# Analysis of the *Trypanosoma brucei* EATRO 164 Bloodstream Guide RNA Transcriptome

**DOI:** 10.1371/journal.pntd.0004793

**Published:** 2016-07-11

**Authors:** Laura E. Kirby, Yanni Sun, David Judah, Scooter Nowak, Donna Koslowsky

**Affiliations:** 1 Department of Microbiology and Molecular Genetics, Michigan State University, East Lansing, Michigan, United States of America; 2 Department of Computer Science and Engineering, Michigan State University, East Lansing, Michigan, United States of America; 3 Merial Veterinary Scholars Program, Michigan State University, East Lansing, Michigan, United States of America; 4 Department of Entomology, Michigan State University, East Lansing, Michigan, United States of America; McGill University, CANADA

## Abstract

The mitochondrial genome of *Trypanosoma brucei* contains many cryptogenes that must be extensively edited following transcription. The RNA editing process is directed by guide RNAs (gRNAs) that encode the information for the specific insertion and deletion of uridylates required to generate translatable mRNAs. We have deep sequenced the gRNA transcriptome from the bloodstream form of the EATRO 164 cell line. Using conventionally accepted fully edited mRNA sequences, ~1 million gRNAs were identified. In contrast, over 3 million reads were identified in our insect stage gRNA transcriptome. A comparison of the two life cycle transcriptomes show an overall ratio of procyclic to bloodstream gRNA reads of 3.5:1. This ratio varies significantly by gene and by gRNA populations within genes. The variation in the abundance of the initiating gRNAs for each gene, however, displays a trend that correlates with the developmental pattern of edited gene expression. A comparison of related major classes from each transcriptome revealed a median value of ten single nucleotide variations per gRNA. Nucleotide variations were much less likely to occur in the consecutive Watson-Crick anchor region, indicating a very strong bias against G:U base pairs in this region. This work indicates that gRNAs are expressed during both life cycle stages, and that differential editing patterns observed for the different mitochondrial mRNA transcripts are not due to the presence or absence of gRNAs. However, the abundance of certain gRNAs may be important in the developmental regulation of RNA editing.

## Author Summary

*Trypanosoma brucei* is the causative agent of African sleeping sickness, a disease that threatens millions of people in sub-Saharan Africa. During its life cycle, *Trypanosoma brucei* lives in either its mammalian host or its insect vector. These environments are very different, and the transition between these environments is accompanied by changes in parasite energy metabolism, including distinct changes in mitochondrial gene expression. In trypanosomes, mitochondrial gene expression involves a unique RNA editing process, where U-residues are inserted or deleted to generate the mRNA’s protein code. The editing process is directed by a set of small RNAs called guide RNAs. Our lab has previously deep sequenced the gRNA transcriptome of the insect stage of *T*. *brucei*. In this paper, we present the gRNA transcriptome of the bloodstream stage. Our comparison of these two transcriptomes indicates that most gRNAs are present in both life cycle stages, even though utilization of the gRNAs differs greatly during the two life-cycle stages. These data provide unique insight into how RNA systems may allow for rapid adaptation to different environments and energy utilization requirements.

## Introduction

The life cycle of *Trypanosoma brucei* involves two distinct environments, the animal host and the insect vector. These environments are distinct in temperature and nutrient composition, providing a unique challenge to *T*. *brucei* as it cycles between hosts. In the bloodstream, trypanosomes exist in two forms, the actively dividing slender form and the non-dividing stumpy form. The slender form is optimized to utilize its glucose rich environment, using glycolysis to generate energy [1]. The stumpy form appears to be transitional, activating mitochondrial genes in preparation for uptake in a blood meal by its tsetse fly vector and subsequent transfer to a harsher environment [1]. Once inside the tsetse fly, the parasite utilizes proline to drive oxidative phosphorylation and ATP production in the mitochondrion [2]. While the activity of the mitochondrion is relatively low during the bloodstream stage (BS), expression of the mitochondrial genome is still essential [3,4]. In *T*. *brucei*, the mitochondrial genome consists of two types of DNA molecules, maxicircles and minicircles. Maxicircles are 22kb circular DNA that contain the genes for two ribosomal RNAs, 12S and 9S, and eighteen mRNA genes [5]. While some of the protein-coding genes do not require RNA editing prior to translation, most require extensive editing before they can be translated [for review see 6,7]. This process involves the insertion of hundreds of uridylates (U)s and less frequently deletion of Us, often doubling the size of the transcript. The sequence changes are guided by small complementary RNA molecules (the guide RNAs) that are encoded on the minicircles [8]. Minicircles make up the bulk of the kinetoplastid network (anywhere from 5,000–10,000 present in each network) with each minicircle encoding 3–5 gRNAs. In *T*. *brucei*, there are more than 200 different minicircle sequence classes (~1200 gRNAs) [8].

Distinct differences in mitochondrial transcript abundance, polyadenylation and the extent of RNA editing are observed during the complex life cycle (Table 1). The pattern of differential RNA editing observed is especially interesting. For example, the cytochrome b (CYb) and cytochrome oxidase II (COII) mRNAs are edited during the insect stage, but are primarily unedited in bloodstream forms [9,10]. In contrast, editing of the NADH dehydrogenase subunit transcripts (ND3, ND7, ND8 and ND9) and editing of the ribosomal protein subunit 12 transcript (RPS12) appears to occur preferentially in bloodstream forms [5,11–15]. Other transcripts, cytochrome oxidase III (COIII) and ATPase subunit 6 (A6) are edited in both life cycle stages [16,17]. Early studies using both Northern blot and primer extension analyses on a limited number of gRNAs indicate that gRNAs are present in both insect and bloodstream forms, suggesting that the regulation of RNA editing is not at the level of gRNA availability [13,18,19]. Our lab has previously published deep sequencing results of the gRNA transcriptome of the *T*. *brucei* EATRO 164 procyclic form [20]. Here we present the deep sequencing data for the gRNA transcriptome of a bloodstream form of EATRO 164. A total of 211 populations of gRNAs were identified. We define a population as a group of gRNAs that may vary in sequence, but direct the editing of the same or near same region of the mRNA. Because kinetoplastid RNA editing allows G:U base pairing, most populations contain multiple sequence classes that can guide the generation of the same mRNA sequence. While the number of populations identified was similar to the number identified in the procyclic gRNA transcriptome (214 populations), the total number of gRNAs identified was much reduced and the coverage was less complete; full complements of gRNAs were only identified for COIII and CYb. In spite of the reduced number of gRNAs, an interesting correlation was found that suggests a relationship between the relative abundance of initiating gRNAs between stages and the developmental pattern of mRNA editing.

**Table 1:** Differences in mitochondrial transcript abundance, polyadenylation and the extent of RNA editing in two life cycle stages of *T*. *brucei*.

	No. of uridines				Relative level of mature RNA			PolyA tail length			
Gene	Added	Deleted	Edited size (nt)	Stage Edited	Long Slender	Short Stumpy	Procyclic	Bloodstream	Procyclic	Number of PC major classes^g^	References
12S	2–17 (tail)	0	1149	N.D.	0.04	1.3	1.0	N.A.	N.A.	N.A.^j^	[9,21,22]
9S	7 (tail)	0	611	N.D.	0.07	1.4	1.0	N.A.	N.A.	N.A.	[9,21,22]
CYb	34	0	1,151	P^a^	~0	0.5	1.0	Short (UE^h^ & E^i^)	Short (E) & Long (E)	11	[9,10,23,24]
A6	448	28	821	P/BS^b^	1.0	N.D.^f^	1.0	Short (E) & Long (E)	Short (E) & Long (E)	81	[16,23]
COI	0	0	1,647	NE^c^	0.07	0.4	1.0	Short	Short & Long	N.A.	[9,24,25]
COII	4	0	663	P	~0	0.5	1.0	Short	Short (UE) & Long (E)	N.A.^k^	[9,24,26]
COIII	547	41	969	P/BS	1.0	N.D.	1.0	Short (E)	Short(E) & Long(E)	151	[17]
ND1	0	0	960	NE	~1	N.D.	~1	Short & Long	Short & Long	N.A.	[25,27,28]
ND2	0	0	1343	NE	>1.0	N.D.	1.0	Short & Long	Short & Long	N.A.	[24,29,30]
ND3	210	13	452	P/BS	>1.0	N.D.	1.0	Short (E)	Short (E)	34	[11]
ND4	0	0	1314	NE	~1.0	N.D.	~1.0	Short & Long	Short & Long	N.A.	[24,25,31,32]
ND5	0	0	1,779	NE	0.5	0.8	1.0	N.D.	N.D.	N.A.	[9,25]
ND7	553	89	1,238	5’P/BS, 3’BS^d^	~10	N.D.	1.0	Short(E)	Short(UE)	129	[5,12]
ND8	259	46	574	BS^e^	~20	N.D.	1.0	Short (E) & Long (E)	Short (E)	70	[5,13,24]
ND9	345	20	649	BS	>1.0	N.D.	1.0	Short (PE) & Long (E)	Short (PE)	39	[14]
RPS12	132	28	325	BS	>1.0	N.D.	1.0	Short(UE & E) & Long (E)	Short(UE & E) & Long (E)	50	[15]
Murf 2	26	4	1,111	P/BS	~1	~1	~1	Short & Long	Short & Long	1	[27,33]
Murf 5	N.D.	N.D.	N.D.	N.D.	N.D.	N.D.	N.D.	N.D.	N.D.	N.A.	N.D.
CR3	148	13	299	BS	>1.0	N.D.	1.0	N.D.	N.D.	37	[34]
CR4	325	40	567	BS	1.0	N.D.	~0	Short (E) & Long (E)	Short (UE)	41	[35]

^a^P, transcript is edited only in the procyclic (insect) developmental stage.

^b^P/BS, transcript is edited in both bloodstream and procyclic stages.

^c^NE, never edited, editing of these transcripts has not been reported.

^d^The ND7 transcript is differentially edited in the procyclic and bloodstream stages.

^e^BS These transcripts are only fully edited in the bloodstream developmental stage [36].

^f^N.D. Values have not yet been determined.

^g^All data comes from EATRO 164 procyclic gRNA transcriptome previously published [20].

^h^UE, unedited, the transcripts which carried these tails were typically unedited.

^i^E, edited, the transcripts carrying these tails were typically edited.

^j^N.A., Not applicable.

^k^COII is a cis-edited transcript. Poly-A tails listed as short are between 10 and 50 nts long and tails listed as long are between 150 and 200 nts long.

## Materials and Methods

### Parasites, isolation of mitochondria and RNA extraction

*T*. *brucei brucei* clone IsTar from stock EATRO 164 were grown in rats and isolated as previously described [37]. Bloodstream forms were virtually all long-slender forms isolated after 4 days of infection. Parasites were used immediately for isolation of mitochondria using differential centrifugation as previously described or stored frozen at -80°C until RNA extraction [20]. Both total RNA from whole parasites and mitochondrial RNA (mtRNA) from purified mitochondria were isolated by the acid guanidinium-phenol-chloroform method [38].

### Ethics statement

Rats were raised according to the animal husbandry guidelines established by Michigan State University. All vertebrate animal use procedures were approved by MSU’s Institutional Animal Care and Use Committee (Application 03/11-051-00). MSU has filed with the Office of Laboratory Animal Welfare (OLAW) an assurance document that commits the university to compliance with NIH policy and the Guide for the Care and Use of laboratory Animals.

### Library preparation and Illumina sequencing

Samples of mtRNA and total RNA were both treated with DNAse RQI and size fractioned on a polyacrylamide gel as previously described [20]. Guide RNAs were extracted from the gel and prepped for sequencing using the Illumina ‘Small RNA’ protocol as previously described [20]. Libraries from both mtRNA and total RNA samples were deep sequenced on Illumina GAIIx. Reads were then processed and trimmed as previously described [20]. Data with two or more Ns, shorter than 20nts after trimming or with an overall mean Q-score < 25 were discarded. Redundant reads were then removed, while maintaining the number of redundant reads and reads containing fewer than 4 consecutive Ts were removed.

### Identification of gRNAs

To identify gRNAs, each transcript read was aligned to the conventionally edited mRNAs based on known base pairing rules (canonical Watson-Crick base pairs and the G-U base pair). In the initial screen, no gaps were allowed in the alignment, allowing the formulation of the gRNA-mRNA alignment as an extended longest common substring (LCS) problem as previously described [10]. Matched gRNAs were then scored (two points for G:C and A:U base pairs and one point for G:U base pairs). gRNAs with scores >45 were identified as guiding a specific region based on the identified mRNA fully edited sequence. Additional searches with reduced stringency (scores >30) were performed on regions with low gRNA coverage. The matched gRNAs were sorted into populations based on their guiding positions, and the populations analyzed and sorted into major sequence classes.

## Results

Much of the initial characterization of RNA editing in *T*. *brucei* was done using the EATRO 164 strain. These experiments suggested that RNA editing was developmentally regulated in that certain genes were shown to be more fully edited in some stages than others (Table 1)[9–17,33]. It was also reported that the developmental regulation was not controlled by gRNA availability, as gRNAs were found in both life cycle stages [13,18,19]. In these early studies, however, only a small number of gRNAs were investigated. In this study, we used deep sequencing to compare the gRNA transcriptomes of a bloodstream form to a procyclic form of *T*. *brucei* EATRO 164. The EATRO 164 strain was isolated in 1960 from *Alcephalus lichtensteini* and maintained in the lab of Dr. K. Vickerman until being obtained by Dr. Stuart in 1966 [39]. Dr. Stuart derived the procyclic form from the Bloodstream culture in 1979 [39]. Both cell lines have been maintained in separate culture since that time.

Trypanosomes from the EATRO 164 strain were grown in Wistar rats to a parasitemia of 1–2 x 10^9^ trypanosomes per mL and isolated using DEAE cellulose columns. Mitochondria and gRNAs were purified as previously described [20]. Libraries were generated using gRNAs isolated from whole cell RNA and gRNAs isolated from mitochondrial RNA. Both bloodstream gRNA libraries were searched using conventionally accepted fully edited mRNA sequences, and a total of 1,024,604 gRNA reads were identified. Surprisingly, the library generated using gRNAs isolated from whole cell RNA had more than twice as many identified gRNA reads as the data generated using gRNAs isolated from mitochondrial RNA. To insure sufficient abundance and gRNA coverage, the two data sets were combined for the analyses presented here. In contrast, over 3 million gRNA reads were identified in our procyclic gRNA transcriptome generated from gRNAs isolated from mitochondrial RNA. Of the 1,024,604 reads identified from the bloodstream transcriptomes, 982,450 reads were sorted into major sequence classes.

**Fig 1:**
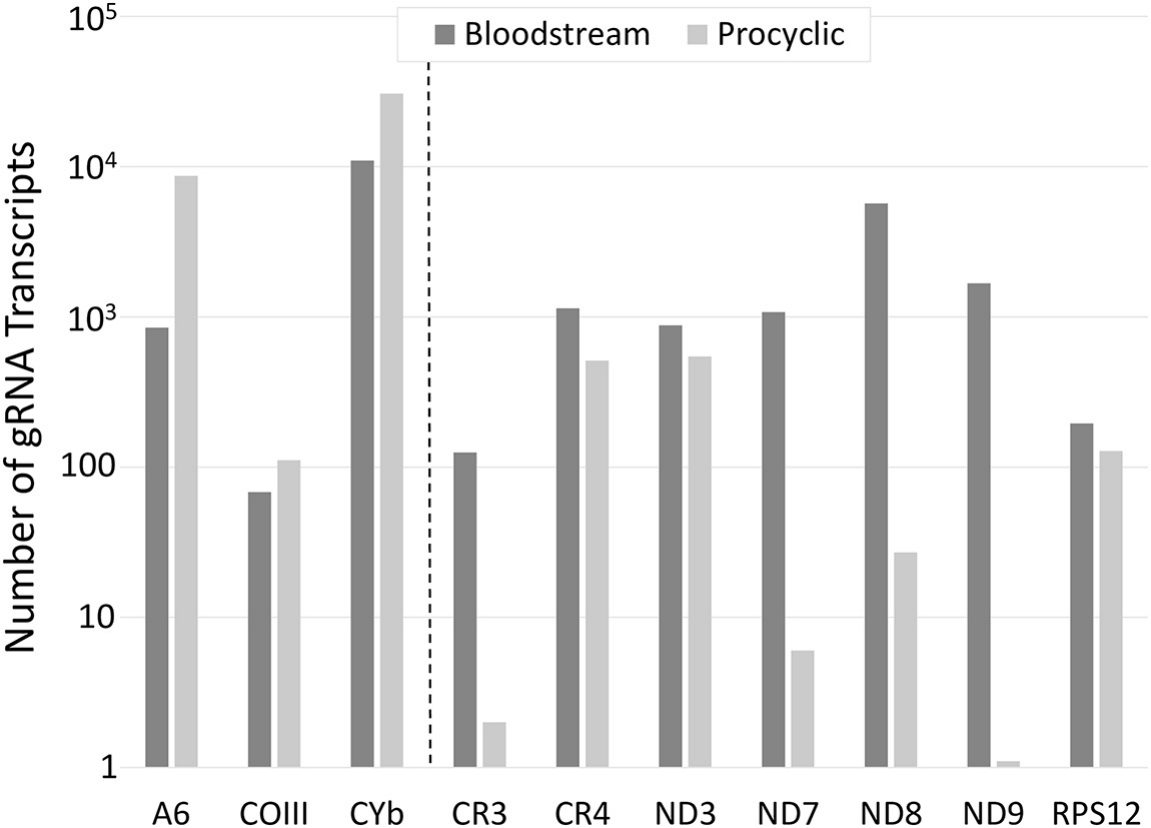
The abundance of the initiating gRNA of all edited mRNAs in each stage. mRNAs to the left of the dashed line are constitutively edited or are edited only in the procyclic stage [16,17,27,33]. mRNAs to the right of the dashed line are only fully edited or more abundant fully edited in the bloodstream stage [11–15].

The overall ratio of identified procyclic gRNA reads to BS gRNA reads was 3.5:1. This ratio varies significantly by gene (Table 2), and by populations within genes (S1 Fig) and, except for the initiating gRNA, no apparent trend relating gRNA abundance and developmental editing pattern was observed. Interestingly, for the initiating gRNA, mRNAs that are fully edited in the procyclic stage only, or are fully edited in both life cycle stages had initiating gRNAs with more reads in the procyclic data set (Fig 1) [16,17,27,33]. In contrast, mRNAs that are only fully edited or are more abundant in the BS, had more initiating gRNAs reads in the BS data set (Fig 1) [11–15].

**Table 2:** Number of gRNA transcripts in procyclic and bloodstream major classes and ratio of procyclic transcripts to bloodstream transcripts for each gene.

Gene	Bloodstream gRNA Reads	Procyclic gRNA Reads	Ratio of PC to BS Reads
A6	41,628	266,532	6.40
COIII	371,139	948,845	2.56
CR3	13,316	236,808	17.78
CR4	25,753	51,979	2.02
CYb	11,022	31,622	2.87
MurfII	157	2,605	16.59
ND3	13,567	75,739	5.58
ND7	291,927	702,061	2.40
ND8	112,868	584,639	5.18
ND9	83,924	72,027	0.86
RPS12	17,191	403,131	23.45
Total	982,492	3,375,988	3.44

Because the identified gRNAs from the BS cells were less abundant, the rule used to identify major gRNA sequence classes was relaxed. Instead of using a strict cut off for the minimum number of reads required, the cut off was assessed on a case-by-case basis. For example, if the total population only had 100 reads, a sequence class with only 10 reads would still be identified as a major sequence class. Once all major classes were identified, 657 sequence classes were identified that could be sorted into 211 populations (Table 3). Although the overall gRNA numbers were down in comparison to the procyclic data set, most of the populations found in that stage (214 gRNA populations) were also identified in the BS transcriptome. However, there were a number of populations that were unique to either the procyclic or BS stage.

**Table 3:** Summary of the gRNA data coverage for each gene.

	Populations		Unique Populations		Average^a^ gRNA Overlap (nts)		Gaps		Weak Overlaps	
Gene	BS	PC	BS	PC	BS	PC	BS	PC	BS	PC
A6	29	28	1	0	18	20	1	0	0	0
COIII	42	39	4	1	19	22	0	0	0	0
CR3	9	9	0	0	19	14	1	0	0	2
CR4	16	18	0	2	17	18	2	0	0	0
CYb	2	2	0	0	12	14	0	0	0	0
MurfII	1	1	0	0	N.A.	N.A.	1	1	0	0
ND3	12	12	0	0	15	15	1	1	0	0
ND7	45	48	2	5	17	21	7	2	4	1
ND8	20	21	2	3	17	21	2	1	1	0
ND9	24	23	1	0	16	16	1	0	0	2
RPS12	11	13	0	2	17	21	2	0	1	0
Total	211	214	10	13	17	19	18	5	6	5

^a^The average gRNA overlaps were determined excluding any regions where neighboring gRNAs shared no overlap.

**Fig 2:**
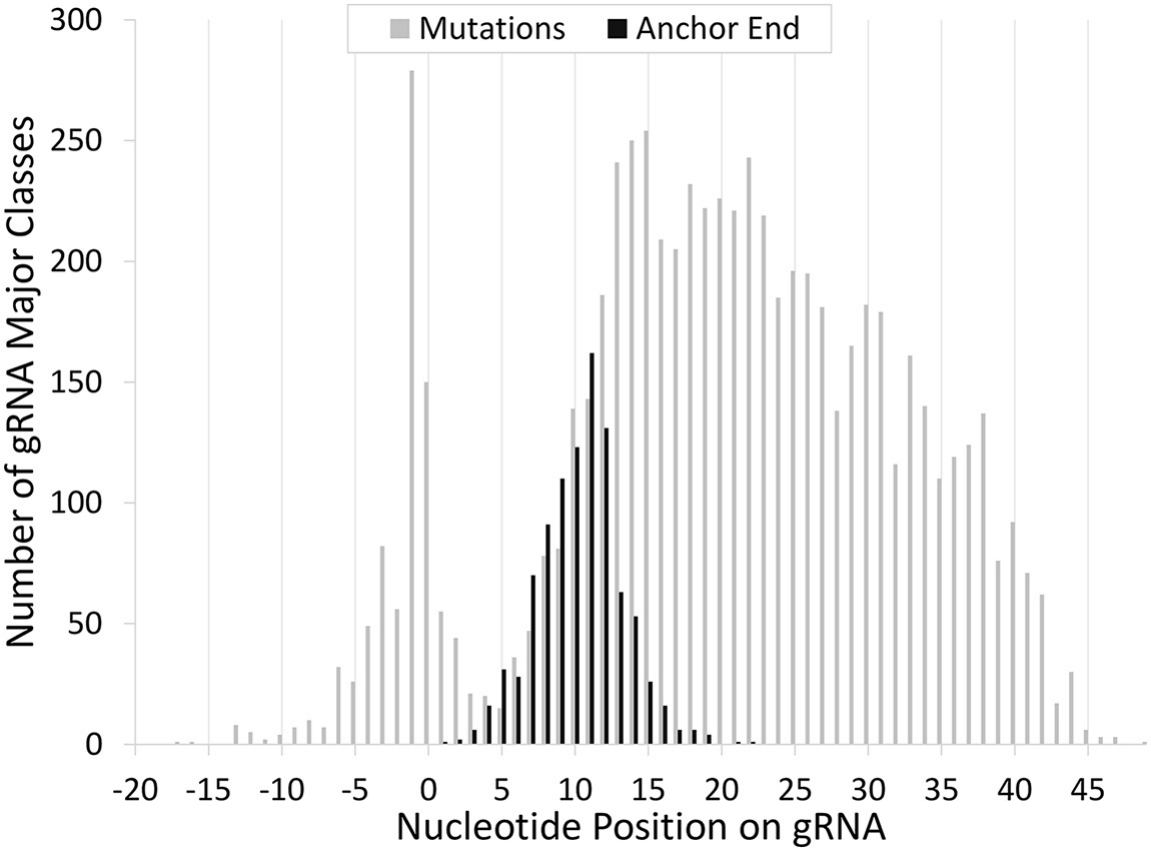
The frequency of nt variations versus nucleotide position in the gRNA. Gray bars indicate the number of gRNA sequence classes with an identified nt difference between related procyclic and bloodstream gRNAs. Nucleotide numbering for each gRNA was normalized by setting the start of the Watson-Crick anchor region to zero. Black bars indicate the number of gRNA sequence classes whose contiguous Watson-Crick anchors end at that position (start of Watson-Crick = zero, so this is an indication of the length of the contiguous Watson-Crick region).

Surprisingly, when the bloodstream and procyclic data sets were compared, only 37 identical major sequence classes were found in both. However, distinctly related sequence classes could be identified when comparing the BS and procyclic populations. Comparing the related major classes from each transcriptome (BS vs procyclic) revealed a median value of ten single nucleotide variations per gRNA. Interestingly, nt variations were much less likely to occur in the consecutive Watson-Crick anchor region of the gRNA than in the rest of the gRNA indicating a very strong bias against G:U base pairs in this region (Fig 2).

**Fig 3:**
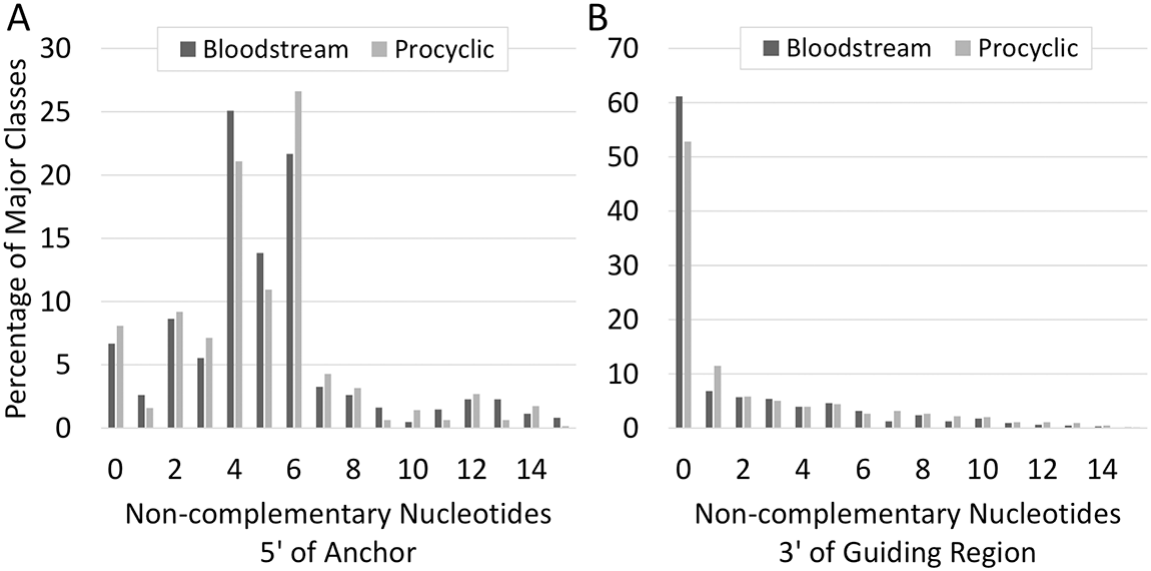
Comparing the number of non-complementary nucleotides 5’ of the anchoring region (A) or 3’ of the guiding region (excluding the U-tail) (B) in procyclic and bloodstream gRNAs.

**Fig 4:**
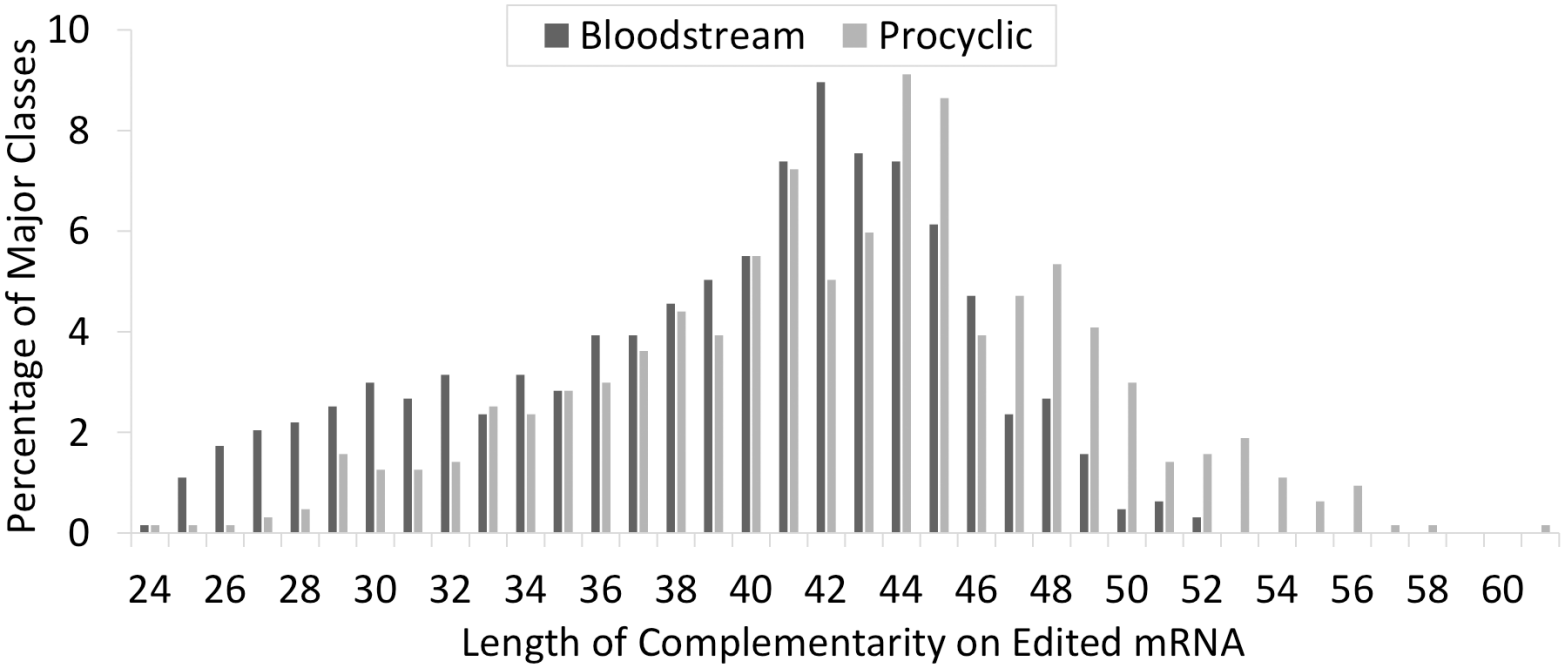
Length of gRNA complementarity (including anchors) to fully edited mRNAs for both bloodstream and procyclic gRNAs.

The Watson-Crick anchors (defined as the number of consecutive nts in the 5’ region with only G:C and A:U base pairs) had a median length of eleven nucleotides and anchor length did not vary between the two forms. The vast majority of major classes of gRNAs had consecutive Watson-Crick anchors greater than seven nts long (92.5%). In addition, most gRNAs with Watson-Crick anchors shorter than eight nts were not an abundant major class for their respective populations. Consistent with observations made from the procyclic data set, most gRNAs had zero non-base pairing nucleotides 5’ to the poly-uridine tail and 4 to 6 non-base pairing nucleotides 5’ to the anchor region (Fig 3). Also consistent with procyclic data, most of the gRNAs (59%) had 38 to 48 nts of complementarity (including anchor regions) with their respective mRNAs (Fig 4). Transcription start sites also did not vary, as preference for an RYAYA start site was observed (Table 4).

**Table 4:** Most common gRNA transcription start sites in procyclic and bloodstream data.

	Percentage of Sequence Classes		Percentage of Transcripts	
Initiating Sequence	Bloodstream	Procyclic	Bloodstream	Procyclic
ATATAT	32.20%	35.20%	33.60%	37.40%
ATATAA	20.00%	21.10%	17.90%	24.70%
AAAAAA	3.60%	4.60%	1.60%	1.30%
ATATAC	3.80%	4.30%	1.50%	3.80%
ATACAA	2.40%	2.80%	1.30%	1.70%
ATATTA	4.90%	2.60%	13.40%	0.90%
ATATAG	2.20%	2.60%	0.70%	7.60%
ATAAAT	2.50%	2.60%	1.00%	0.70%
ATACAT	2.70%	2.20%	3.20%	2.20%
ATAAAA	1.70%	2.10%	0.20%	1.10%

### Coverage and gaps

**Fig 5:**
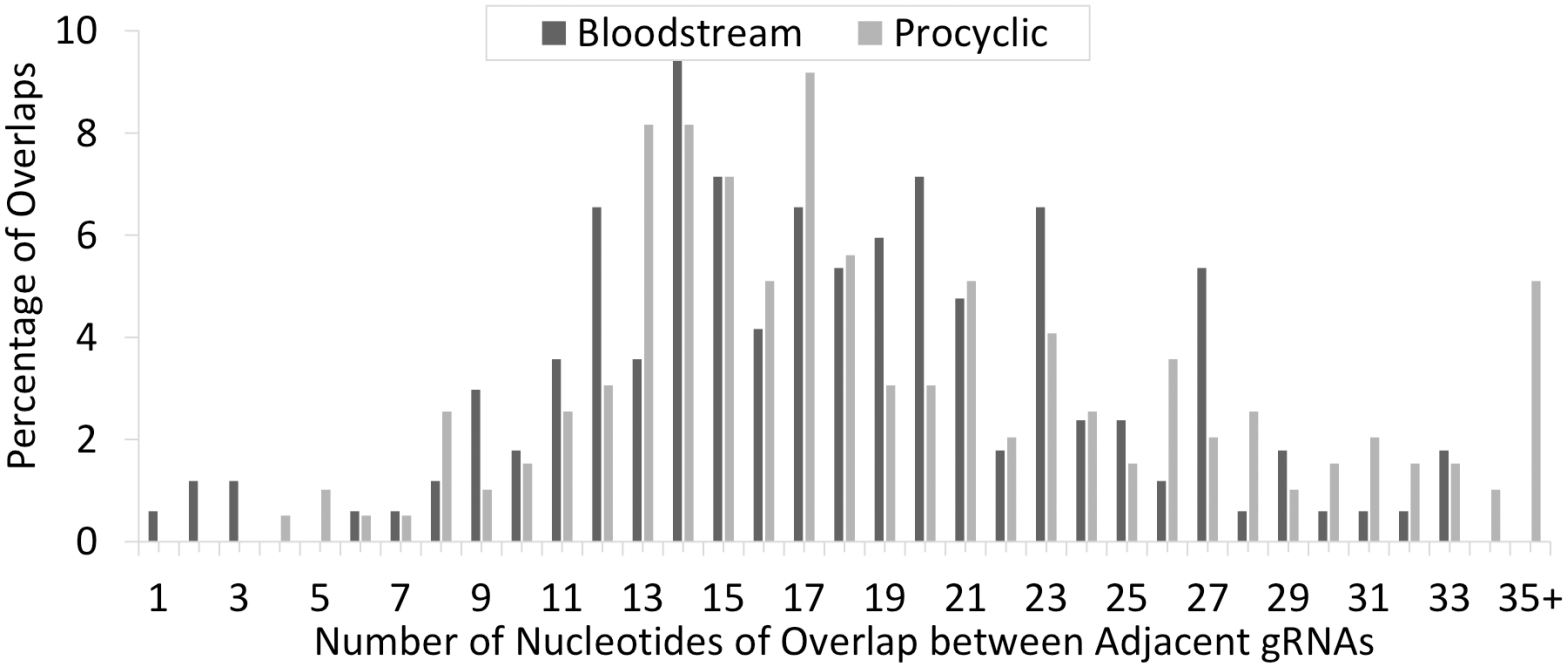
The percentage of different nucleotide overlaps found between adjacent gRNAs. gRNAs were aligned to their fully edited mRNA sequence and the number of mRNA nts with complementarity to both adjacent gRNAs determined.

In order to determine if the BS gRNA transcriptome contained a full complement of guide RNAs, the gRNA populations were aligned to the fully edited mRNAs (S2 Fig). We note, that for an mRNA to be fully edited, not only must all editing sites on the mRNA be covered by a gRNA, the downstream gRNA must generate the anchor binding site for the subsequent gRNA. Therefore, adjacent gRNAs must overlap. Overall, there was an average of 17 nts of overlap between adjacent gRNAs, with the average overlap varying slightly by gene (Table 3). As the median Watson-Crick Anchor is 11 nts, in most cases, the overlap extends beyond the Watson-Crick anchor of the subsequent gRNA. However, we did observe a number of regions where the overlap is minimal. Currently, there is no data that stipulates the minimum anchor needed for efficient editing. However, we postulate that similar to microRNAs, for an anchoring sequence to be sufficiently specific, it should be at least six nucleotides [40]. Indeed, when examining the overlaps between most gRNAs, there are only ten (four procyclic and six BS) that are less than six nucleotides (Fig 5).

We therefore used six nucleotides as a cut off to identify regions with potential missing guide RNAs for both life cycle stage transcriptomes. In contrast to the procyclic data, where full complements of gRNAs were identified for five of the mRNA transcripts (A6, COIII, CR4, CYb, and RPS12), in the BS transcriptome, a full complement of gRNAs was only identified for COIII and CYb. Overall, there are 12 edited regions where no gRNAs were identified, and five regions with weak gRNA overlaps in the BS data (Table 5). Of these 17 regions, seven belong to ND7 alone. Interestingly, nine of the 17 missing populations are in very low abundance in the procyclic data, having 100 or fewer reads. Because the number of reads in the BS data is ~3.5 fold less abundant, this could account for some of these regions of poor coverage. There are six regions that lack gRNA coverage in both data sets. These are found in CR3, MurfII, ND3 and ND7 (Table 5). Interestingly, three of these regions are close to the 3’ end of their respective genes. Regions of weak overlap (ND9(238–242), ND9(609–612)) and regions without gRNA coverage (CR3(278–292), ND8(541–553)) that are unique to the procyclic transcriptome were also observed. Interestingly, the regions of poor procyclic coverage are found in CR3, ND8 and ND9, all transcripts that are preferentially edited in the BS form [5,13,14,34].

**Table 5:** Identified gaps or weak overlaps (less than 6 nucleotides) between populations of gRNAs observed in both data sets.

Gene	Stage Missing Coverage	Range^b^	Gap or Overlap	Abundance of Equivalent gRNA
A6	Bloodstream	669–670	2 nt Gap	39,063
CR3	BS/P^a^	233/226-230	1 nt G/5 nt O	Missing in Both Stages
CR3	Procyclic	278–292	15 nt Gap	125
CR4	Bloodstream	143–165	23 nt Gap	7,175
CR4	Bloodstream	302–306	5 nt Gap	643
MurfII	BS/P	80–85	6 nt Gap	Missing in Both Stages
ND3	BS/P	389–401	13 nt Gap	Missing in Both Stages
ND7	BS/P	92–94	3 nt Gap	Missing in Both Stages
ND7	Bloodstream	95–120	26 nt Gap	1
ND7	Bloodstream	292–293	2 nt Overlap	3,259
ND7	Bloodstream	325–326	2 nt Gap	888
ND7	BS/P	485–486	0 nt Overlap	Missing in Both Stages
ND7	Bloodstream	1000–1000	1 nt Overlap	101
ND7	Bloodstream	1079–1085	7 nt Gap	44
ND7	BS/P	1086/1086-1088	1 nt G/3 nt G	Missing in Both Stages
ND7	Bloodstream	1225–1232	8 nt Gap	123
ND7	Bloodstream	1269–1270	2 nt Overlap	63
ND8	Bloodstream	54–56	3 nt Overlap	1
ND8	Bloodstream	153–159	7 nt Gap	4
ND8	Bloodstream	386–389	4 nt Gap	2
ND8	Procyclic	541–553	13 nt Gap	413
ND9	Procyclic	238–242	5 nt Overlap	652
ND9	Bloodstream	340–342	3 nt Gap	36
ND9	Procyclic	609–612	4 nt Overlap	7
RPS12	Bloodstream	122–132	11 nt Gap	3
RPS12	Bloodstream	156–158	3 nt Overlap	62
RPS12	Bloodstream	337–349	13 nt Gap	128

^a^Indicates that the region of poor coverage was identified in both data sets.

^**b**^Range is respective nucleotides in the fully edited mRNA. Both nucleotides and deletion sites in the fully edited mRNA were numbered starting from the 5’ end (see S2 Fig for numbered sequences).

While the number of reads in the BS data is less abundant than the procyclic data in general, there are 87 gRNA populations with more identified reads than in the procyclic data set (Table 6). These populations are found in every gene except CYb and MurfII, but most of the populations belong to one of the NADH dehydrogenase subunits, particularly, ND7 or ND9. Another trend worth noting is that the all of the genes with fully edited transcripts that are more abundant in the BS stage, (CR3, CR4, ND3, ND7, ND8, and ND9, with the exception of RPS12), have a higher percentage of classes that are more abundant in the BS (Table 6).

**Table 6:** Summary of populations found in both data sets that have more reads in the bloodstream data set than in the procyclic data set.

Gene	PC and BS Shared Populations	Populations more abundant in BS	Percentage of populations more abundant in BS
A6	28	6	21%
COIII	38	12	32%
CR3	9	5	56%
CR4	16	10	63%
CYb	2	0	0%
MurfII	1	0	0%
ND3	12	6	50%
ND7	43	20	47%
ND8	18	8	44%
ND9	23	16	70%
RPS12	11	4	36%
Total	201	87	43%

### Gene specific gRNA characteristics

#### ATPase 6

In the BS gRNA transcriptome, a total of 29 gRNA populations containing 86 different major sequence classes were identified that could guide the editing of A6 (Table 3; S1A Table). One population was identified that was unique to the BS transcriptome (gA6(281–329)). The gRNAs bordering this population share extensive overlap, so its absence in the procyclic transcriptome would not impact the editing process (S1A Table). We note that two of the gRNAs identified have single nucleotide mismatches. The bloodstream gA6(640–668) has an identified mismatch (C:U) that disrupts the complementarity of the gA6(640–668) population (S2A Fig). The second mismatched gRNA (gA6(520–533)) would introduce a frameshift. Excluding these two mismatched regions, there is complete coverage of ATPase 6. In contrast to the procyclic data, where the conventional initiating gRNA and the gRNA immediately following it were extremely rare, both of these gRNAs, gA6(773–822), previously identified as gA6-14 and gA6(745–789), were fairly abundant, each having hundreds of reads. The alternative initiating gRNA identified in the procyclic data set was not found. This finding is similar to that found in the *T*. *brucei* Lister strain 427 where authors identified alternative initiating gRNAs not found in the EATRO 164 procyclic gRNA transcriptome [41].

**Fig 6:**
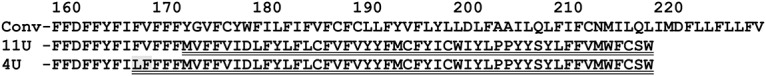
Alignment of conventional ATPase 6 protein sequence to hypothetical proteins generated by the 11U alternative edited mRNA and the 4U alternatively edited mRNA. Double underlined residues show where the alternative sequences differ from the conventional sequence. The shaded residues in the 4U sequence show where it differs from the 11U sequence.

Another disparity between the two life cycle data sets was found when comparing the abundance of gRNAs implicated in a potential alternative edit. In the procyclic gRNA transcriptome, a gRNA was identified that would guide the insertion of 11 U-residues instead of the needed 12 between G555 and A568 [20]. This gRNA (pA6(557–593)) was 25-fold more abundant than the conventional gRNA (pA6(549–593)). In the BS data set however, more than 400 reads of the 12U gRNA were identified and only one read was found that would encode the alternative 11U edit. Surprisingly, while G555-A568 would be correctly edited (insertion of 12Us), the next editing site (A549-G555) is edited by bsA6(520–553), the gRNA that introduces the 1 nt frameshift. This frameshift would generate a predicted protein with nearly the same amino acid sequence as the procyclic 11U frameshift edit (two amino acid changes) (Fig 6).

#### Cytochrome oxidase subunit III

**Fig 7:**
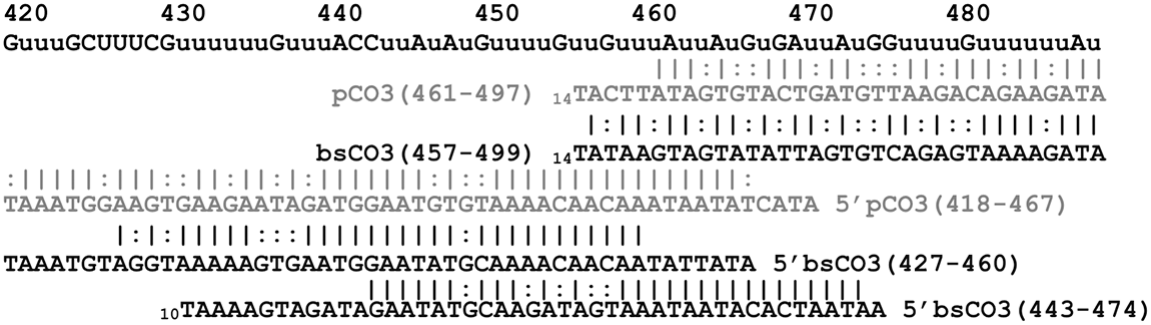
Editing sites 420–489 of COIII aligned with the gRNAs identified for that region in the procyclic (grey) and bloodstream (black) data sets. The gRNA covering 443–474 was only found in the bloodstream data set.

Forty-two gRNA populations, guiding the editing of COIII were identified in the BS transcriptome; three more than in the procyclic data set (S1B Table). This disparity is caused by the presence of several unique populations. While the procyclic data set contained one unique population, the BS data contained four gRNA populations not previously identified. Of these four unique populations, three of them are required for full overlapping coverage in the bloodstream. They are not however, required for full coverage in the procyclic stage. These three unique gRNA populations all span relatively small regions of weak overlap (Fig 7, S2B Fig).

An alternative edit of COIII has been described, involving distinct edits at two adjacent sites that links the open reading frame of the edited 3’ end to an ORF found in the 5’ pre-edited sequence [42]. The previously identified alternative gRNA that can generate the needed editing events was not found in either the BS or procyclic transcriptomes.

#### C-rich regions 3 and 4

In the BS data set, nine populations and 34 major sequence classes were identified that direct the editing of the CR3 transcript. The coverage of edited CR3 is nearly complete in the bloodstream data set with only a one nt gap in coverage (editing site 233) (S2C Fig). This is in contrast to the procyclic transcriptome, where gRNAs that matched the published sequence downstream of nt 196 were very rare (<10 copies) and no gRNAs were identified that could direct editing near the 3’ end (nucleotides 275–292).

A full consensus sequence for edited CR4 has only been found in BS *T*. *brucei* [35]. Using this sequence, 16 gRNA populations, containing 62 major sequence classes were identified in the BS transcriptome (S1D Table). In contrast to the procyclic data, where a full complement of gRNAs were identified, there are two gaps in the BS coverage (Table 5).

#### Cytochrome b and maxicircle unidentified reading frame II

RNA editing in the Cytochrome b (CYb) transcript is limited to the 5’ end and two gRNA populations are sufficient to guide the small number of edits needed to render the CYb transcript functional. Both populations were observed in both data sets, with a total of 6 major classes. Interestingly, in both data sets, the initiating gRNA is significantly more abundant than the second gRNA, being approximately 30 fold more abundant in the procyclic data set and approximately 200 fold more abundant in the bloodstream data set (S1E Table). This is in contrast to most of the other transcripts where the initiating gRNAs are not very abundant. In addition, almost all of the CYb gRNA major classes have an A-run transcription start site, deviating from the common RYAYA initiation site pattern.

Editing in MurfII is also limited to the 5’ end and requires only two gRNAs. One of these gRNAs (gMurfII(30–79)) is encoded on the maxicircle [43]. While this gRNA was observed in both data sets, the gRNAs identified were not identical. A purine-purine transition near the 3’ end of the gRNA differentiates the procyclic and BS forms (S1F Table and S2F Fig). An initiating gRNA is needed to generate the 3’ most edits that create the anchor sequence for gMurfII(30–79). This gRNA was not found in either data set, despite additional searches with reduced search stringency.

#### NADH dehydrogenase subunits 3, 7, 8, and 9

In the initial characterization of RNA editing in *T*. *brucei* EATRO 164, fully edited ND subunit transcripts were only found in RNA isolated from the BS stage. We were therefore surprised to find that fewer ND gRNA populations were identified in the BS transcriptome and a full complement of gRNAs was not identified for any of the ND subunits. The most complete coverage was found for ND3 and ND9. For ND3, the BS data set contained twelve populations and 41 major classes of gRNAs. One gap in coverage was observed, from 389–401. This region overlaps a region that has no clear consensus sequence, 375–395 [11]. ND9 is the only gene in this study whose bloodstream gRNA reads outnumber the procyclic gRNA reads identified (Table 2). Twenty-four bloodstream gRNA populations were identified with all edited nucleotides covered if gRNAs with a single base pair mismatch are taken into account (S2J Fig).

While 45 gRNA populations were identified for ND7 in the BS data set, the gRNA coverage was significantly worse when compared to the identified procyclic gRNAs (Table 5). Despite the poor coverage, two unique gRNA populations (bs gRNA (772–816) and (1128–1182)) were identified (S2H Fig and S1H Table). ND8 also had poor gRNA coverage (Table 5). Interestingly, there are several populations in ND8 that contain highly abundant gRNA sequence classes with mismatches that shorten the complementarity of the gRNA. These usually have a single mismatch in the gRNA that would otherwise guide conventional editing (S1I Table).

#### Ribosomal protein S12

The BS data set contained 11 populations and 26 major sequence classes that direct editing of RPS12 (Table 3). While the procyclic transcriptome contained a full complement of gRNAs, the BS RPS12 data contains one gap in coverage and one region of poor overlap, (Table 5). This was surprising, as RPS12 has been shown to be essential in both life cycle stages [44,45]. The region of the mRNA with poor coverage has a high percentage of C residues and gRNAs covering this region may utilize C:A base pairs. If this is the case, some classes of gRNAs may not have been detected, as the program used to search for gRNAs does not allow for C:A base pairs (S2K Fig).

## Discussion

This is the first comprehensive characterization of the mitochondrial gRNA transcriptome from the bloodstream stage of *Trypanosoma brucei brucei*. As we have previously characterized the insect stage gRNA transcriptome, these data allow the comparison of gRNA characteristics across the two main life cycle stages [20]. In the EATRO 164 BS gRNA transcriptome, gRNAs for every edited gene were identified. Interestingly, while the number of populations identified in this data set was only slightly lower than that reported in the procyclic data set, the total number of gRNA transcript reads identified was considerably lower despite the fact that multiple transcriptome libraries were combined. While this may be a reflection of the down regulation of mitochondrial transcription in the bloodstream stage (see Table 1), it is impossible to rule out technical problems in the generation and sequencing of the libraries. It has been previously reported that gRNA presence did not correlate with developmental RNA editing patterns in *T*. *brucei* and our data does not challenge this [18,19]. The data did however, show an interesting trend in the abundance of the initiating gRNAs as relates to their developmental editing patterns (Fig 1). It may be that the abundance of the initiating gRNAs is regulated in order to control editing of their target mRNAs. However, we cannot rule out the possibility that not all of the populations of initiating gRNAs were identified. For the pan-edited mRNAs, the initiating gRNAs direct sequence changes that are often downstream of the stop codon. Sequence changes in this region would be tolerated, as long as the anchor sequence for the next gRNA is maintained. This type of mutation was observed in the 3’ end of ATPase 6 [2]. In addition, characterization of the initiating gRNAs in the Lister 427 *T*. *brucei* cell line identified several gRNAs that would direct an alternative editing pattern, suggesting a high tolerance for sequence changes near the mRNA 3’ ends. [41].

As expected, general gRNA characteristics are conserved across the two life-cycle stages. Populations retain the general location of their anchors, there is relatively little shift in the location of populations, and the lengths of complementarity are very similar. We did observe that considerable nucleotide variations were found in the guiding regions of the gRNAs from the different life cycle strains of the EATRO 164 cells. This particular cell line dates back to 1960 when the BS form was originally acquired [39]. Procyclic cells were derived from the BS stock in 1979 and the two cell lines maintained separately since that date [39]. Mixed trypanosome genotypes are detected frequently in field isolates from both tsetse flies and mammals and it may be that separation into different culture conditions allowed different genotypes to predominate in each life cycle strain [46–48]. Because gRNAs utilize both canonical (Watson-Crick) as well as G:U base-pairing to direct the change in sequence, most transition mutations in the gRNA, would not lead to changes in the mRNA sequence and would not be selected against [49]. We do note however, that a very strong bias against A to G transitions is observed in the anchor regions of the gRNAs. This suggests that transition mutations in this region are not tolerated. This suggests that the editing machinery recognizes and selects for a conventional base-paired double helix in the initial gRNA/mRNA pairing. The ability to discriminate against G:U base-pairs in the initial interaction would greatly increase the accuracy of the gRNA targeting event. Considering the sequential nature of the overall editing process, this would be very advantageous.

### Coverage

Surprisingly, complete gRNA coverage was observed only for the pan-edited COIII and for CYb, where editing is limited to the 5’ end. The identification of the CYb gRNAs was expected, as it has been previously reported that the gRNAs are present in both life cycle stages even though editing of CYb is limited to the procyclic stage [8, 9]. The full coverage of COIII was also not surprising, as COIII was shown to be fully edited and equally abundant in both stages [17]. However, we expected to see complete coverage of ATPase 6 and RPS12 as both of these transcripts have been shown to be essential in both life cycle stages [3,44,45,50]. For ATPase 6, we did identify a total of 29 gRNA populations that do cover all of the editing sites. However, one of the gRNAs (bsA6(643–667)) has a single nucleotide mismatch (C:U) and one would introduce a frameshift (bsA6(520–553)). The C:U mismatch occurs near the middle of the gRNA, placing the C:U mismatch in a region that is unusually high in Gs and Cs (S2A Fig). It may be that the G:C basepairs immediately upstream of the mismatch stabilize the gRNA/mRNA interaction, allowing it to be tolerated. The frameshift gRNA is also interesting, as it occurs just upstream (1 editing site) of another site where we had previously observed a frameshift sequence anomaly. Both frameshifts (the BS 4U and the Procyclic 11U) generate a predicted protein with nearly the same amino acid sequence. As the frameshifts occur downstream of the highly conserved amino acid region involved in proton translocation [16], it may be that this different carboxyl terminus is tolerated.

Near full coverage is also observed for RPS12. For this transcript, one BS identified gRNA (bsRPS12(96–121)) has an A-nt insertion that disrupts the gRNA complementarity. Surprisingly, the other mRNA transcript found with near complete coverage was ND9 (one gRNA has a single nt mismatch). All of the other mitochondrially encoded Complex I members did have substantial gaps in coverage. Currently, there is considerable debate on the necessity of Complex I subunits for either stage of the trypanosome life cycle. Studies using RNAi and knockout cell lines of nuclear-encoded members of Complex I have shown that the complex is unnecessary for survival in either life cycle stage [51,52]. However, the nuclear-encoded Complex I member genes are maintained [29], and while we not did identify full coverage for the ND transcripts, a vast majority of the gRNAs were found in both life cycle stages.

This study used high-throughput sequencing to characterize the gRNA transcriptome during the bloodstream stage of the trypanosome life cycle. This work suggests that gRNAs are expressed during both life cycle stages, and that differential editing patterns observed for the different mitochondrial mRNA transcripts are not due to the presence or absence of gRNAs.

### Accession numbers

SAMN04302078, SAMN04302079, SAMN04302080, and SAMN04302081 NCBI’s Sequence Read Archive.

## Supporting Information

S1 Fig Quantification of the number of identified bloodstream and procyclic gRNA transcripts that cover a respective nucleotide in the fully edited mRNA. Bloodstream gRNAs are shown in dark gray and procyclic gRNAs are shown in light gray. Nucleotides and deletion sites were both numbered as edited positions in the mRNA transcripts starting from the 5’ end (+1 = 0). Boxes indicate the positions of identified populations of gRNAs (coverage ranges shown in parenthesis). Boxes with dark gray or light gray diagonal stripes indicate populations identified only in the bloodstream or procyclic transcriptomes respectively. A. ATPase subunit 6; B. Cytochrome oxidase III; C. C-rich region 3; D. C-rich region 4; E. NADH dehydrogenase subunit 3; F. NADH dehydrogenase subunit 7; G. NADH dehydrogenase subunit 8; H. NADH dehydrogenase subunit 9; I. Ribosomal Protein S12. All individual data points were designated with solid circles. Close overlapping of individual data points generate the observed solid lines.
(DOCX)

S2 Fig Alignment of the mitochondrial fully edited mRNAs and the most abundant gRNAs required for full coverage identified in the bloodstream (blue) and procyclic (gray) life cycle stages. Conservative mutations between gRNAs are shown in green and mutations that disrupt alignment are shown in red. Lowercase u’s indicate uridylates added by editing, asterisks indicate encoded uridylates deleted during editing. Nucleotides and deletion sites in the fully edited mRNA were numbered starting from the 5’ end (+1 = 0). Watson-Crick (|) and G:U (:) base pairs are indicated. Mismatches are indicated by the number sign (#). A) ATPase 6; B) Cytochrome Oxidase III; C) C-Rich Region 3; D) C-Rich Region 4; E) Cytochrome b; F) Maxicircle Unidentified Reading Frame II (Murf II); G) NADH Dehydrogenase Subunit 3; H) NADH Dehydrogenase Subunit 7; I) NADH Dehydrogenase Subunit 8; J) NADH Dehydrogenase Subunit 9; K) Ribosomal Protein S12.
(DOCX)

S1 Table All gRNA major classes identified in the EATRO 164 procyclic (shaded gray) and bloodstream (white) transcriptomes. Populations of gRNAs are bordered boxes. A) ATPase 6; B) Cytochrome Oxidase III; C) C-Rich Region 3; D) C- Rich Region 4; E) Cytochrome b; F) Maxicircle Unidentified Reading Frame II (Murf II); G) NADH Dehydrogenase Subunit 3; H) NADH Dehydrogenase Subunit 7; I) NADH Dehydrogenase Subunit 8; J) NADH Dehydrogenase Subunit 9; K) Ribosomal Protein S12.
(DOCX)
