# Supplementary material for: Analysis of the Trypanosoma brucei EATRO 164 Bloodstream Guide RNA Transcriptome
Source: PLoS Negl Trop Dis. 2016 Jul 11;10(7):e0004793. doi: 10.1371/journal.pntd.0004793 (PMC4939953; doi:10.1371/journal.pntd.0004793)

Supplementary Figure 1: Quantification of the number of identified bloodstream and procyclic gRNA transcripts that cover a respective nucleotide in the fully edited mRNA. Bloodstream gRNAs are shown in dark gray and procyclic gRNAs are shown in light gray. Nucleotides and deletion sites were both numbered as edited positions in the mRNA transcripts starting from the 5’ end (+1 =0). Boxes indicate the positions of identified populations of gRNAs (coverage ranges shown in parenthesis). Boxes with dark gray or light gray diagonal stripes indicate populations identified only in the bloodstream or procyclic transcriptomes respectively. A. ATPase subunit 6; B. Cytochrome oxidase III; C. C-rich region 3; D. C-rich region 4; E. NADH dehydrogenase subunit 3; F. NADH dehydrogenase subunit 7; G. NADH dehydrogenase subunit 8; H. NADH dehydrogenase subunit 9; I. Ribosomal Protein S12. All individual data points were designated with solid circles. Close overlapping of individual data points generate the observed solid lines.

A. ATPase subunit 6


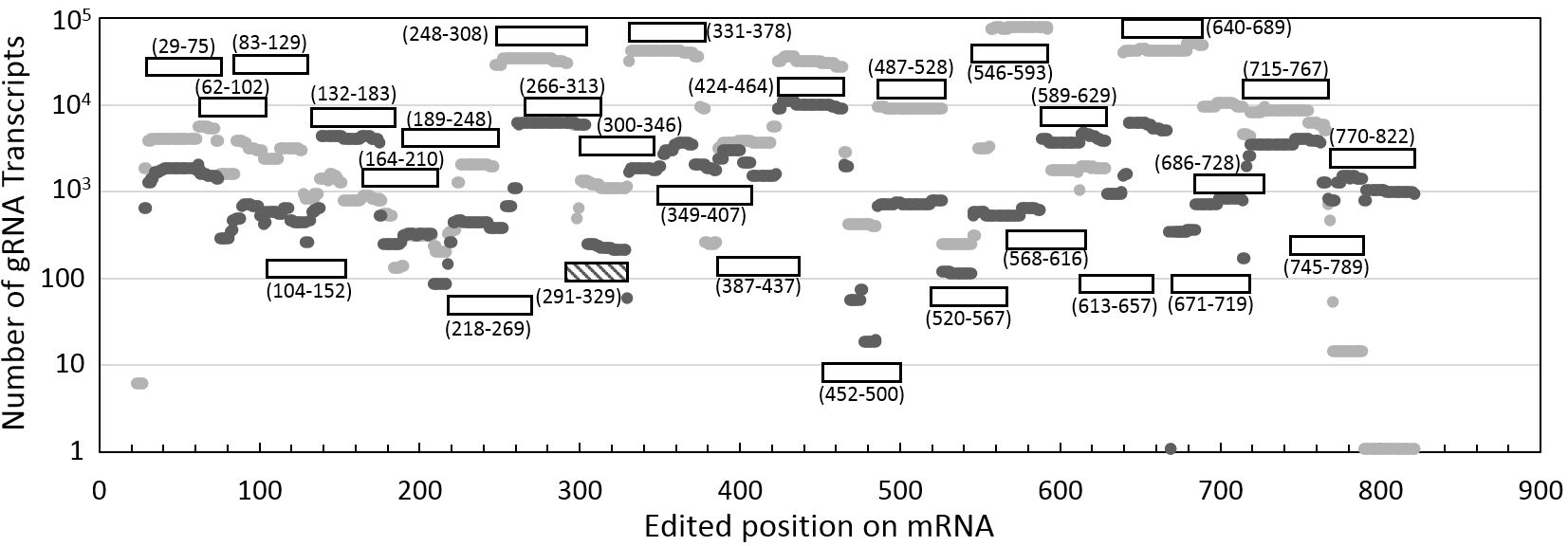


B. Cytochrome oxidase III


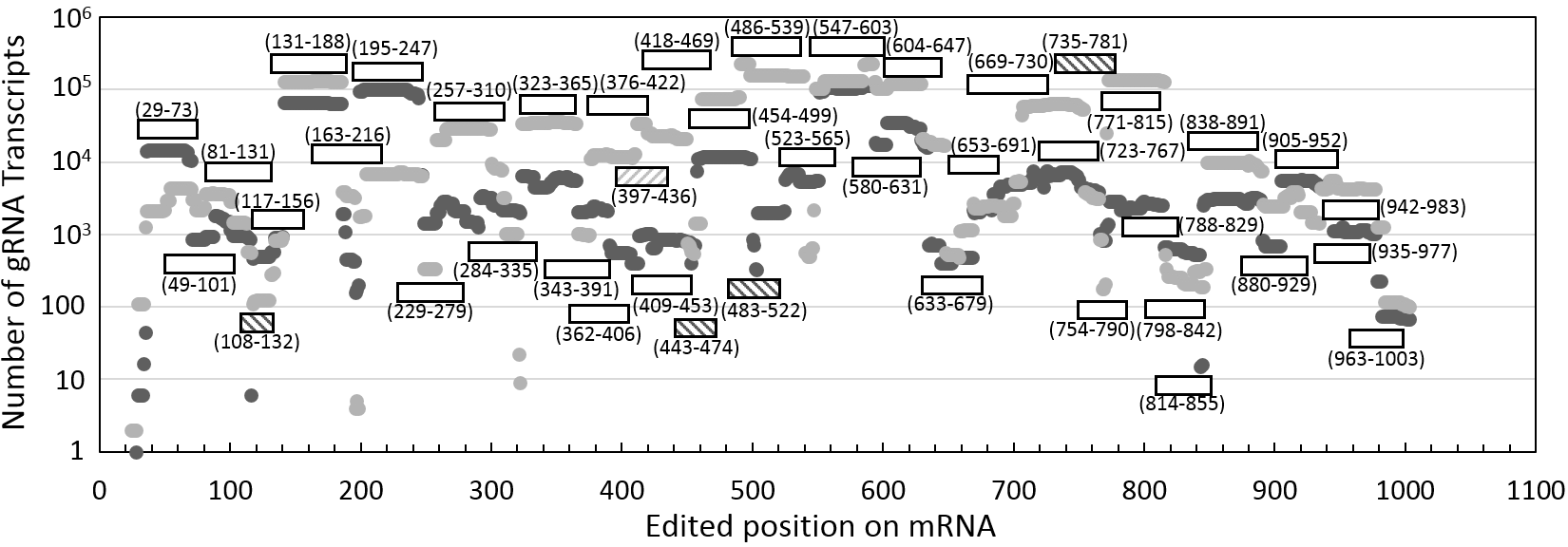


C. C-rich region 3


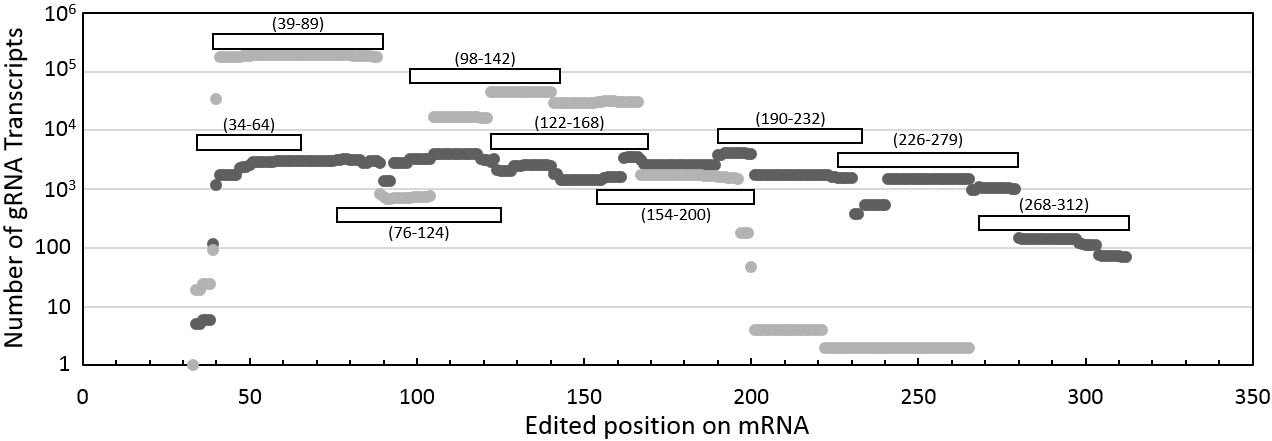


D. C-rich region 4


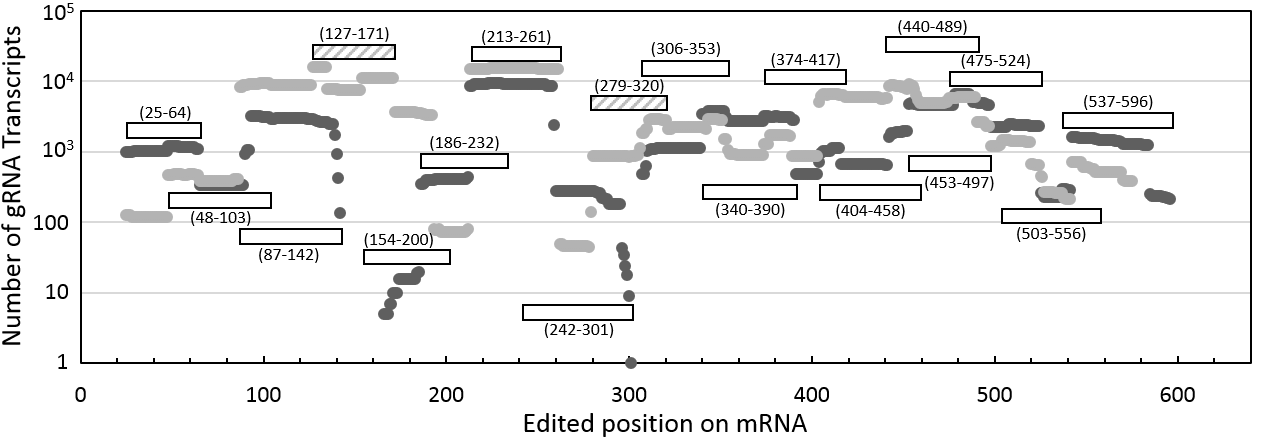


E. NADH dehydrogenase subunit 3


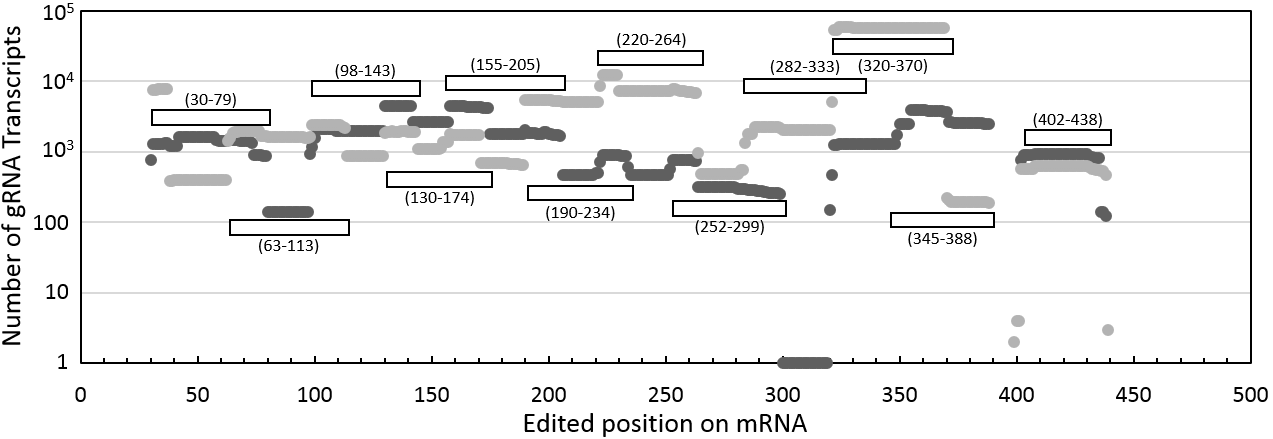


F. NADH dehydrogenase subunit 7


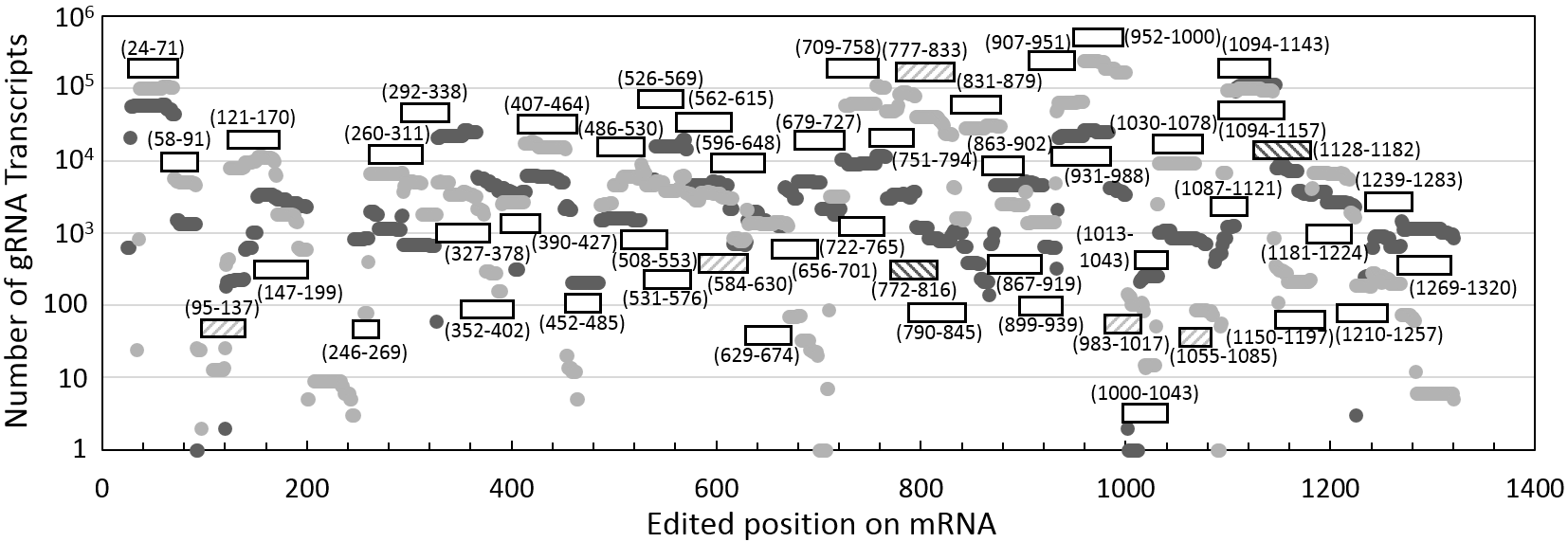


G. NADH dehydrogenase subunit 8


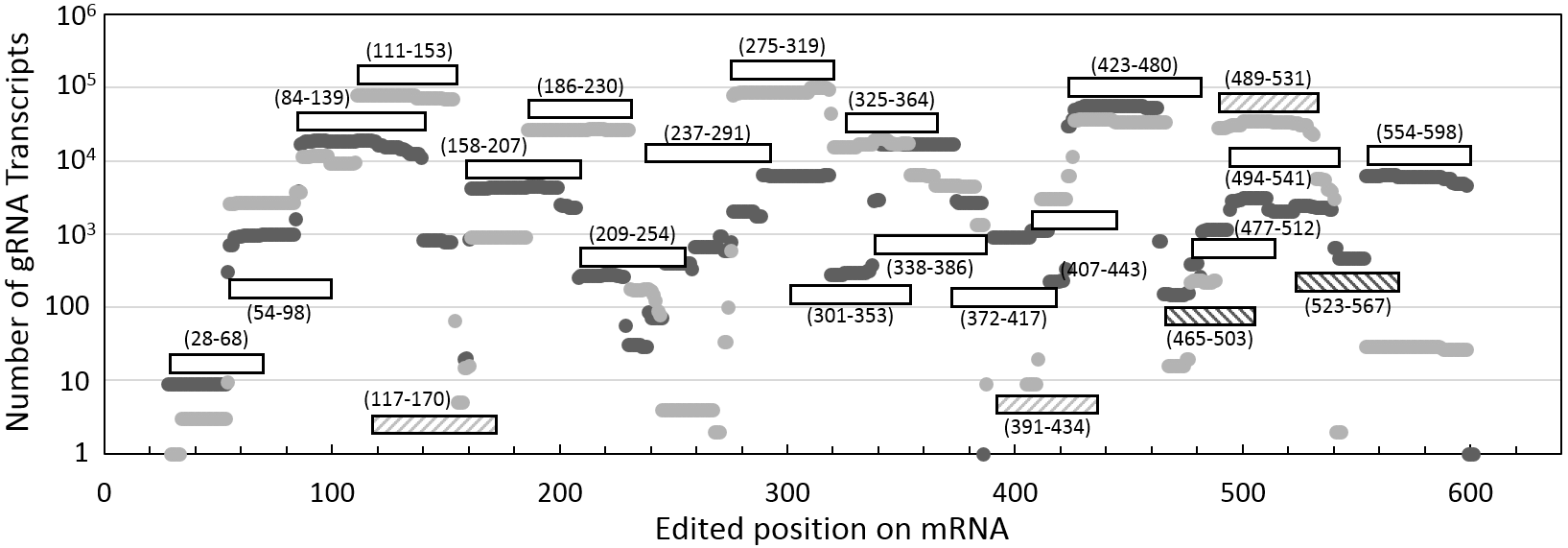


H. NADH dehydrogenase subunit 9


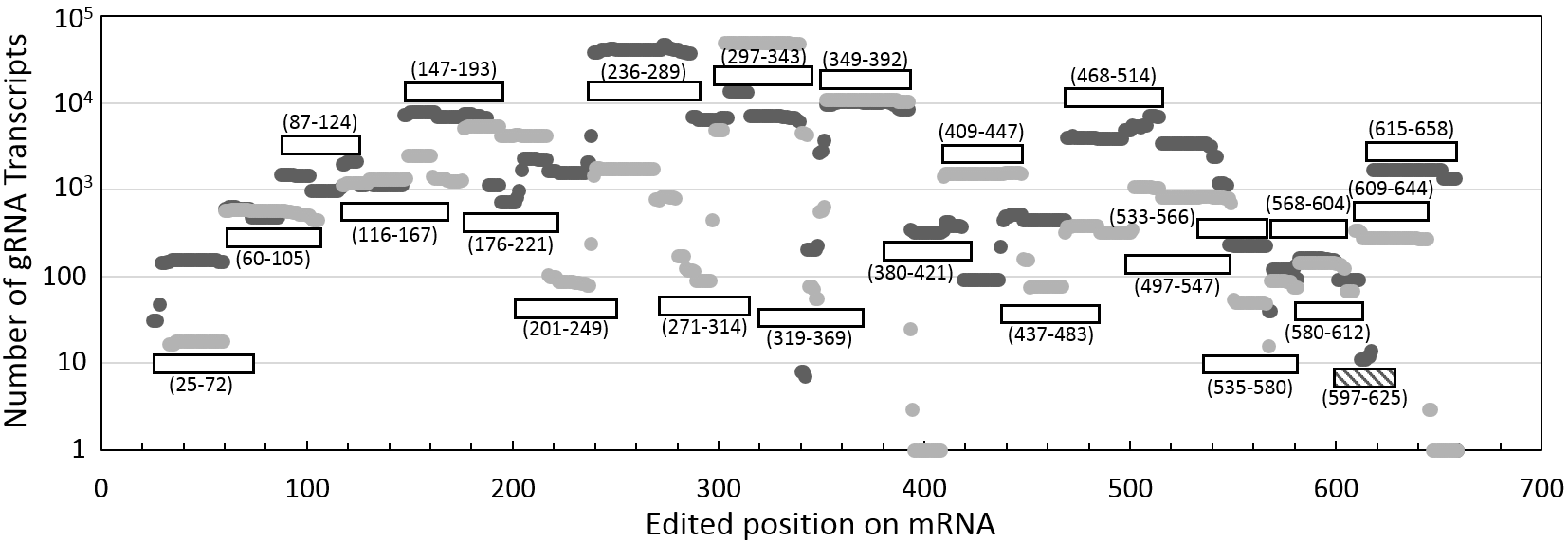


I. Ribosomal Protein S12


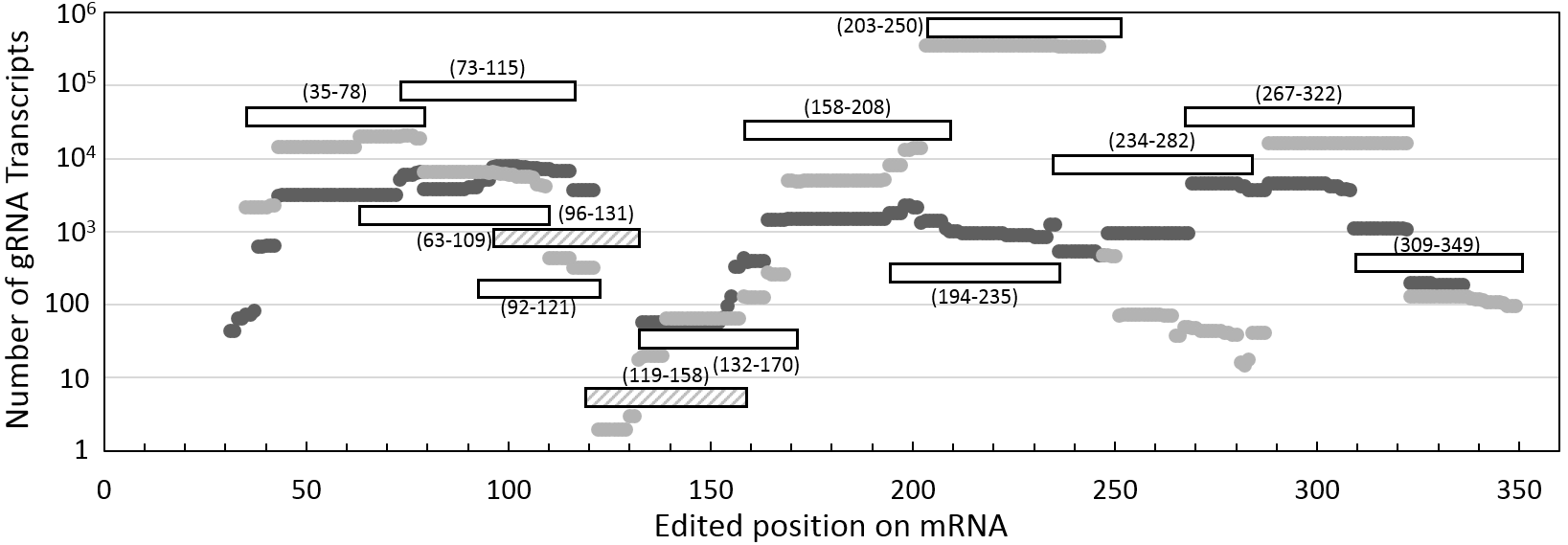

Supplement: S1 Fig — Bloodstream gRNAs are shown in dark gray and procyclic gRNAs are shown in light gray. Nucleotides and deletion sites were both numbered as edited positions in the mRNA transcripts starting from the 5’ end (+1 = 0). Boxes indicate the positions of identified populations of gRNAs (coverage ranges shown in parenthesis). Boxes with dark gray or light gray diagonal stripes indicate populations identified only in the bloodstream or procyclic transcriptomes respectively. A. ATPase subunit 6; B. Cytochrome oxidase III; C. C-rich region 3; D. C-rich region 4; E. NADH dehydrogenase subunit 3; F. NADH dehydrogenase subunit 7; G. NADH dehydrogenase subunit 8; H. NADH dehydrogenase subunit 9; I. Ribosomal Protein S12. All individual data points were designated with solid circles. Close overlapping of individual data points generate the observed solid lines. (DOCX) [file pntd.0004793.s001.docx]
