# Supplementary material for: Analysis of the Trypanosoma brucei EATRO 164 Bloodstream Guide RNA Transcriptome
Source: PLoS Negl Trop Dis. 2016 Jul 11;10(7):e0004793. doi: 10.1371/journal.pntd.0004793 (PMC4939953; doi:10.1371/journal.pntd.0004793)
Supplement: S2 Fig — Conservative mutations between gRNAs are shown in green and mutations that disrupt alignment are shown in red. Lowercase u’s indicate uridylates added by editing, asterisks indicate encoded uridylates deleted during editing. Nucleotides and deletion sites in the fully edited mRNA were numbered starting from the 5’ end (+1 = 0). Watson-Crick (|) and G:U (:) base pairs are indicated. Mismatches are indicated by the number sign (#). A) ATPase 6; B) Cytochrome Oxidase III; C) C-Rich Region 3; D) C-Rich Region 4; E) Cytochrome b; F) Maxicircle Unidentified Reading Frame II (Murf II); G) NADH Dehydrogenase Subunit 3; H) NADH Dehydrogenase Subunit 7; I) NADH Dehydrogenase Subunit 8; J) NADH Dehydrogenase Subunit 9; K) Ribosomal Protein S12. (DOCX) [file pntd.0004793.s002.docx]

**Supplementary Figure 2**. Alignment of the mitochondrial fully edited mRNAs and the most abundant gRNAs required for full coverage identified in the bloodstream (blue) and procyclic (gray) life cycle stages. Conservative mutations between gRNAs are shown in green and mutations that disrupt alignment are shown in red. Lowercase u’s indicate uridylates added by editing, asterisks indicate encoded uridylates deleted during editing. Nucleotides and deletion sites in the fully edited mRNA were numbered starting from the 5’ end (+1=0). Watson-Crick (|) and G:U (:) base pairs are indicated. Mismatches are indicated by the number sign (#). A) ATPase 6; B) Cytochrome Oxidase III; C) C-Rich Region 3; D) C-Rich Region 4; E) Cytochrome b; F) Maxicircle Unidentified Reading Frame II (Murf II); G) NADH Dehydrogenase Subunit 3; H) NADH Dehydrogenase Subunit 7; I) NADH Dehydrogenase Subunit 8; J) NADH Dehydrogenase Subunit 9; K) Ribosomal Protein S12.

A) ATPase subunit 6

**0 10 20 30 40 50 60 70 80 90 AAAAAUAAGUAUUUUGAUAUUAUUAAAGUAAAuAuGuuuuuAuuuuuuuuuuGuGAuuuAUUUUGGuuGCGuuuGuuAuuAuGuAuGuAuuAuuGuGuAu**

**|||||||::||:|:|||:::||::|||||||:|:||||||||||**

**_11_TTTTATACGGAAGTGAAAGGGAAGTACTAAATGAGACCAACGCAACATATA 5’ pA6(29-72)**

**_11_TTTTGTATAGAAATGAAGAGAAGGTACTAGGTAAAACCAATGCAAATATA 5’ bsA6(29-75)**

**||::|::|||:|:|:|:|||:||||||:||:|||||||**

**pA6(62-102) _11_TAATTAGTGCAGATAGTGATATATACATGATGACACATA**

**bsA6(62-100) _22_TAATTAGTGTAGATAATGATACATATATAGTAACACATA**

**:|||:||::|::||**

**pA6(86-127) _10_TTATAGTAGTATGTA**

**bsA6(90-129) _10_TATAGTATATG**

**100 110 120 130 140 150 160 170 180 190**

**GAuCuAGGuuAuGuuuuAuuGuGuAuuuuAAuUGuuuAAuGuuGAuuuuuGAuuuuuuAuuAuuuuGuuuG*UUUGAuuuGuAuuuGuuuGuuGGuuuGu**

**||| :::|:|: |:|||:||:|||:|:||:||||:|||||**

**CTAACATA 5’ pA6(62-102) pA6(164-208) _12_TATTATGTGGTAGAT-AGACTGAATATAGATAAGCAACTAAACA**

**C-AAAATA 5’ bsA6(62-100) bsA6(164-208) _14_TATTAGTAGAT-AGACTGAATATAGACAGATAACCAAACA**

**:|:||||:|:||:||:||:||||||||| |::||:::**

**TTGGATCTAGTATAAGATGACACATAAATATA 5’ pA6(86-127) pA6(192-243) _04_TATTAAGTG**

**TTAGATTCAATACGAGATAGCACATAAAATATATA 5’ bsA6(90-129) bsA6(190-243) _19_TTAATTAAGTG**

**||:|||::::|||:||||||:|:|||:|||||||||||||**

**_12_TAATAGAAGATAGTGTATAGAATTGACAGATTGCAACTAAAAACTACATA 5’ pA6(113-152)**

**_12_TTTTAATATAGAATGGTGCATGAAATTGACGAGTTACAACTAAAA-CTATA 5’ bsA6(105-148)**

**||:|::||:||::|::||:|||||:|:|||:|| ||||||||||||**

**_11_TGATATAGTTAGAAGTTGGAAGATAATGAGACAGAC-AAACTAAACATATA 5’(138-183)**

**_10_TAATAAGTGGTAGTTAGAGACTGGAAAATAGTAAAACAAAC-AAAT-AAATA 5’ bsA6(139-175)**

**200 210 220 230 240 250 260 270 280 290**

**G***UUUGuuuuuAuuGuuGuGGuuuAuGuuGuuuAAuuuAuAuAGuuuAAUUUUGuAuuA*UUGuAuuACuUAUUUG***AAuuuG*UAuuUGuuGuuu**

**| |||:| ||||||:||::: ||:||: ||::|||::|:|**

**C---AAATATATA pA6(164-208) pA6(266-313) _12_TTAATGAGTAGGT---TTGAAT-ATGGACAGTAGA**

**C---GAATATA 5’ bsA6(164-208) bsA6(281-313) _09_TAGTGTATAGA----TTAGAT-ATGGATGATAAG**

**: |||::||:|||:::|||::|:||||||||||||||||||| bsA6(291-329) _16_TAATAGTAGA**

**T---AAATGAAGATAGTGACATTAGATACAACAAATTAAATATA 5’ pA6(192-243) pA6(301-345) _11_TAAA**

**T---AAATGGAAATAGTAGTATCAAGTACAACAAATTAAATATA 5’ bsA6(190-243) bsA6(301-345) _15_TAAA**

**|:||:|::|||||||:|:|||||:||||:|:|||||| ||||||||**

**_11_TAGTATAGTAAATTAAGTGTATCAGATTAGAGCATAAT-AACATAATAATACA 5’ pA6(224-269)**

**_02_TTTAAGTGTAACAGATTGAATATGTCAAGTTAAAACATAAT-A-TATATA 5’ bsA6(221-262)**

**||||:|::|||:| |||||:|||:||:|:| |||||| |||||**

**pA6(248-292) _13_TATTAGAGTATAGT-AACATGATGGATGAGC---TTAAAC-ATAAAATATA5’**

**bsA6(254-298) _17_TATATAGT-AGTATGATGGATAGAC---TTAAGC-ATAAACAACAATATATA**

**300 310 320 330 340 350 360 370 380 390**

**uGuAuuGuuuuuuuAuuGuAuAuuGCAuuuuuAuuuuuGuuuuGuuuuuuAuGuGAuuuuuuuuuGuuuAAuAAuuuGuUAGuuGGuGAuA****Guuuu**

**|||||||||||||| ||||::|||||:|||:|:|||::||:||||| :||||**

**ACATAACAAAAAAAAAAAAA 5’ pA6(266-313) pA6(360-407) _13_TAAAAGTAAATTGTTAGATAATTGACTACTAT----TAAAA**

**ACATAACAAAAAAAAAA 5’ bsA6(281-313) bsA6(352-401) _12_TACTAAGAGAAGATGAATTGTTAGGTAATCAATCACTAT----CAAAA**

**ATATAGTAAAGAGATAGCATATAACGTAAATATATAAA 5’ bsA6(291-329) :||| :|:|:**

**::||::|||||:|||::||||:||||:||:||:|||||||||||| pA6(387-435) _10_TTTAT----TAGAG**

**CTGTAGTAAAAAGATAGTATATGACGTGAAGATGAAAACAAAACAATATA 5’ pA6(301-345) bsA6(387-435) _11_TTTAT----TAAAG**

**CTGTAGTAAAAAGATAGTATGTGACGTGAAAATGAAAACAAAACAATATA 5’ bsA6(301-345)**

**|||:||::||:|:::|||||:||||:|||:|:|||||||||||||**

**pA6(331-375) _12_TATAGAAGTAAGATGGAAAATGCACTGAAAGAGAACAAATTATTAATATA 5’**

**bsA6(331-371) _15_TATAGAGATAAGACAGAAGATGCACTAAAGAAAAACAAATTAAATATA 5’**

**400 410 420 430 440 450 460 470 480 490**

**AuGGAuGuuuuuuuuAUUC**GuuuuuuGuuGuGuuuuuuAGAGuGuuuuuCuuuGuuGuGuCGuuGuuuGuCGACGuuuuuGCGuuuGUUUUGuAAuuu**

**|||||||| :||:|:||::::||::|||||:|:|||:|||:|||||||||||**

**TACCTACATATATA 5’ pA6(360-407) pA6(455-497) _14_TTAATATGGTGGTAAGTAGCTGTAGAAATGCAGACAAAACATTATATA 5’**

**TAAT-ATA 5’ bsA6(352-401) _04_TAATTAGAGTAACATAGCAATAGATAGCTGCATTAA 5’ bsA6(452-477)**

**||||||::||:|:|:|||| ||||:|||||||||| |:|:|:::|||:|**

**TATCTATGAAGAGAGTAAG--CAAAGAACAACACAATATATA 5’ pA6(387-435) pA6(487-526) _16_TATAGAGTGTTAGA**

**TGTCTATAGAAAAGATAAG--CAGAGAACAACACAATATATA 5’ bsA6(387-435) bsA6(487-526) _16_TATAGAGTGTTAGA**

**|||:::|||||::||||||||:|:||:|:|:||||||||:|**

**_12_TAAAGTGACACAGGAAATCTCATAGAAGGGAGCAACACAGTATATA 5’ pA6(424-464)**

**_13_TAAAGCGACATGGAAAATCTCATAGAAGGGAGCAACACAGTATATA 5’ bsA6(424-464)**

**bsA6(458-500) _15_TATAGTAATAGATAGCTGCGAAGATGCAAACAGAACATTAAA**

**500 510 520 530 540 550 560 570 580 590**

**AuuAuCAuCCCAuUUUUUAuuGuuGAuGuuuuuuGAuuuuuuuUAuuuuAuuuuuGuuuuuuuuuuuuAuGGuGuuuuuuGuuAuuGAuuuAuuuuAuuu**

**|||||||||||||:||:|||||||||| ||::|:|:||:|:|:|:|||:||||||||||:**

**TAATAGTAGGGTAGAAGATAACAACTAAACATA 5’(487-526) pA6(568-611) _12_TTATTATAGAAGATAGTGACTGAATAAAATAAG**

**TAATAGTAGGGTAGAAGATAACAACTAAACATA 5’ bsA6(487-526) bsA6(576-616) _07_TACATATAGAATAGTGACTGGATGAAATGAA**

**:||||::||:|:|||:|||:||||||:|||||||||||||||||||| |:|:||||:||**

**pA6(521-567) _05_TTAATTGTAAGAGACTGAAAGAAATAAGATAAAAACAAAAAAAAAAAAAA 5’ (589-629)_13_AATTTAGTGAAATGAA**

**bsA6(520-553) _04_TATATAACTGTGAGAGACTAAGAAGAATGAAATAAAA-CAAAAAAAAAAA 5’ (589-629)_10_AATTTAGTGAAATGAA**

**||||:|:|::#||:|:|:|||||::|||||:||||||||:||||||||**

**TTTAAA 5’ bsA6(458-500) pA6(557-593) _09_TATAAATGAGAGT-AAGAGAGAAAATGTCACAAGAAACAATAGCTAAATAA 5’**

**bsA6(546-592) _11_TAAATGAGAGTGAAAGAGAGAGATACCGTAGAAGACAATAACTAAATATA 5’**

**600 610 620 630 640 650 660 670 680 690**

**AuuuuuGuGuuuuGuuuuuGuuuAuuAuuuuAUGuGuuuuuAuAuUUGuuGGAuuuAUUuGCC***GCCAuAuuAC****AGuuAuuuAuuuuuuGuAAu**

**|||||||||||| |:|:|:||||::|:|:||||**

**TAAAAACACAAATCATA 5’ pA6(568-611) pA6(680-714) _13_TAAT-----TTAGTGAATAGGAGATATTG**

**TAAAAACACAAAACAAATA 5’(576-616) bsA6(671-714) _14_TTAATG----TTAGTAAGTGGAGAATATTA**

**|||:|:||:||::||||:|||||||||||| |:**

**TAAGAGCATAAGGCAAAGACAAATAATAAATA 5’ pA6(589-629) pA6(698-728) _11_TAATAAGAGATAGTG**

**TAAGAGCATAAGGCAAAGACAAATAATAAATA 5’ bsA6(589-629) bsA6(699-727) _11_TAATAAGAAATATGA**

**:||||:::|||:|||||||::||:|:||||||:|||||||||**

**_12_TTAAAAGTGAATGATAAAATGTACGAGAATATAGACAACCTAATATATA5’** **pA6(613-654)**

**_11_TTTAAAAGTGAATGATAAAATGTACGAGAATATAGACAACCTAATATA 5’ bsA6(613-654)**

**|||:|:|:::|:|:||:|:|| :|||||:||| ||||||||||**

**pA6(640-689) _12_TTATATAGATAGTTTGAGTAGATGG---TGGTATGATG----TCAATAAATATATA 5’**

**bsA6(643-667)_15_TAAGTAGTCTAGGTAGATGG---CGTTATAGTG----TCAATAAATATATACA 5’**

**700 710 720 730 740 750 760 770 780 790**

**AuGAuuuuGCAGuuGAuAAuGG**AuuuuuuGuuGuuuuuGuuGuuuGuuuAGuuuuGuAuuuGAuuuuuGAuAGuuAuuAuAuuGuuGuuGAAAuuuG***

**|:|||:||||||||| :|||:||||:||||:::|||::||||:||**

**TGCTAGAACGTCAACATAAAA 5’ pA6(680-714) pA6(770-822)TCTCTTCTTTCCCTTTATTAATAGTATAGTGACAGTTTTAGAC-**

**TACTAGAACGTCAACATAGA 5’ bsA6(671-714) bsA6(773-822) _09_TTTAATAGTATAGTGACAGTTTTAGAC-**

**||:|:|:|:||:|:|||||||| |||||**

**TATTGAGATGTTAGCTATTACC--TAAAATTA pA6(698-728)**

**TGTTAGAATGTCAATTATTACC--TAAATATATA 5’ bsA6(699-727)**

**:: ||||:|:::|:|:|||:|:||:|:||:||||:||||||||||||**

**_11_TTT--TAAAGAGTGATAGAAATAGCAGATAAGTCAAGACATAAACTAAATA 5’ pA6(720-767)**

**_14_TTTATT--TAGAGAGTAGCAAAGACAGTAAGTAGATCAAAACATAAAT-ATATA 5’ bsA6(717-763)**

**:||||:|:|::||||::|::||:||:|||||||||||||||||**

**pA6(747-789) _11_TTAAATTAGAGTATAAGTTGGAAGCTGTCAATAATATAACAACATAAAA 5’**

**bsA6(747-789) _11_TTAAATTAGAGTGTAAGTTGGAGACTATCGATAATATAACAACATATATA 5’**

**800 810 820 830 840**

***GuuUGuuA**UUGGAGUUAUAGAAUAAGAUCAAAUAAGUUAAUAAUA_**

**:||:|||| |:||||||||||**

**-TAAGCAAT--AGCCTCAATATCAGG 5’**

**-TAAGCAAT--AGCCTCAATATCATATA 5’**

**Alternate initiating gRNA (procyclic transcriptome only)**

**750 760 770 780 790 800 810 820 830**

**uAGuuuuGuAuuuGAuuuuuGAuAGuuAuuAuAuuGuuG*uGAAA*uuG**GuuuUGuuA**UUGGAGUUAUAGAAUAAGAUCAAAU**

**:||||:|||::::|| |:||| ||| :|||:||:| ||||||||||||**

**pA6(774-822) *_14_TAATAGTATGGTGAC-ATTTT-GAC--TAAAGCAGT--AACCTCAATATCATA 5’**

B) Cytochrome Oxidase III

**0 10 20 30 40 50 60 70 80 90**

**GGUUAUUGAGGAUUGUUUAAAAUUGAAUAAuuAuuAuuuuuuuAuGuuuuuGuuuC*****GuuGuAuAuuuGuuGGuGuuA****GuGGuGuuuuuGuu**

**|||:||||||||:|:|:|:|| ||||||||||**

**pCO3(35-70)_11_TATG-TAGTTAAGAAAATGCAGAGATAGAG-----CAACATATAATTAATA 5’**

**bsCO3(36-70)_12_TATATATGGTAGAAAAGAGATACAAGAATAGAG-----CAACATATAATATATA 5’**

**|| :|::|||||::||:::|:|:| |||:||||||||||**

**pCO3(54-101) _11_TAAATAG-----TAGTATATAGGCAGTTATAGT----CACTACAAAAACAA**

**bsCO3(51-99) _09_TAAAG-----TAGTATATAAACAGTTACGAT----CATCACAAAAACAA**

**| :||||:|:||:::|**

**pCO3(81-116) _10_TT----TACTATAGAAGTGA**

**bsCO3(88-115) _07_TCTATAGAAGTAA**

**100 110 120 130 140 150 160 170 180 190**

**uuuuuAuCuuuACCuGCCAuuGuuAuuGuGuAuuGGuuAuuuuGuuuGuuG****GGAuuuAuuuGuuuAuuGUUUG****GuAGuuuuuuAuuuGuuGA**

**|| |::|:|:||: |:||:||:|:||:||||||||| |||:|**

**AATATA 5’ pCO3(141-185) _12_TAATTTAGTAGATAAT----CTTAGATGAGCAGATAACAAAC----CATTATATA 5’**

**TATA 5’ bsCO3(141-185) _14_TAATTTAGTAGATAAT----CTTAGATGAGCAGATAACAAAC----CATTATATA 5’**

**||:|:|:||||||||||#|||||||||:|:||| |::::|||::|:|: |:||||:|:||||||||||**

**AAGAGTGGAAATGGACGATAACAATAATATATA 5’(81-116) (163-203) _10_TAGATATAGTGGATAGTAGAT----CGTCAAGAGATAAACAACT**

**AGAGATAGAGATGGATAGGTAGCAATAA 5’(88-115) bsCO3(168-195) _12_TATAGTGAAT----TATCAGAGAATAGACTACT**

**_09_TAGATGGATGGTGACAATAACATATATA 5’bsCO3(108-132)**

**||:|:|:|:|:|||||:|||||:|:||||||||| |: :||:|**

**(117-156) _13_TATATAGTGATAGTGATACATAGCCAATGAGACAAACAAC----CTATATATA 5’ pCO3(195-247) _09_TAATT**

**(117-155)_16_TGATAGTAGTAGTGACACGTGATCAATAAGACAAACAAC----C-ATATACA 5’ bsCO3(195-244) _13_TAATT**

**200 210 220 230 240 250 260 270 280 290**

**uuGuG****GuuuuAuuuuuuuuuuuGuuGGuuuuuGuAuuuGuuuGuuGuuGuuAuuGuuAGAuuuGuuuuGuGAuuuuuuACGuGGuuuAuuuGAuuu**

**||:| |:|||:||:|:|:|:||:|:|||:|||:|:|||||||||||||**

**AATAA----AA 5’(163-203) pCO3(258-299) _10_TATAATTTAGATAGAGCATTGAAAGATGTATCAAATAAACTAAA**

**AACAC----CAAAATATATA 5’(168-195) bsCO3(259-300) _13_TTAATTTAGATAAGATATTGAGAAGTGCACTAGATAAACTAAA**

**|:::: :|:|||:|:|:|||:|||:||:|||||||||||:|||:|**

**AGTGT----TAGAATGAGAGAAAGAACGACTAAAAACATAAATAAATA 5’(195-247)**

**AGTGT----CAAGATGGAAAAAGAAGTAGCCGAGAACATAAACAATATA 5’(195-244)**

**::|:|:|:|||:|:|:||||::|:|:|:|||||||||||||||||: ||:||:|**

**pCO3(229-274) _10_TTTAGAGATATAGATAGACAATGATAGTGACAATCTAAACAAAACATATA 5’(293-320) _10_TAATTAGA**

**bsCO3(236-279) _11_TTATAAATGAATAATGACAATGATAGTCTAGACAAGACACTAAAATATA 5 _11_TAAATTGAA**

**300 310 320 330 340 350 360 370 380 390**

**uuGuGuuuuAuuACGuuGuAuCCAGuAuuGuuuuuuAuGGuuuuuAuGuAG*UGAGuuuGuuuuAuuuAuGGCGuuuuuuG**UUGuAuuAuuuGGuuuA**

**|:|:| ||:||: :||:||:::||||:|||||:||:|::||| |||||||||**

**TATATA 5’(258-299) pCO3(345-391) _12_TATATT-GCTTAAGTGAAATGAATACTGCGAGGAAC--AACATAATATATATA 5’**

**ATATATA 5’(259-300) bsCO3(345-389) _03_TATATT-GCTTAAATGAGATAAATGCTGCAGAGAAC--AACATAAAATATATA 5’**

**:|:|:|:|||:|||:|::|||#||||||| ||||:|||::|::|:||:: |||||:|||:||:||||**

**GATATAGAATGATGTAGTATATGTCATAA 5’(293-320) pCO3(362-406) _09_TAATAGATATTGTGAGAAGT--AACATGATAGACTAAAT**

**AGTGTAAGATAATGTAGCATGGGTCATAACAAATATATA 5’bsCO3(291-332) _06_TAATAGATACTGTGAGAAGT--GACATAATAGACTAAAT**

**|||:: :||:||:|||::||:||**

**pCO3(376-418) _10_TTTAAAGT--GACGTAGTAAGTCAGAT**

**bsCO3(378-422) _17_TAATTAAT--AGTGTGATAGACTAGAT**

**|:|||:::||||||::|||:|||::||| :||||||||||||| :||**

**pCO3(323-365) _11_TATTATAGTGAAAAATGTCAAGAATGTATC-GCTCAAACAAAATATATA 5’ pCO3(397-436) _10_TGAT**

**bsCO3(323-365) _09_TATTATAGTGAAAGATGTTAGAAATGTGTC-ACTCAAACAAAATATATATA 5’**

**400 410 420 430 440 450 460 470 480 490**

**uGuuuAuuuuuGuGuuGuGAGuuuGCUUUCGuuuuuuGuuuACCuuAuAuGuuuuGuuGuuuAuuAuGuGAuuAuGGuuuuGuuuuuuAuuGG*UAuuuu**

**||||||| |||:|::|||:||:::||:|||:||:|||||| ||||**

**ACAAATATATATA 5’(362-406) pCO3(461-497) _14_TACTTATAGTGTACTGATGTTAAGACAGAAGATAACC-ATAATACATA**

**ACAAATATATA bsCO3(362-406) bsCO3(457-499) _14_TATAAGTAGTATATTAGTGTCAGAGTAAAAGATAACC-ATAAAATTATA 5’**

**|||:||||||||||||:|: bsCO3(483-522) _14_TAAAAGTGACT-ATGGAA**

**ACAGATAAAAACACAATATACA 5’(376-418) :: ||||:|**

**ACGAATAAAGACACAACATTCAATATA 5’(378-422) pCO3(491-539) _10_TTT-ATAAGA**

**|:|:|||:|:|||:::||||:|:||||||||||||| bsCO3(504-535)_15_TTAGAA**

**ATAGATAGAGACATGGCACTTAGACGAAAGCAAAAAA AAAA 5’ (397-436)**

**||:|::::|||:|:|::||||:|:|::||||||||||||**

**_12_ATCATAGTGTTCAGATGGGAGCAGAGAGTAAATGGAATATAATATATA 5’pCO3(411-449)**

**_16_TTCAGTGTTTAAGCGAAGGTAGAAGATAAATGGAATATACAATATATA bsCO3(413-452)**

**|:|||||:|||::||:||:|:|||||||:|::||||||||||||||||:**

**_04_TATATTAAATGGAAGTGAAGAATAGATGGAATGTGTAAAACAACAAATAATATCATA 5’pCO3(418-467)**

**_10_TTTAAATGTAGGTAAAAAGTGAATGGAATATGCAAAACAACAA-TATTATA 5’ bsCO3(427-460)**

**_10_TAAAAGTAGATAGAATATGCAAGATAGTAAATAATACACTAATAA 5’bsCO3(443-474)**

**500 510 520 530 540 550 560 570 580 590**

**uuAGAuuuAuuuAAuuuGuuGAuAAAuACAuuuuAUUUGuuUGuuAGuGGuuuAuuuGuuAAuuuuuuuGuuuuGuGUUUUUGGuuuAGGuuuuuuuGuu**

**AATCTAAGTAAATTAAACAACTAATAAA 5’(483-522) |||::|:|:||::||**

**:||:|||:||:||||:::||||:|||||||||||||||:| pCO3(585-629) _12_TAATTTAGAGAAGTAA**

**GATTTAAGTAGATTAGGTAACTGTTTATGTAAAATAAATATA 5’(491-539) bsCO3(585-628) _13_TAATTTAGAGAAGCAG**

**GATGTAGGTAAATTGAGCAGCTGTTTATGTAAAATATATA 5’(504-535)**

**||||:|||:|:|:||||||||:||:|:|||||||||||**

**pCO3(528-565) _12_TATAGTAAGATAGATAGACAATCACTAAGTGAACAATTAAAATATATA 5’**

**bsCO3(525-563) _13_TAATATGTAGAGTAGATAAACAGTCACTAGATGAACAATTAATATATA 5’**

**::||||:|:::||||:|:|:||||:|:||||:|||:|||||||||**

**pCO3(548-592) _11_TTTAAATGAGTGATTAGAGAGACAAGATACAAGAACTAAATCCAAATATATA 5’**

**bsCO3(551-593) _13_TAATAGATGATTGAAGAGACGAGATACAAGAGCCAAATCCAAAATAACA 5’**

**600 610 620 630 640 650 660 670 680 690**

**G**UUGuuGuuuuGuAuuAuGAuuGAGuuuGuuGuuuG****GuuuuuuGuuuuuGuGAAACCAGuuAUGAGA**GUUUGCAuuGuuAuuuAuuACAuuA**

**| ||:|||:||::|||||||||||||||: :|:| :|||:||:||::||:||:|||||||**

**C--AATAACGAAGTATAATACTAACTCAAGATATA 5’(585-629) pCO3(669-717) _09_TTTTT--TAAATGTGACGGTAGATGATGTAAT**

**T--AATAGTAGAGCATAATACTAACTCAATATATA 5’(585-628) bsCO3(669-715) _08_TTTTT--TAAATGTAGTAGTAAATGATGTAGT**

**|:||:|:||:||:||::||::||||::|:||||| |||||| ||||||||:**

**_13_TATAATAGAATATGATGTTAGTTCAAGTAGCAAAC----CAAAAA#CAAAAACATA 5’ pCO3(604-647)**

**_13_TATAATAGAATATGATATTAGTTTGAACAGCGAGC----CATAAAACAAAAACATA 5’ bsCO3(604-643)**

**||: :|:|:|::|:|:|:|:||||||||:|||#:| |||||||||||**

**pCO3(635-669) _14_TAAT----TAGAGAGTAGAGATATTTTGGTCAGTACATT--CAAACGTAACATATA 5’**

**bsCO3(635-669) _11_TAAT----TAGAGAGTAGAGATATTTTGGTCAGTACATT--CAAACGTAACATATA 5’**

**|||||::|||||:| :||:|||||||||||||**

**pCO3(659-691) _12_TAACATAGATACTTGGTTGATACTTT--TAAGCGTAACAATAAATTATA 5’**

**bsCO3(653-682) _15_TAGATAGTAGTGATGTTTTGGTCAATATTCT--CAAGTGTATA 5’**

**700 710 720 730 740 750 760 770 780 790**

**AGuuGuGG****UGuuuuuGGuuCuAuuuuAuuuuuAuuGGAuuuAuUACAuuuuA**UGCAuGuuuuuuuAGGuGuuuuGuuGuuGuuuAuuuGuuuuA**

**||:||||| ||||:| :||:|:||:|:::|:|:||:|||:||:|**

**TCGACACC----ACAAGATAATA 5’(669-717) pCO3(772-815) _14_TATCATAGAATAGTGATAGATGAACGAAGT**

**TTAGTACC----ACAATA-CCAAGATATA 5’(669-715) bsCO3(773-814) _11_TATATAAAGTGACGGTAGATGAACAGAAT**

**:: |:||:|:::|||||::|:|:|||:||::|||:|||||||||| ||||**

**_10_TTT----ATAAGAGTTAAGATGGAGTGAAAGTAGTCTAGATAATGTAAATATATA 5’pCO3(706-753) pCO3(796-829) _06_TAAAT**

**_26_TGATATAT----ATAGAAGTTAAGATAGAATGAAGATGACTTAAATAATGTAAATATA 5’bsCO3(707-753) (788-829) _13_TAATAAGTGAAAT**

**||||:|||:|:|||:|:||||:|:|||||:||| |||||||| bsCO3(798-842) _14_TATAT**

**pCO3(723-765) _14_TGATAGAATGAGAATGATCTAAGTGATGTAGAAT--ACGTACAATATATA 5’**

**bsCO3(724-765) _10_TATAGAATAGAAGTGACTTAGATGATGTAGAAT--ACGTACAATATATA 5’**

**bsCO3(736-781) _17_TAATTTAGATAGTGTAAGAT--GCGTGTAGAAGAATCCACAAAACATATA 5’**

**|| |:|||::|:|:||||::|||:||||||||||||**

**pCO3(754-790) _15_TAATGTAGTAT--ATGTATGAGAGAATCTGCAAGACAACAACAAATTATATA 5’**

**bsCO3(754-790) _12_TAATGTAGTAT--ATGTATGAGAGAATCTACAAGACGACAACAAATTATATA 5’**

**800 810 820 830 840 850 860 870 880 890**

**uGCGuuuGuuuAAuuuuuuGuGuAuGGAuACACGuuuuGuuuuuuuGuAuuGuGuuuGuuuAuAuuGACAuuuuGuuGAUUUAGuuuGAuuuuuuuuAuu**

**|:|||:|||||||||| |||:|:|:|:|:||:||::||||:|:||::|||||||||||||**

**ATGCAGACAAATTAAATATA 5’(772-815)pCO3(848-890)_08_TATAATATAGATAGATGTAGTTGTAGAGCAGTTAAATCAAACTAATATATA 5’**

**ATGCAAACAAATTAA-TATA 5’(773-814)(845-889)_11_TATATATAGTGTAAGTAGATGTAGCTGTGAAATAACTAAATCAAATTATA 5’**

**|:|:|:|:|:|||||:|:|||:||||||||**

**ATGTAGATAGATTAAGAGACATATACCTATAGTGCAAAACAATA 5’ (796-829)**

**GTGCAGATAAATTAGAAGACACATACCTATATATA 5’(788-829)**

**|:|||::|||||:|:|::|:||||:|||||||||||||||| |||::||:|||:|:|:||||**

**TAGTAAATGAATTAGAGAGTATATACTTATGTGCAAAACAAAATATA 5’(802-842) pCO3(880-918) _13_TATAATTGAATTAAGAGAGATAA**

**ATGTAGGTAAATTGAGGGATATGTGTCTATGTGCAAAACAAAATA 5’(798-842) bsCO3(880-929) _14_TAATTGAATTGAAAGAGATAA**

**|||:::|:|||:|||||||||||:|:||:||||||||||:|**

**(814-854) _06_TAAAGGTATATATCTATGTGCAAAGCGAAGAAACATAACATATATA 5’**

**bsCO3(828-855) _22_TTATGTAGATGTGCAAAGTAGAGAGACATAACACAATATATA 5’**

**900 910 920 930 940 950 960 970 980 990**

**GCGAuuuGuuuAuuuuGAuGuuuuAuGuGuuAuGuAuuuGuGuGuGuAAuuuuAuuGGuGuuuuUUUAGUUGuuGAuuA*GuuAAuuuGuAuuGGUAGUU**

**|||||:||||||||:|||| |::|||:||:::|||:| |||||:||:|||||||||||**

**CGCTAGACAAATAAGACTAAATATA 5’(880-918) pCO3(963-1003) _08_TATATTGGAATTAATGGCTAGT-CAATTGAATATAACCATCAA**

**TGCTAAACAGATAAGACTACAAAATATATA 5’(880-929) bsCO3(965-1003) _17_TGTAATTAATAGTTAGT-CAATTGAATATAGCCATCAA**

**::||||:||:|::||:||:||:||||||||:|||||||:||**

**_13_TTGAATAGAATTGTAAGATGCATAATACATAGACACACATATAATATA 5’ pCO3(907-947)**

**_13_TATATAGTTAGAAGATACATGATACATAAGTACACACATTAAAAGATA 5’bsCO3(920-952)**

**||||:::|||::|||:|:||||:|:|:||||:|||||||||||**

**_12_TTAAATGTACATGTTAGAGTAACTATAGAAAAGTCAACAACTAAATATA 5’ pCO3(935-977)**

**bsCO3(940-977) _14_TAATTAGTGTATATTGAGATAGTCACAGAAGAATCAACAACTAAATATA 5’**

**::::||||:|:||||::|||:|:||||:||||||||| ||||**

**_14_TAATTAGTGTATTAGAGTAACTGCAAGAGAATCGACAACTAAT-CAATATA 5’ pCO3(942-983)**

**bsCO3(951-981) _10_TAATGTACA-TATAGTGACTATAAGAAGATCAACAACTAAT-CATA**

**1000**

**UGUAGGAAG**

**||||**

**ACATATA 5’**

**ACATATATA 5’**

C) C-rich region 3

**0 10 20 30 40 50 60 70 80 90**

**agaaauauaaauauguguaugauauauaaaaacaauguuuga****uuguuugguuuuguuguuuuuuuauuguuuguuuguacauuuuuuuuguuuuuu**

**|||:|:||| |||:|:|||||||||||**

**pCR3(33-62) _09_TAAAGTGAGATTATAGACT----AACGAGCCAAAACAACATGTATA 5’**

**bsCR3(34-62) _09_TAGTGTGAT-GTAAACT----AACAAGTCAAAACAACATATATA 5’**

**| |::|:|::|||:::||||:|:|:|||||::|||||||||||||**

**pCR3(41-88) _14_TAT----AGTAGATTAAAGTGACAAGAGAGTAACAGGCAAACATGTAAAA-TATATA 5’**

**bsCR3(41-89) _08_TTT----AGTAGATTAAAGTGACAAGGGAATAACGAGCAAACATGTAAAGATATA 5’**

**|::||||::|:|:|::||||:|**

**pCR3(78-118) _16_TATCATAGTATGTGGAGAGAGTAAAAGA**

**bsCR3(78-118) _04_TATCATAGTATGTGGAGAAAGTAAAAGA**

**pCR3(105-140) _13_TATAGTTAT**

**bsCR3(105-140) _12_TTAGTTAT**

**100 110 120 130 140 150 160 170 180 190**

**auuuguuugug***a**uuuguuuuuauguuuguua*uuuaguuuuuguuuuuuauuggauuuuuguuuuuuauuuaauauggguuuauuguuguguuua**

**||||||||||| | || |||::||:||:||:|:|:|:||||||:|:||||||||||||:|**

**TAAACAAACAC---T--AATATATA 5’(78-118) pCR3(154-196) _13_TTAAtttagaagtagagagtgaattatgctcaaataacaacatatata 5’**

**TAAACAAACAC---T--AATATATA 5’(78-118) (162-200) _15_TACTATAGATAGAAGATAGATTATGCTCAGATGACAACACAAATATATA**

**:|:||| | :|||:|:|||||||||||| |:|| :||:::|:||**

**AGATGGAGCAC---T--GAACGAGAATACAAACAAT-AGATA 5’ (105-140) pCR3(190-230)** **_06_TTAATGTAGAT**

**AGATAGAGCAC---T--GAACGAGAATACAAACAAT-AGATA 5’ (105-140) bsCR3(192-232) _11_TATATAGAT**

**|:||||::|::||| |:|||:||:|||:|:|||||||||||:|:|**

**_13_TATAGAATATGAGTAAT-AGATCGAAGACAGAGAATAACCTAAAGATA 5’ pCR3(122-166)**

**_09_TTATAGAGTAAT-GAATCAAAGACAAGAGATAATCTAAAAACAATATA 5’ bsCR3(129-167)**

**200 210 220 230 240 250 260 270 280 290**

**uuuuuuuuuuuuauuuuAucauuugauauguguauca*aauuguuauuuauuauuuag*uucguuua*uauuguuauuuuuauaauuuauuuaaguaugc**

**:|||:||:|:||||:|||:||:||||||||| :|||#|:|#||:|#|||||||**

**GAAAGAAGAGAATAGAATGGTGAACTATACAACATATA 5’(190-230) (293-308) _12_TAATTAGT-GAAATGATAGTGATTAGAGTCATACG**

**AAGAGAGAGAAATGGAATAGTAGACTATACACAATAGATA 5’(192-232) (268-312) _05_TAAATAGTAATGGAGATATTGAGTGAATTCGTACG**

**|||:|:||||| ||#|::|||#:|||||::|| |||||||| ||||||||||**

**_12_AATATATATAGT-TTCATGATATGTAATAGGTC-AAGCAAAT-ATAACAATAATATA 5’ pCR3(226-277)**

**_09_TAACAGT-TTGATGATAGGTAATGAGTC-AAGTAAAC-ATAACAATATA 5’ bsCR3(234-265)**

**_14_TT-TCTATGATAGGTAGTAGATC-AAGCAAAT-ATAACAATAAGATA 5’ bsCR3(241-279)**

**300 310**

**aaauaauuuuugu polyA**

**|||||||||**

**TTTATTAAATATATA 5’ pCR3(293-308)**

**TTTATTAAAAATATA 5’ bsCR3(268-312)**

D) C-rich region 4. Resequencing of the mRNA indicated that there were 2 errors in the original sequence (yellow highlights).

**0 10 20 30 40 50 60 70 80 90**

**UAAUUUAUUGUUAUCUUUGUGUAUUUAUUAuuAuuuuAuuuuAAuuuuGGuuGuGC***AuuuuuuuuuuuuuuAuuuG***GuG*UGuuuGuGuuuuA***

**|||:||:|:||||:||:||||:|:||||||| ||||||**

**pCR4(25-64) _12_TATATATAGTAGTGAAATGAAGTTAAGATCAACACG---TAAAAATA 5’**

**bsCR4(25-64) _14_TATATATAGTAGTAAAATGAAGTTAAGATCAACACG---TAAAAATAATA 5’**

**::|::|:| |::|:|:|||:|:|||:||: ||: |||:|||||||||**

**pCR4(48-103) _12_TTTAGTATG---TGGAGAGAAAGAGAATGAAT---CAT-ACAGACACAAAAT-**

**bsCR4(48-103) _12_TTTAGTATG---TAGAGAGAAAAAGAATGAAT---CAT-ACAGACACAAAAT-**

**:|||:||:||:|**

**pCR4(87-134) _10_TTAAATACGAAGT-**

**bsCR4(93-142) _05_TATTAAAGT-**

**100 110 120 130 140 150 160 170 180 190**

**UGuA*C*A*GuuuAuGGuAuAuuuuAuuGuuGuuuuGuuuuuuGuuuuuGuuGUUUGuuUGuGuGGGuAuGuuuuAuuuGuuuuGuuAuAGuuGuuuGuu**

**|:|| ||:|:|:|:|:||||:|||:|||:||::|||||||||||**

**ATATATA 5’(48-103) pCR4(154-192) _14_TAATAGATATATCCATGCAAGATAGACGGAACAATATCAAATTATA 5’**

**ACATATA 5’ (48-103) (166-196) _06_TATGCGTGTAAGATAGATAAAACAATATCAACAAAATATA5’**

**|:|| | | ||:||||:||||:|:||||||||||| ||||::::|||::|**

**ATAT-G-T-CAGATACTATATGAGATAACAACAAATATATA 5’(87-134) pCR4(186-232) _11_TTATATTGGTAAATGA**

**GTAT-G-T-TAGATGCTATATAAAGTGACAACAAAACAAAAAAA 5’(93-142) bsCR4(186-232) _14_TTATATTGGTAAATGA**

**|:|::|:||::|||:|:|:|:|||::|:|:||||||||:||||:|**

**_14_TATATAGTAGAATGAAAGATAGAGACAGTAGATAAACACACTCATATATATA 5’ pCR4(127-171)**

**200 210 220 230 240 250 260 270 280 290**

**uuuuuuuGuuGuUUUG*GGuuGuGAuuuuuuAuuG**GuGuuuuG***AuuGuAuAGuuuAuuuuuuuuGuGACGuuAuAAuuUUGuuuAuuuuuuuuuu**

**:|:||:|:|||:||:|||:||||||:|:||||:|||||||||||||||||**

**||:|:|:||::||||: ||:||||||||||||| p(251-300) _11_TATATTAGATAGAAGAAATACTGCAGTGTTAAGACAAATAAAAAAAAAAA 5’**

**AAGAGAGCAGTAAAAT-CCGACACTAAAAAATA 5’(186-232) _06_TATATTAGATAGAAGAAATACTGCAGTGTTAAGACAAATAAAAAAAAAAAA 5’**

**AAGAGAGCAGTAAAAT-CCGACACTAAAAAATA 5’(186-232) ||:||:|||||:|:||||:|**

**||: ::|::::|::||:|||:: |||:||:: |||||||||||||| pCR4(280-320)_10_TGTAGAATAAATAGAGAAAAGA**

**p(213-261) _12_TAAT-TTAGTGTTGGAAGATAGT--CACGAAGT---TAACATATCAAATATATA 5’**

**_12_TAAT-TCAGTATTAGAGAGTAAC--TACAGAGC---TGACATATCAA-TATATA 5’bsCR4(213-258)**

**300 310 320 330 340 350 360 370 380 390**

**uuAuuuuGuuuuGuGuuuuuuGuAuuG*UUGuuuuuAuUUGGuuuGuuuGGuuuuuuuuuG***UAuuuuuuGUUGuGuuuuGuGuuAuuuuuuGAuuuA**

**:|||:|:|::||||||||||| |:|:|:||:|||:|||:|:|||||:|**

**GATAGAGCGGAACACAAAAAAAAAA 5’(280-320) pCR4(374-417) _11_TATATAGAATACAGTAAGAGACTAAGT**

**:||||:|:||:|:|:|||:: :||::|||||:||:|:|||||||| bsCR4(374-415) _17_TATATAGAATATAATGGAGAACTAAGT**

**_11_TTAAAATATAAGAGATATAGT-GACGGAAATAGACTAGACAAACCATAAAATA 5’(307-351)**

**_09_TTAAAATATGGAAAATATAGT-GACAGAGATGAGCTGAACAAACCAATAAATTAGTTGGTTTGTT 5’ bsCR4(307-352)**

**|::||:::|||:|:|:|: ||:|:|:||||:|||||||||||||**

**pCR4(343-388) _15_TAGTAAGTTAAAGAGAGAT---ATGAGAGACAATACAAAACACAATATATA 5’**

**bsCR4(340-390) _05_TTTAAATGAGTTAAGAGAGAAT---ATAAAGAGCAGCATAAAACACAATAAATATA 5’**

**400 410 420 430 440 450 460 470 480 490**

**uuuuuuAuGuUGuuuuuUGuuuuGGG***UG*GuuuuuuuGuuuuuGuuuuuuuuuuuuGuuuAuGuuuGuuuuuAuuuGuGGuuGuuGuuAuuuuGuuA**

**:|||:||||||||||||| ||||:|:::|:|::|||:|:||:||**

**GAAAGATACAACAAAAAAAAAA 5’(374-417) pCR4(475-519) _12_TTAAATATTGATAGTAATGAGACGAT**

**AGAGAATACAACAAAATATATA 5’(374-415) bsCR4(478-524) _14_TATATTAGTGATAATGAGATAAT**

**||||::::|:||:|:||:||:| || :|||:|||||:|||||||||||||||**

**_14_TAATATGGTAGAAGATAAGACTC---AC-TAAAGAAACAGAAACAAAAAAAAAAA 5’ pCR4(404-457)**

**_08_TAATATAGTAGAAGATAAGACTC---AC-TAAAGAAACAGAAACAAAAAAAAAAAAAA 5’ bsCR4(404-458)**

**|||::|:|||:|:|:|::||||:|||::|||:|||:|||||||||||:|**

**pCR4(442-489) _12_TTAAAGTAGAAAGAGAGAGTAAATGCAAGTAAAGATAGACACCAACAATA 5’**

**bsCR4(442-487) _11_TTAAAATAGAAAGAGAGAGTAAATACAGATAAAGATGAACACCAACAAAATA 5’**

**|||:|::|:||::|:|:||:|||:||:||:|||||||||||||||**

**bsCR4(453-497) _11_TAAAGAGTAGATGTAGATAAGAATGAATACTAACAACAATAAAACATA 5’**

**bsCR4(453-497) _15_TAAGAGATAGATGTAAGTGAGAATAAACACTAGTAACAATAAAACATA 5’**

**500 510 520 530 540 550 560 570 580 590**

**GuuuGGuuGuuGUUGuuAuuUGuGuAuA****GGUUUAuuUAuA*UGCGuuuuuuAuuuuAGAuAAuUAuG****G****UA**UUGGUUUUAUAAAAUG**

**:|:||:||:|||||||||||**

**TAGACTAATAACAACAATAA-TATATA 5’(475-519)**

**CAGACTAGTAACAACAATAAACATA 5’ (478-524)**

**::||:|::|::||||:|:|||||| ||:|:|:||||| ||||||||||**

**_03_TTTAATAGTAGTAATAGATACATAT----CCGAGTGAATAT-ACGCAAAAAAAA 5’pCR4(504-554)**

**_12_TATAGTAGTGATAGATACATGT----CTAGATGAGTAT-ACGCAAAAAAAAAAA 5’ bsCR4(507-554)**

**|| |:|::||:|:||:|:|||||||||:| | #| |||||||||||||**

**pCR4(542-575) _12_TAAT-ATGTGAAGAGTAGAGTCTATTAATGC----C-----T--AACCAAAATATTTAA 5’**

**bsCR4(542-584) _19_TAAT-GTGTGAAAGATGGAATCTATTAGTGT----C----AT---ACCAAAATATTTATA 5’**

**600**

**UUUUUUCU polyA**

E) Cytochrome b

**0 10 20 30 40 50 60 70 80 90**

**GUUAAGAAUAAUGGUUAUAAAUUUUAUAUAAAuAuGuuuCGuuGuAGAuuuuuAuuAuuuuuuuuAuuAuuuAGAAAuuuGuGuuGUCUUUUAAUGUCAG**

**:||:|:||:|::||:||:::|||||||||||||**

**_12_TGATAGGTGTCGTATAGAGTAGTATTTAGGGATAATAAAAAAAAAA 5’ pCYb(32-64)**

**_06_TATAGGTGTCGTATAAGGTAGTATTTGAGGATAATAAAAAAAAAA 5’ bsCYb(32-64)**

**||||||:::|||||||:|:||||||||::::||||||||**

**pCYb(53-91) _11_TTAATAAGGGAAATAATGAGTCTTTAAGTGTGACAGAAAAAAAAAA 5’**

**bsCYb(54-91) _05_TATCAATAGGAGGGGTAATGAGTCTTTAGATGTAACAGAAAAAAAA 5’**

F) Maxicircle unidentified reading frame II

**0 10 20 30 40 50 60 70 80 90**

**UUUUAUAUAGAAAGGUAUAUAAUCUAUAAUGAuuuuAAuGuuuGGuuGuuuuA****AuuuAGuuuuAuuuUUGuGCUUUGAUUGuAGUCGUGUUUUUGA**

**:|||||||::|::::||::|:|| |||||:|||||||||||||||||**

**pMURF2(30-79) _11_TTTAAAATTGTAGGTTAATGAGAT----TAAATTAAAATAAAAACACGAAAGATA 5’**

**bsMURF2(30-79) _08_TTTAAAATTGTAAGTTAATGAGAT----TAAATTAAAATAAAAACACGAAAGATA 5’**

G) NADH Dehydrogenase subunit 3

**0 10 20 30 40 50 60 70 80 90**

**UCAAAAAAUCCUCGCCUUUUUACUUUAGUUUGUUAUCAuuAuuuuuAuAuuuGuuuuUG*A*UAuuGuGGuuuA**UUAuuuuAuuuAuAGGuuuuuuuu**

**|||:||:||||:||:|::::||||:| | |||::||||||| | |**

**pND3(33-76) _12_TATAGTAGTAAAGATGTGGGTAAAAGC-T-ATAGTACCAAAT--ATTATATA 5’ (99-143) _11_TA**

**bsND3(30-73) _12_TATAATAGTGATAAAGATGTGAATAAAGAC-T-ATAACACCAAAT--TATA 5’ (98-141) _12_TAA**

**|||:|:::||| |:|:|:||:|:|:||:|||||:||**

**pND3(63-113) _11_TTAATATTGAAT--AGTGAGATGAGTGTCTAAAAAGAA**

**bsND3(63-108) _09_TTAATATTAGAT--AGTGAGATGAATATTCAAAGAAAA**

**100 110 120 130 140 150 160 170 180 190**

**uAuGuuuuuuAuGuuuuuuAuuGCAuuuuuuuGAuuGuuuuCGuuGuuGuuuGuGGuuuuCGuGuGGuUUGuAuGAuAuGAAuUCACGuuuG*GUGuuuu**

**|||||||:|||||| |||:::|::|:|:||:||:|::||:||||:|||: :||||||**

**ATACAAAGAATACA 5’(63-113) pND3(158-205) _15_TAAGTGTATTAGATGTGCTGTGTTTGAGTGTAAAT-TACAAAA**

**ATACAAAAA-TACATA 5’(63-108) bsND3(158-205) _13_TAAGTGTATTAGATATGCTGTGTTTGAGTGTAAAT-TACAAAA**

**|||:::||||||::|||:|||::||:||:|||||||||||||:| || :|:|:||**

**ATATGGAAAATATGAAAGATAGTGTGAAGAAACTAACAAAAGTATA 5’ pND3(99-143) pND3(190-229) _13_TAC-TATAGAA**

**ATATAGAGAATATAGAAAATGGCGTAAAGAGACTAACAAAAGAATA 5’ bsND3(98-141) bsND3(190-233) _13_TAC-TATAGAA**

**||:|:|:::|||:|:||::|:||||:|:||||||||:||||**

**_18_TATAATTGATGGAAGTAGCAGTAGATACTAGAAGCACACTAAAC-TATATA 5’ pND3(130-170)**

**_11_TAATTAGTAAGAGTGACAATAAACACTAGAGGCACATCAAACATATATA 5’ bsND3(130-174)**

**200 210 220 230 240 250 260 270 280 290**

**AuACAuuGGAuuuAUGuuuuGuuAGuUGuUUGuuuuuuGuAuuGuuAAAuuCCAuUAuuuGuGuUUUGuuGuuuGuuuuuGUGAuA*GuGuuGuuuuAuu**

**|||||| |||||:|:|:||:|:|:::|:|||:|::|:||| ||||||||||||:**

**TATGTATATA 5’ (158-205) pND3(253-299) _14_TCTTTAATAGATATAAGATAGTGAGCAAGAGTATTAT-CACAACAAAATAGTATA 5’**

**TATGTATATA 5’ (185-205) bsND3(253-299) _06_TTTAATAGATATAGAGTGACAGATAGAAACATTAT-CACAACAAAATAATATA5’**

**||||||::|||:|||:||:||||||||||| || :::||:||:|||:**

**TATGTAGTCTAGATATAAGACAATCAACAATATATA 5’(190-229) pND3(284-329) _06_TAAT-TGTAATAAGATAG**

**TATGTAGCTTAGATACAGAGTAGTCAACAAACAATATA 5’(190-233) pND3(285-328) _10_TT-TATAATAAAATAG**

**|:|::||::|::|:|:|||:|||||||:|||||||||||:|**

**pND3(223-263) _11_TTTAGTAAGTAGGAGATATAGCAATTTAGGGTAATAAACATATATT 5’**

**bsND3(222-263) _16_TATTAATAGATAGAGAGTGTAGCAATTTAGGGTAATAGACATATAAA 5’**

**300 310 320 330 340 350 360 370 380 390**

**uuuGuuAUGGuuuuuuGuUUUUGuGGuuuuuGuuuuuuGuuGuAuGuAuAG****GAuuUGuGuGGuAuuuuuGGGAUCAC*GuAuAUUUGUGUGGUGUA**

**|:|:||||:|:|||:|:||||||||||||| pND3(402-438) _13_TATATTGC**

**AGATAATATCGAAAGATAAAAACACCAAAATATA 5’(284-329) bsND3(402-435) _08_TGC**

**AGATAATACCGGAAGATAGAAACACCAAA-TA 5’(285-328)**

**:|::||:|::|:||:::|:|||::||||: ||||::|||||||||**

**pND3(322-369) _13_TTATTAAGAGTAGAAGGTAGCATGTATATT----CTAAGTACACCATAA-TATATA 5’**

**bsND3(322-370) _12_TTATTAAGAGTAGAAGGTAGCATGTATATT----CTAAGTACACCATAAATA 5’**

**|||| :|||::::|:||||:|:||::||||| |||||||**

**pND3(347-388) _18_TATTTTATC----TTAAGTGTATCATAGAGACTTTAGTG-CATATAACAAATGTATA 5’**

**bsND3(355-388) _11_TTT---ATTAAGTGTACTGTGAAAGCTTTGGTG-TATATAACAAACAAATATA 5’**

**400 410 420 430 440 450 460**

**AUUUUAuuuuGuuuAuGA**UGuuuUUUGUUGUAUUAUACAUAUUAUAUUAAUAAAUAUAUAAAA**

**||:||:|:::|||||| ::||||:||||||||||||**

**TTAAGTAGAGTGAATACT--GTAAAAGACAACATAATATTATA 5’ (402-438)**

**TTAAATGAGATAAATGCT--GCAGAGAACAACATAAAAT-ATATA 5’ (402-435)**

H) NADH Dehydrogenase subunit 7

**0 10 20 30 40 50 60 70 80 90**

**UGAUACAAAAAAACAUGACUACAUGAUAAGUAuCAuuuuAuGuuAuuuuuGGuAGuuuuuuuACAuuuGuAuCGuuuuACAuuuG*GUCCACAGCAuCCC**

**:|||||::|:|:|:::||||:|:|||||||||:| |||#|**

**pND7(36-69) _14_TATTATAGT-GAATACGGTGAGAGTTATCAGAGAAATGTAAATAATATA5 (108-137)TTAGATTTTTAGAG**

**bsND7(28-71) _12_TATTATAGTAAGATGCAATGAAAGCCGTCAAGAGAATGTAAACATATAAA 5’**

**||:||||:|:||||:|:|||||||:| ::||||#|||||**

**pND7(59-91) _13_TGTAAGTGTAGATATAGTAGAATGTAAGC-TGGGTGACGTAGATATATA5’**

**bsND7(58-91) _12_TAAAGTGTAAATATAGCGAAGTGTAAAT-CAGGTGACATAAATATATA5’**

**100 110 120 130 140 150 160 170 180 190**

**G***CAGCACAuG**GuGuuuuAuGuuGuuuAuuGuAuuuuuGuGGuGA*AuuuAuuGuuuA**UAUUGAuUGuAuuAuA***G*GuuAUUUGCAUCGUG**

**| #||#||||| |:::|||||||||||||||:|||**

**C----TCATGTAC--CGTGAAATACAACAAATAATATA 5’ pND7(108-137)**

**:||:||:|:|:||:||:|::|||:| ||:|||:||||| |||||||**

**_14_TTAATAAGTGATATGAAGATGCCATT-TAGATAGCAAAT--ATAACTACATA 5’ pND7(124-170)**

**_16_TATATAGTAAATGACATGGAAGTGCTACT-TAAATAACAAAT--ATATATA 5’ bsND7(121-166)**

**||||:::|:| |||::|:||||::||| | :|||||**

**pND7(152-190) _12_TAATAGTGAGT--ATAGTTGACATGGTAT---C-TAATAATACGTAGCATTAAA5’**

**bsND7(151-199) _12_TAAAGTGATAGAT--GTGATTGATATGATGT---C-CAATAA-ATGTAGCATAAA5’**

**200 210 220 230 240 250 260 270 280 290**

**GUACAGAAAAGUUAUGUGAAUAUAAAAGUGUAGAACAAUGUCUUCCGuAUUUCGACAGGUUAGAuuAuGuuA*GuGuuuGuuGuAAuGAGCAuuuGuuGu**

**:||:|:|||||:||:|||||||||**

**pND7(246-269) _14_TAAATAAGGAAATCTATGAGGCTGTTCAGTCTAATACACAACTATA 5’**

**bsND7(246-269) _12_TAAATAAGGAAATCTATGGGGCTGTTCAGTCTAATACACAACTATA 5’**

**|:|||||:|:| :||||:::||||||:||||:||:||||**

**pND7(261-311) _08_TTTTAATATAGT-TACAAGTGACATTATTCGTGAATAACA**

**bsND7(261-293) _10_TTTTAATGTAGT-TATAAGTGACATTGCTCGTGA-CAACA**

**|:|**

**pND7(297-338) _13_TACATA**

**bsND7(292-324) _13_TGAAATAGTG**

**300 310 320 330 340 350 360 370 380 390**

**CuuuA***UGuuuuGAGuAuAuGuuGCGAuGuuGuuuGuCGuuACGuuGuGCAuuuAuGCGuuuAuuAAuuGuA****GAAuuuAC***CCGuAGuuuuA**

**||||| |||| |||||::|||:||:||||||:| |||||||| |#||||||||**

**GAAAT---ACAATATA 5’(261-311) pND7(352-398) _07_TATAAATGTGCAGATGATTAACGT----CTTAAATG---GACATCAAAAG-ATATA5’**

**GAA-T---ATA-TATA 5’(261-293) bsND7(353-402) _13_TAAAATGTGCAGATAATTAATGT----CTTAGATG---GGTATCAAAATTATATATA5’**

**||:|| ::|||:||:||||:|||||||||||||||:| || #|:||::|:||**

**GAGAT---GTAAAGCTTATATGCAACGCTACAACAAATATA 5’(297-338) pND7(390-424)_16_TAATTG---AGTATTGAGAT**

**GAAAT---ATAAGACTCATATACGA-GCTACAACAA-TATATA 5’(292-324) bsND7(391-424) _14_TAATG---GATATTAAGAT**

**:||||::||::||||:||::||::|||||||:||||||||||||:||**

**pND7(327-373) _13_TATTATAGTAAGTAGCAGTGTGACGTGTAAATATGCAAATAATTAATATA 5’**

**bsND7(327-365) _12_TAATTGTAGTAAGTAGCAATGTAACGCGTAGATATGCAAATATACATA 5’**

**400 410 420 430 440 450 460 470 480 490**

**AuGGuuuGuuGuGuAuAuCAuGuAuGGuuuuGG*AuuuAGGuuGuuuGuCUCCGuuG*UUAuGAuCAuuuGAGGAA***CG*UGACAAAuuGAuGACAuu**

**||::|:|:|:|:||||||||||||| |||||:|::|||||**

**TATTAGATAGCGCATATAGTACATAAATTATA 5’(390-424) pND7(486-530) _11_TTTTAATTGTTGTAA**

**TATCAAGTGACATATATAGTACATAACATTAAA 5’(391-424) bsND7(486-530) _11_TTTTAATTGTTGTAA**

**:||||||||:||::||:|:|: ||:|||:|::|||||||||**

**_13_TTATATAGTATATGTCAGAGCT-TAGATCTAGTAAACAGAGGAATATATA 5’ pND7(412-452)**

**_13_TATAGTGTATGTTGGAATC-TAAGTTCGATAGACAGAGGCAAT-A-TATA 5’ bsND7(414-458)**

**:||: |:|::||||:|::|:||| || |:||#|||||||||||||**

**pND7(453-485) _13_TATAAT-AGTGTTAGTGAGTTTCTT---GC-ATTGATTAACTACTGTAA**

**bsND7(453-485) _13_TATAAT-AGTGTTAGTGAGTTTCTT---GC-ATTGATTAACTACAATAA**

**500 510 520 530 540 550 560 570 580 590**

**uuuuGAuuuAuG**UUGuGGuuGuCGuAuGCAuuuGGCUUUCAuGGuuuuAuuA*GGuAUUCUUGAUGAuuuuGuuuuuGGuuuuGuuGAuuuuuuGuuG**

**|| :||:|||:|:|:|:|::|||:|:::||:||:|:|||**

**AATA 5’(452-501) pND7(564-615) _12_TTTATTAAGATAGAGATTAAAGCGGTTAGAAGATAAC**

**ATA 5’(453-485) bsND7(564-615) _10_TTTATTAAGATAGAGATTAAAGCAGTTAGAAGATAAC**

**||:|||:|:||: |:||::||||||||||| |:|::||||:|:::||**

**GAGACTGAGTAT--AGCATTAACAGCATACGAATATA 5’(486-530) pND7(584-630) _15_TATAGTTAAAGAGTGAC**

**GAGACTGAGTAT--AGCATTAACAGCATACGAATATA 5’(486-530)**

**|||: |:||:::||:|:||::||:|::|||||||||:||| ::|:**

**_14_TAATTGTATAT--AGCATTGACGGTATGTGTGAGTCGAAAGTACTAAATATA 5’(508-548) pND7(596-642) _18_TAAATTTGAT**

**_11_TTATAT--AGTGCTAGTAGTATATGTAGGTTGAGAGTACCAAAATAATATA 5’bsND7(508-553) bsND7(596-640) _11_TTAAT**

**|||:|||||::||:||||:||:|:|||| |:|||||||||||||**

**pND7(526-569) _12_TAATATGTAAATTGAGAGTATCAGAGTAAT-CTATAAGAACTACTATATATA 5’**

**bsND7(526-569) _13_TAATATGTAAATTGAGAGTATCAGAGTAAT-CTATAAGAACTACTATATATA 5’**

**||||:::|:|||:| |:||:|||::||||||||||||**

**pND7(540-576) _05_TACTGATAGTATTGAGATAGT-CTATGAGAGTTACTAAAACAAATAATAATA 5’**

**bsND7(540-571) _14_TAATCGTTAGTGTCAAGATAGT-CTATAAGAACTACTAAATAATATA 5’**

**600 610 620 630 640 650 660 670 680 690**

**uuGuuGA***UAAuAuCAuGuuuGuuuGuuAuGGAuuGuuAuGAuuuGuuAuuuGuGGGuAAUCGuuuAuuuUAuuuGCGuuuGC***GuGGuuuGuCAu**

**||||||| |||||| :::|||||::|||:|||||:|||::|||| ||:|||||||||**

**AACAACT---ATTATATT 5’ pND7(564-615) pND7(656-699) _12_TTTTATTAGTGAATGAAATAGACGTGAATG---CATCAAACAGTATATATA 5’**

**AACAACT---ATTATATA 5’ bsND7(564-615) bsND7(666-701) _12_TACATAGTTAGTGA-TAGAATAAGTGCAGATG---TACCAAACAGTAGATATA 5’**

**|:::||| |||||||||:||||||||||| :|||:| :::|||::|||:**

**AGTGACT---ATTATAGTATAAACAAACAATTAATA 5’(584-630) pND7(679-727) _13_TTAAATG---TGTCAAGTAGTG**

**|::|::| ||||||||:||:|:|:||:|||||||||||||: bsND7(679-725) _16_TGATGTTAAGTG---TGTTAGACGGTA**

**AGTAGTT---ATTATAGTGCAGATAGACGATACCTAACAATATATA 5’(596-642)**

**AGTGACT---GTTATAGTGCGAGTGAACAATACCTAACAATTAAA 5’(596-640)**

**|||::||:::|||||:|::||||:|||:|||||||||||||:**

**_13_TATATTTAGTGATACTGAGTAATAGACATCCATTAGCAAATAGTATA 5’ pND7(629-670)**

**_11_TTTTAGTAGTATTAGATAGTAAGTGTCCATTAGCAAATAAAATATATA 5’ bsND7(632-674)**

**700 710 720 730 740 750 760 770 780 790**

**uuuuuGAuuuAuAuGAuuuA**GuuuuuA**A**UAGuuuAAGuGGuGuuuuGuCuCGuuCGuuAGGuAuGGuGuGAGAuuGUCGuuuAuuuAGuuGuuA**

**::|||:|||||||||||||| |||||| ||::||:||:|:|||::||:||**

**GGAAATTAAATATACTAAAT--CAAAAAAAAAAA 5’(679-727) pND7(778-830) _12_TATAGTAGTAAGTGAATTGACGAT**

**GAAGGTTGAATATACTAAAT--TAAA 5’(679-725) bsND7(772-806) _12_TAATTGATATGACACTCTAGTAGCAAATAGATCAACAAT**

**bsND7(782-816) _16_TAATTGATGTTATATTCTAAGGGCAGATAGATCAACAAT**

**|||:|:|:| :|:|:|| | ||:|:||||||:|:||||||||||| |:||:|:|**

**_13_TAATATTGAGT--TAGAGAT--T--ATTAGATTCACTATAAAACAGAGCATATATA 5’(711-758) (792-845) _10_TTTAATAGT**

**_13_TATATATTGAGT--TAGAGAT--T--ATCAGATTAACCATAGAACAGAGCAAAA 5’ (709-741) (790-839) _11_TAATTAGTGAT**

**|:|| | ||||:|||:||:|::|:||:|||||||||||**

**pND7(725-764) _14_TAACTTAGAT--T--ATCAGATTTACTATGAGACGGAGCAAGCAATAATATA 5’**

**bsND7(722-765) _14_TATAAGAGT--T--ATTGAGTTCGTTACAAGATAGAGCAAGCAATTATATA 5’**

**|:|||:|:||:||::||:|:||||:||||||||||||||**

**pND7(756-794) _14_TAATTATAGTGTAAGTAGTCTATGTCATATTCTAGCAGCAAATAAATCATA 5’**

**bsND7(756-794) _14_TTTATAGTGTAAGTAGTCTATGTCATATTCTAGCAGCAAATAAATCATA 5’**

**800 810 820 830 840 850 860 870 880 890**

******UGA*****GuUGuAuuuuAuGuuuuGuuAuGAuuAuuGuuuuuGuuuuAuAGGuGAuGCAuuuGA*UCGuuuAuuuuuACGuuuGuuuGAUAuGCG**

**::| ||::|||||||||||||:| :|||||||:|||:|||::|:|||||:|:**

**----GTT-----CAGTATAAAATACAAAATATATA 5’(778-830) pND7(872-916) _09_TTAAATAAAGATGTAAATGAGCTATATGT**

**----ACT----ATATA 5’(772-806) bsND7(872-919) _17_TTAAGTGAAGATGTGGGTGAATTATATGC**

**----ATT-----CAATA 5’(782-816)**

**::| ::||:|:|||||:|:|::|:||||||||||||||**

**----GTT-----TGACGTGAAATATAGAGTAGTACTAATAACAAAATATA 5’(792-845)**

**----ATT-----TAGCATGAGATATAGAACAATACTAATATATA 5’(790-839)**

**:|||||::::|:|:::|||:|:|::||:||||||| :||||||||**

**_13_TTTAATAGTGGAGATGGAATGTTCGTTATGTAAACT-GGCAAATATA 5’ pND7(834-877)**

**_15_TTTAGTAGTAAAAATAAGATATTCACTATGTAGATT-AGCAAATAAATATA 5’ bsND7(834-879)**

**||||:| ||::||||||:|||||:|:|:||||||||:**

**pND7(863-902) _10_TTAAATT-AGTGAATAAAGATGCAGATAGACTATACGT**

**pND7(866-902) _10_TATT-AGTGAATGAAAATGTGAGTAGATTATACGC**

**900 910 920 930 940 950 960 970 980 990**

**uAuGAGuuuGuuGAuuuGuAAGCAAuGuuuuuuuGuuGGuuuuuuuGuuuuuG*****GuuuuGuuuGuuuGuuuG**AuuAuuuAuAuuGuGAuAuuAC**

**||| |||::|:|:|:|:|::| |:|||:||:|:|||||||||||**

**ATAATATA 5’(863-902) pND7(959-1000) _14_TAAAAGTAGATAGATAGGC--TGATAGATGTGACACTATAATG**

**ATAAATAATATA 5’(866-902) bsND7(959-1000) _11_TAAAAGTAGATAAATAGGC--TGATAGATGTGACACTATAATG**

**||:||:||||||||||| |||||:|:::||||:||**

**ATGCTTAAACAACTAAAATATA 5’ gND7(872-916) pND7(983-1017) _12_TAATATGATGTTATAGTG**

**GTGCTTAAACAACTAAACATA 5’(872-919)**

**||:|:|:|:|::||:|||||||||||::|||||:||:|| |||||**

**_13_TATTTAGATAGTTAGACATTCGTTACGGAAAAATAATCATATATA 5’ pND7(901-939) pND7(1001-1032) _08_TAATAGTTAATG**

**_12_TATTTAAGTGACTAGATATTCGTTATAGAAAAACAATCATATATA 5’ bSND7(901-939) bsND7(1000-1043) _13_TTTAATA**

**|::||:|:||::|||||||:||:||:|||||||||:|||:|**

**ATGTTCTAGTAATTGAATGTTCGTTATAAGAAGACAACCAAAGAAATA 5’ pND7(907-947)**

**_17_TCTAGTAATTGAGTGTTCGTTATAAGAAGACAACCAAAGAAATAGAAAACGGAACC 5’ bsND7(907-951)**

**||:|:::|:||:|:||:|::| |||:|:|:|||||||||| |**

**_13_TAATAGTTAGAAGAGCAGAGGC-----CAAGATAGACAAACAAAC--TTATATA 5’ pND7(932-978)**

**_12_TTAATTAAAGAGATAGAGAT-----CAAAGTAAGCAGACAAAC--TAATAATATATA 5’ bsND7(934-983)**

**1000 1010 1020 1030 1040 1050 1060 1070 1080 1090**

**CAuuG****AGACCAuuAuuAuGuuAuuuuAuAGuuuGuGGuGuuGuuGuuuGCCGGGuAuA*UCAuuuGC*UUGUGuuGAACACCCCAAAGGuGA***G**

**| :::|||| ||||:|:| :|:|:::|||||||## ::||| |**

**GAAATA 5’(959-1000) pND7(1055-1085) _03_TTTTATAT-AGTAGATG-GATATGGCTTGTGGAAAGATTACT C 5’**

**GAAATATA 5’ (959-1000)**

**|||:: |||||||||#|||||||||| :||||||#|#||:||:| |**

**GTAGT----TCTGGTAATTATACAATAAATATATA 5’ pND7(983-1017) (1089-1121) _14_AAATGAGTGTTTTGTGGAG-TTTCATT---C**

**#||:| |||||||:||:|:||||||||||| bsND7(1087-1113) _12_TAAATG-AATATAGCTTATAGAGTTTCTGCT---T**

**ATAGC----TCTGGTAGTAGTGCAATAAAATATATATA 5’ pND7(1001-1032) :**

**GTGAC----TTTGGTGATAGTGCAGTAAGATGTCAAACACCACATATA 5’ bsND7(1000-1043) pND7(1099-1143) _14_TAT**

**||:| |:|||#||||:||:|||:|||||||||||||:||| bsND7(1099-1143) _12_TAT**

**_13_TAGC----TTTGGGAATAGTATAATGAAATATCAAACACTACATA 5’ pND7(1015-1043)**

**_14_TT----TTTAGTGATAGTGCAGTAAGATGTCAAACACCACATATA 5’ bsND7(1013-1043)**

**|:||::::||||:::|:|||:|||:|||:| |||||#|| ||||:**

**pND7(1032-1067) _11_TTTAAGTGTCACAGTGATAAATGGCTCATGT-AGTAA-CG-AACATATATA 5’**

**bsND7(1032-1078) _10_TTTAAATGCTATAGTGACGAGTGGCTTATGT-AGTAAACG-AATATAAA 5’**

**1100 1110 1120 1130 1140 1150 1160 1170 1180 1190**

**uAuuGuuuGuuAuuA****UGuuuuuGuGuuGGuuuAuGuuCUCGuuuACGuuuGCGuuGuGCGGAuuuuuuGCA*UAUUUGuuuAuuGGAuGuuuGuuu**

**|||||||||||||:| |:| :|||||::|||:|:|:|||**

**ATAACAAACAATAGT----ATATA 5’(1089-1121) pND7(1181-1218) _12_TTAAATAGTCTATAGATAAA**

**ATAACAAACAATAA-----ATA 5’ (1087-1113) bsND7(1183-1224) _13_TAATAGTGACTTATAAGTAGA**

**|||:::||||||:|| ||::|:|:||:|:||||||||||||**

**ATAGTGAACAATGAT----ACGGAGATACGATCAAATACAAGAGAATATA 5’(1099-1143)**

**ATAGTGAATAATGAT----ACGAAGGTACAGTTAAATACAAGAGAATATA 5’(1099-1143)**

**|:|||:|| |:|:|:|:::|:::|:|||:|||||||||||:|||:|:|**

**_13_TATAATGAT----ATAGAGATGTAGTTAGATATAAGAGCAAATGTAAATGTATA 5’pND7(1107-1157)**

**_14_TATAATAGT----ATAGAGATACAGTCAAGTGTAAGAGCAA-TGCAAAT-TATA 5’bsND7(1107-1146)**

**_09_TCAATTGAGTATAAGAGCAGATGCAAGCGTAACATGCCTAATATATA 5’ bsND7(1128-1167)**

**:|||:|:|::|:|::||:|:|::|| ||:|:||:||||||||||||:|**

**pND7(1150-1197) _10_TTAAATGTAGTATGTTTAGAGAGTGT-ATGAGCAGATAACCTACAAATATA 5’**

**bsND7(1150-1195) _10_TATATAAGTGTAATATGCCTGGAAGATGT-ATAGACAAATAACCTATAAA 5’**

**1200 1210 1220 1230 1240 1250 1260 1270 1280 1290**

**GCGuGGuuuuuuAuuGCAuGAuuuAGuuGC***C*GuuuuAGGuAAuAuuGAuGuuGuuuuuGGAuCCGUAGAUCGuuA*GuuuuAuAuGuG**A*******

**|||||:|:||:|||||||| ||:|||:|:| ||:|||||:::| |**

**CGCACTAGAAGATAACGTAAATATATA 5’(1181-1218) pND7(1269-1320) _12_TAATTTAGTAGT-CAGAATATGTGC--T-----**

**TGTATCAGAAAATGACGTACTAAATATA 5’(1183-1224) bsND7(1269-1320) _17_TAATTTAGTAGT-CAGAATATGTGC--T-----**

**|||||:||::||:|||||:| | |||:||:||||||||||||||||**

**_14_TAATAATGTGTTAGATCAATG---G-CAAGATTCATTATAACTACAACATATA 5’pND7(1210-1257)**

**_09_TAATGTGTTAGATCAATA---G-CAAGATTCATTATAACTACAACATATA 5’bsND7(1233-1257)**

**|:::|||:|||::|:||:|||||||||| |||**

**pND7(1251-1282) _12_TAAGTGTGACAGAAATTTGGGTATCTAGCAAT-CAA-TATATATA 5’**

**bsND7(1240-1270) _16_TTTTATTGTAGTTATAGTGAAGACTTAGGCATA--GCAAT-CAAATATA 5’**

**1300 1310 1320 1330**

***GGUUAUUGuAGGAUUGUUUAAAAUUGAAUAAAAA**

**|:|:||||||||||||||||**

**-CTAGTAACATCCTAACAAATATA 5’(1269-1320)**

**-TTAGTAACATCCTAACAAATATA 5’(1269-1320)**

I) NADH Dehydrogenase subunit 8

**0 10 20 30 40 50 60 70 80 90**

**CAAUUUAAUAAUUUUAAGUUUUGGUUGAUUAuuAuuuuuuuAuuuuuuuAuuuuuGuAuGuuuuuuuuuGAuuuuuuGuuuuuuuUUUUUGuuuGuuuuu**

**|||:|:|||:||||:|||:|||||:||||||||||||||| |||::|::||:||**

**pND8(29-68) _08_TATTATATAGTGAAAGAATAGAAAGATAAAGACATACAAAAAAAAAAA 5’ (87-136) _14_TAAATGAGTAAGAA**

**bsND8(28-56) _04_TAGGGAGATAGTAAAAGAGTAGGAGGATGAGGATAAAA 5’ bsND8(86-139) _12_TAAAATGAGTAAGAG**

**:|||:|:|:|:||::||||:||||||:||:||||||||||||||**

**pND8(55-98) _11_TTATATAGAGAGAAGTTAAAGAACAAAGAAGAAAAACAAACAAAATATA5’**

**bsND8(54-97) _16_TATATATAGAAAGAGACTGAGAAACAAAAGAAAAAGACAAACAAA-TATA 5’**

**100 110 120 130 140 150 160 170 180 190**

**AuAuGuGUuuuGuuuGuuGuGuuA****CuAUUU*GuuuA***CCCAuuGAGuuAACCAuuGuuAGuuuAuuGGuuCGuGGUAACCAuuuuuuGCGUUUU**

**|||:||::|:||:|::|||||||| |||||| :| :|||:|:|||::||||:|:||||||||#||||||||||**

**TATGCATGAGACGAGTAACACAAT----GATAAA-TA 5’(87-136) (161-187) _11_TTAATTAGATAGTCAAGTATCATTGGTATAAAACGCAAAT**

**TATATGCAAGATAGGCAGTACAAT----GATAAA-CAAATATA 5’(86-139) _15_TGTAATTAAGTAGTTAGGTATCATTGGTAAAAGACGCAAAA**

**:||::|::::||| |||:|| :|::| |||||||||||#|||||| ||||:|::|::|||**

**(111-153)_13_TTAAGTAGTGTAAT----GATGAA-TAGGT---GGGTAACTCAAATGGTAAATATATA5’ p(186-230) _13_TTAAAGAGTGTGAAA**

**_12_TATTCAGATAGTATAGT----GATAGA-CAGAT---GGGTGACTTA-TTGGTAACATATA5’ pND8(187-228) _11_TAAAAGAGCGTAAAA**

**:|:||| |||:|: :||:| |#||:|:|:|:|||||||||||||||||**

**pND8(117-170) _11_TTTATAAT----GATGAG-TAAGT---GAGTGATTTAGTTGGTAACAATCAAATAAACA 5’**

**200 210 220 230 240 250 260 270 280 290**

**uAUU***GGuGuGGuuuAGAGCGuuGuAuuGCuuGuCGuuuAuGuGAuuuAAuuuGCCCuA****GuuuAGCAuuGGAuG***UUCGuGuuGGGuGGAGu**

**AATATA 5’ pND8(161-187) :|:: |:|:|:||:|||::|:|**

**TGATATA 5’ bsND8(160-199) pND8(276-318) _13_TAATTGT---AGGTATAATCCATTTTA**

**|||: |:|:||:||:|||||||||||||| bsND8(289-318) _10_TTTTGT---AAGTAGAGCTCATTTCA**

**ATAG---CTATACTAAGTCTCGCAACATAACATA 5’(186-230)**

**GTGA---CCATATTAAATCTCGCAACATATATTATA 5’(187-228)**

**:||:|||:|:|::||||:|:||||||||||||**

**_06_TAATAGCTATAGTAAGTCTTGTAGTATAATGGACAGCAAATACAA 5’ (213-244)**

**_12_TTAGATGTTGCAGTATAATGAGTAGCAAATACATATAAA 5’ bsND8(219-245)**

**:||||::|:|:|:|||:|:||||| :|| :|||||||||**

**pND8(237-267) _15_TTAAATGTATTGAGTTAGATGGGAT----TAATATGTAACCTAC 5’**

**bsND8(246-271) _11_TAATGTAGTGAGTTAAATGGGAT----TAGATTGATACCTAC---AAGCATA 5’**

**|||:|:|:|:|||:|:||||| |||:||#||:||||| |||:|:**

**pND8(240-288) _10_TATATATTGAGTTAGATGGGAT----CAAGTCATAGCCTAC---AAGTAT 5’**

**bsND8(259-285) _05_TATAATGTTAGTATATTGAGTTAGATGGTAT----TAGATCGTAGCCTAC---AAGAATATA 5’**

**300 310 320 330 340 350 360 370 380 390 uuuGGuGGuCAU**C*GuuuuGCGGAuuGAuuuACAuuGAGuuAU*C**GU**CGuuGuAuuuAuuGuGGuuuuuGuAuGCAuGuuuGCCCGACAGAU****

**:|::|||:|||| | ||| |||:||::|||:|:|:|||||#||||**

**GAGTCACTAGTA--G-CAACATATATA 5’(276-318) pND8(372-417)** **_14_TAAATATGTGTATAGATGGGCTTTCTA--**

**GAGTCATCAGTA--G-CAATATATA 5’(276-318) (390-414) _15_TAT-TGTGTGTGAGCGAGTTGTTTG--**

**|||::::|||| | :|:|::|::||:|||:||||||||||||| |||||**

**TAACTGTTAGTA--G-TAGAGTGTTTAGCTAGATGTAACTCAATATATA 5’(301-344) (391-431) _14_TAAAATTTAATTACGTGTTAGTCTA--**

**_11_TAATCGTTAGTA--G-TAAGATGCCTAGCTAGATGTAACTCAATATA 5’(301-344) bsND8(407-441)_15_TATAT--**

**:|#|| | :||:|:|:|||::||:||||:|||||||| | :| |**

**_13_TATAATA--G-TAAGATGTCTAGTTAGATGTGACTCAATA-G--TA--GATTATA 5’ pND8(310-353)**

**_14_TTA--T-CAGAGTGTCTAATTAAGTGTAACTCAATA-A--CA--TATA 5’ bsND8(316-344)**

**|:||||:||:|||| | :: ||||||||||||**

**pND8(331-364) _14_TAATGTCTATAGT--AGTGTAGCTTAATA-G--TG--GCAACATAAATATATA 5’**

**_17_TATAGTAATAGAGTGTGTGATTGGATGTAGTTCGATA-G--CA--GCA 5’bsND8(325-355)**

**:|:|||| | :| |::||||:|||:|:||:||:||||||||||**

**pND8(338-382) _09_TTTTAATA-G--TA--GTGACATGAATGATACTAAGAACATACGTAAATATA 5’**

**bsND8(338-385) _04_TATTTTTAATA-G--TA--GTGACATAGATAATATCAGAGACATACGTACAACATGTA 5’**

**400 410 420 430 440 450 460 470 480 490**

****GCCAuuACGCAUUCAuuGuuuGuuAuGuGuuuuuGuuGuuuAGCC**AU**GuAuuuAuuG*GCGC***C***CAAGuuuuuAuuGuuuGGuuGuuGu**

**:||||||||||||#|| bsND8(465-503) TTTTATAAGTGTC-AGTG---G---GTTCGAAGATAGTAAACTAACAACA**

**--TGGTAATGCGTAAATATATA 5’(372-417) |||| :|:| # # |:|||:||||:||::|||:|:||**

**--TGGTAATGCGTAA-TATATATGTTAAA 5’(390-414) pND8(477-512) _14_TAAC-TGTG---A---A#TTAAAGATAATAAGTCAATAGCA**

**:||||||#:||::|||||||||||||:|:| bsND8(482-510) _12_TATAGTAATGAGCCAGTAACG**

**--TGGTAATATGTGGGTAACAAACAATATATATA 5’ (391-431) ||::|::|:::**

**||::||:|:|:||:||||||||:||||||||| pND8(489-531) _14_TAATTAGTAGTG**

**_12_TATATGCAGTGGGTGATAGACGATACACAAGAACAACAAAAAAAA 5’ (411-442) bsND8(494-539)_18_TTATAGTG**

**--GGAATATGTGTAGGTAGTAAATAATATACAAAAACAACAATAAA 5’(407-441)**

**||:|:||:|::||:|:||:|| || :|||:||||: |||# | ||||||||**

**pND8(426-466) _13_TTATATAAGAGTAATAGATTGG--TA--TATAGATAAT-CGCA---G---GTTCAAAATA 5’**

**bsND8(426-480) _02_TTATATAGAAGTAGTGAATTGG--TA--TATGAGTAAC-TGCG---G---GTTCAATATATA 5’**

**500 510 520 530 540 550 560 570 580 590**

**uuuAuGuuAuuuGAuuuuuAuuuGuGuuuuGuGuAGuuAuuuAuuuuGGGuGAuuuAuuGUGuuuAuGAuuuAA***AGAA**AuuCACGGUGAAAUUAA**

**AAATTATA 5’ (465-503) ||||::|:|:||::||:||| |||| ||||||||||||||||**

**:|||||||||||: pND8(554-598) _13_TAATAGTATAGATGTTAGATT---TCTT--TAAGTGCCACTTTAAT 5’**

**GAATACAATAAATATATA 5’(477-512) bsND8(554-598) _05_TAATAGTATAGATGTTAGATT---TCTT--TAGGTGCCACTTTAATATATA 5’**

**AGATACAATAATTATATA 5’ (482-510)**

**|:|||:|:|:||||::||||||||||||||:|**

**AGATATAGTGAACTGGAAATAAACACAAAATAGATA 5’ (489-531)**

**||||::|:|:||:||||:||||::|||:|:|||||||||||**

**AAATGTAGTGAATTAAAGATAAGTACAGAGCACATCAATAATATATA 5’ pND8(500-540)**

**AAATATGATAGACTGAGAATAGATACAAGACACATCAATA-TATA 5’(494-539)**

**bsND8(523-567) _12_TATAGAGTATATTGATAGATAGAGCTCACTAAATGACACAAATATAAATA 5’**

**600 610**

**AUUUUGACUAAAU poly[A]**

J) NADH Dehydrogenase subunit 9

**0 10 20 30 40 50 60 70 80 90**

**UUAAUAUCAACUUAAUUUUUUUUAUAAACAuuAuAuuAUGuGuAuAuUUUUAuGuuuAuuuCGuuuAuGuuuuuGuuuAAuuUUAuuuuA**UUGuuuGu**

**:||:||:::||||:||:||||:|:|||||||||||||||**

**pND9(33-71) _14_TATTATAGTAGTATGTATATGAAGATACGAGTAAAGCAAATACAAATATA 5’**

**bsND9(25-72) _11_TTTTGTAATATAGTGTATATGTGAGAATATAAATAAGGCAAATACAAAATATA 5’**

**||::|:||:||:|:::||||:||||||:|| ||||||||**

**pND9(60-105) _11_TATTTAGTGAGTATAAGAGTGAATTGAAATAAGAT--AACAAACA**

**bsND9(60-101) _11_TAAATGTAGTGAATATGGAGATAGATTAAGATAAAAT--AACAAACA**

**||| |:::||:|**

**pND9(87-124) _11_TTAAT--AGTGAATA**

**bsND9(87-124) _10_TATAAT--AGTGAATA**

**100 110 120 130 140 150 160 170 180 190 GuuGuAGAuGGuGuuUUGuuuGuuuuGuuGAuuGuAGuuuuuuGuuuuuuuAuuGuuuuGuuAGuuuuuuuuuGuuuuAUUGuAuGuuuuuAuuuuuuAA**

**|||:|| ||||:::|::|:|:|||:::||||**

**CAATATA 5’(60-105) pND9(176-216) _14_TAATAGTGTGTAGAGATAGGGAATT**

**TA-TATA 5’(60-101) bsND9(176-216) _14_TAATAGTATATAGAGATAGAAAATT**

**:|::||||::|||:|:|:||||||| ||||||**

**TAGTATCTGTCACGAGATAAACAAA-CAACTATA 5’ (87-124)**

**TGACATCTACTACAGAGCAGACAAA-CAACTATAAA 5’ (87-124)**

**:|||:|:|::||:|:|||||::||:|:|||:|||||||||||:|**

**pND9(117-160) _13_TTAAATAGAGTAATTGACATCGGAAGATAAAGAAATAACAAAATA-TATA 5’**

**bsND9(117-162) _12_TTAAATAGAATAGTTAGTGTCAAAGAGTAAAGAAATAACAAAACAATATA 5’**

**:|||::|:||:|||||||::||:|:||||||||||||||||||||**

**pND9(149-193) _15_TTGATAGTAGAATAATCAAAGGAAGATAAAATAACATACAAAAATAATATA 5’**

**bsND9(147-187) _15_TAGAATAGTGAGATAATCAAAGAGAAGCAGAATAACATACAATATATA 5’**

**200 210 220 230 240 250 260 270 280 290 uuuGuGAuuuuuGuuuuuAuAuuGUUGuGAuUUGuuAuuGAuuGAuuuuuGuGGuuuuuGuuuuuGuCGuuuuAuGuuGuuGUAuAuuuuAuuuuGuuuG**

**|:|||||:||||||||| |||||:::|:||:|:||||:|:||:|||**

**AGACACTGAAAACAAAATATAACATA 5’(176-216) pND9(272-312) _06_TATACAGTGATATGTGAAATGAGACGAAC**

**AGATGCTAAAAACAAAATATAACATA 5’(176-216) bsND9(273-314) _12_TTTATAGTAGTATATAAGATGGAACGAAT**

**||:|:|:||:|:|:|:|||||::|::||||:|||||||||||**

**TAATATTGAAGATAGAGATATAGTAGTACTAGACAATAACTAAAATATA 5’ pND9(201-242) pND9(303-339) _13_TAA-**

**_11_TAATTAAGAGTAGGGATATGGTGACACTAGATAGTAACTAACTAAAAAAAA 5’ bsND9(204-249) bsND9(305-339) _13_TAAT**

**:|||:||:|:|:||:::|||:|:||:|||||:|||||||||**

**pND9(239-279) _12_TTTAATTAGAGATACTGGAAATAGAAGCAGCAGAATACAACATATTAAA 5’**

**bsND9(239-286) _11_TTTAATTGAAAGTACTAGAGATAAGAGTAGCAAGATACAACAATATATA 5’**

**300 310 320 330 340 350 360 370 380 390**

**uuuuuGuGuGuuCGuuuGuGuuuuGuuuuGuGuuGUUUGuuuGUAuuuuuuGGAuuGuGuuuuA*GuuuuA**GuuGuuuuuGuUAuGCGuuuuuGuuGu**

**|:|:||||||||| :||::|:|:|:| |||:|| :|::|||:||||||||||||**

**AGAGACACACAAGAATA 5’(272-312) pND9(352-392) _13_TAATTAGTATAGAGT-CAAGAT--TAGTAAAGACAATACGCAAATTATA 5’**

**AAAGACACACAAGCATATATA 5’ (273-314) (352-392) _10_TAATTAGTATAGAGT-CAAGAT--TAACAGAGACAATACGCAAATTATA 5’**

**:||||::|||:||||||:|:|:|:||||||||||||| :||||:|:|:|:|:|::|**

**AGTGACACGTAAGTAAACACGAGATAGAACACAACAAACATATA 5’(303-339) pND9(382-421) _10_TTAATATGTAGAGATAGTA**

**AGAGTCACGTAAGTAAACACGAGATAGAACACAACAAACATATA 5’(305-339) bsND9(380-418) _12_TAATAGTATGTAGAGATAGTA**

**:||||:|:||:|::|||:|:||||||:|:||:||||||||||||| :|**

**pND9(319-366) _11_TTAAAATAGAATATGACAGATAAACATGAGAAGCCTAACACAAAAT-TATA 5’**

**bsND9 (343-368) _03_TTAAAATAGAATATGACAGATAGAGATAAAGAATCTAACACAAAAT-TAAATA 5’**

**400 410 420 430 440 450 460 470 480 490**

**uGGAACGC*GAAuGuuuUGAUUUGuuuGGuuuuUAuuuuGuuGGuAAuGAuAuuuuACAUCGuuuAuuuGuuGAuuG****GuuuuuuGuuGGuuuuuuu**

**::||||:| ||||:|||||||| |:||:|:|| :|:|:|::|||||:|:|:|**

**GTCTTGTG-CTTATAAAACTAA 5’(382-421) pND9(468-514) _13_TATAATTGAC----TAGAGAGTAACCAGAGAGA**

**ATCTTGCG-CTTACAAAATATATA 5’(380-418) bsND9(469-514) _11_TTAATTGAC----TAAAGAGTGACCGAAGAAG**

**:|||:|:||:|:||||:|:|:||:||||:||||||||||**

**_08_TTTTATAGAATTGAACAGATCGAAGATAAGACAACCATTAAAATATA 5’ pND9(409-447)**

**_05_TATATTGTAGAATTAAGTGAATCAGAAATAAAGCAACCATTAAAATATA 5’ bs(410-447)**

**:|||::|||:|||:|:||||||:||||:|:|||||||| ||||**

**pND9(439-484) _13_TTAACTGTTATTATGAGATGTAGTAAATGAGCAACTAAC----CAAATATA 5’**

**bsND9(438-483) _10_TATAATTGTTATTGTAGAATGTAGCGAGTGAATAACTAAC----CAA-TATATA 5’**

**500 510 520 530 540 550 560 570 580 590**

**uuGuuGAAGuGuuAUCCAuuAuuuGGuuuGuuuGuAuuGuuAuuuuGuGuGuuG**GuGGAGGAGAUAGuAuGuACGuuuACAAuGuuAuuuuuGuuGuu**

**::||||||||||||| ::||:|||:|:||||||:||||:|:|||||||**

**GGCAACTTCACAATATATA 5’(468-514) pND9(568-604) _11_TGCTATAGTGTATATGTAGATGTTATAATAGAGACAACAA**

**AGCAACTTCACAATATATA 5’(469-514) bsND9(569-600) _04_TGAGTAGTATATGCAAATGTTACAGTGAAAACAACAA**

**:|||||:::|:|||||||||:||:||:::|||||||||||||||:|:| |||:|:|:||:|:||||:**

**_12_TTAACTTTGTAGTAGGTAATAGACTAAGTGAACATAACAATAAAATATA 5’(502-549) pND9(582-612) _09_TTTATAGTGAAGATAACAG**

**_14_TAACAGTTTGATGATAGGTAATGAGTCAAGTAAACATAACAATATATA 5’(509-542) bsND9(582-611) _12_TTTATAATGGAGATAGTAA**

**bsND9(597-625) _12_TTTATAGTAGAGATATTAA**

**||||:|:||:||:|:|::|: |::||:|||||#:|||||||||||**

**pND9(534-566) _14_TAATAATAGTAGAATATATGAT--CGTCTTCTCTAGTATACATGCAAA 5’**

**bsND9(533-566) _09_TTATAATAGTAGAATATATGAT--CGTCTTCTCTAGTATACATGCAAATAAA 5’**

**|||:|:||:||:|:|||:: :|:|||:|||:||||||||||||**

**pND9(535-578) _13_TTAATAGTAGAATATACAGT--TATCTCTTCTGTCATACATGCAATTATA 5’**

**bsND9(543-580) _09_TAATAGTGAACATATAGT--CATCTCTTTTATTATACATGCAAATTATA 5’**

**600 610 620 630 640 650 660 670**

**GCAuACC**AAuuUUUAuuuG*CAuuAuuuuAuuuA***AuA**UCACCGuUGUAAUUCUAAAUUUCUCACUUCC**

**|||||**

**CGTATATATA 5’(568-604)**

**TATATA 5’(569-600)**

**:|||||| ||||##|||:|| ||||||**

**TGTATGG--TTAATTATAGAC-GTAATATA 5’(582-612)**

**CGTGTGG--TTATTAATAGAC-GTAA 5’(582-611)**

**CGTGTGG--TTGAAGATAAAC-GTAA 5’(597-625)**

**||||:|:||||: |||||:||:|:||| ||| #||||:||||||||||**

**_12_TTTAAGAGTAAAT-GTAATGAAGTGAAT---TAT---GTGGTAACATTAAGAATATATA 5’ pND9(609-644)**

**_12_TTTAAAAGTGAAT-GTAATAAGATAGAT---TG----ATAG 5’ bsND9(609-640)**

**:||||: |||:|:|:||||:| ||| |||||::||||||:||**

**_12_TGTAAAT-GTAGTGAGATAAGT---TAT--AGTGGTGACATTAGGAAATATATA 5’ pND9(615-659)**

**_12_TTAAAGTTAGT-GTAATAAGATAAGT---TAT--AGTGGCAACATTAAGTATATA 5’ bsND9(618-658)**

K) Ribosomal Protein S12

**0 10 20 30 40 50 60 70 80 90**

**cUaaUacacUUUUgaUaacaaacUaaagUaaauauauuuuguuuuuuuugcguauguga*UUUUUGuAuG*GuuGuuguuuac*guuuuguuuuauuugu**

**||:|::::||:|:|||:|||||:| :||:||||:| |||||| |::|**

**pRPS12(35-76) _12_TATTTAGAGTGGAAGAGACGTATACATT-GAAGACATGC-CAACAAATA (96-121)_12_TATTATAGTA**

**bsRPS12(38-78) _18_TAGTGAAGAGAGTGTATATGCT-AAAGACATAC-CAACAATATATA(96-121)_11_TAATAGTA**

**|:||:|:||||::||| |:||::|||| |||||||| (96-131)_10_TATAGTA**

**pRPS12(43-78) _14_TATATAGTTAGAAGATGCATGTACT-AGAAGTATAC-CAACAACATATA 5’**

**bsRPS12(43-78) _12_TATATAGTTAGAAGATGCATGTACT-AGAAGTATAC-CAACAACATATA 5’**

**|::||:: :||::|:|:||| ::|:|||:||||||||**

**pRPS12(63-109) _12_TATAGTATGT-TAATGATAGATG-TGAGACAGAATAAACA**

**bsRPS12(66-99) _07_TAGTAGAGTGT-CAATAGTAAATG-CAAAACAAAATAAATATA5’**

**:|::|:||| |||:|:||:|||||||**

**pRPS12(74-106) _12_TAATATGTCATTAGTAGATG-CAAGATAAGATAAACA**

**bsRPS12(73-115) _16_TCTTTTATAGTAAATG-TAGAGCAAGATAGACA**

**100 110 120 130 140 150 160 170 180 190**

**uuuauguuauuauaugaguccg**cgauugcccaguuccgguaaccgacguguauuguaugc**c****guauuuuauuUauauaauuuuguuuggaugu**

**|||||:|||| :|||:|:|:|||||:|||:||::|:||||:|**

**AAATATAATATA 5’(63-109) pRPS12(169-208) _12_TATATAGAGTGAATATGTTAGAATGAGCCTATA**

**|||||:| bsRPS12(156-207) _12_TTATATG--G----TATAAGATAGATGTGTTAGAATAGACTTACA**

**AAATATATATA 5’ (74-106)**

**GAATACAATAATATATATA 5’(73-115)**

**||||:||||:|||||:|:|||| ::||:|**

**AAATGCAATGATATATTTAGGC--ACTAA 5’ (96-121) pRPS12(194-235) _11_TTTTATA**

**GAATGTAGTGATATATTCAGGT--AGCTAACGTGTCAAATATA 5’(96-121) bsRPS12(194-235) _09_TTTTATa**

**||||:||||:|||||:|:|||| ||||||||#|:|||**

**AAATGCAATGATATATTTAGGC--GCTAACGGATTAAGATATA 5’(96-131)**

**|: |:||::||#|::||||:|||||:||||||||||||**

**_10_TATTCAGT--GTTAGTGGATTGAGGCTATTGGTTGCACATAACATTCA 5’ (119-158)**

**:|||:|#||:|:|#:|||||||||||:||:|:||||| | ||**

**TTTTAATGTGTTAGGATCATTGGCTGCATATGATATACG--G----CAATATA 5’ pRPS12(139-170)**

**_11_TTTAGTAGATTGAGGCTATTGGTTGCACATAACATTCATA 5’ bsRPS12(133-158)**

**200 210 220 230 240 250 260 270 280 290**

**ugcguuguuuuuuuuguuguuuuauugguuuaguuaug**Ucauuauuuauuauaga***gggUggugguuuuguugauuuaccc***g****gug*Uaa**

**||||||||| ::|||:::||||:||#|| : ||: |||**

**ACGCAACAATAAATA 5’ (169-208) pRPS12(267-322) _07_TTTAAAGTGACTAGATAGG---T----CAT-ATT**

**ATGCAACATATA 5’(156-207) bsRPS12(288-322) _12_TTTAAAGTAACTAGATGGA---T----CAT-ATT**

**bsRPS12(269-308) _10_TTAGTACAAGAGCAGTTAAATGGG---C----TAC-ATT**

**|||: ||||:|:|||:||:||| :::|||::|||||||||||:|**

**_14_TATAT--AGTAGTGAATGATGTCT---TTTACCGTCAAAACAACTAGAATATA 5’pRPS12(234-280)**

**_15_TATAT--AGTAGTGAGTGATATCT---TTTACCGTCAAAACAACTAGAATATA 5’bsRPS12(234-280)**

**|:|:|::|:|:||:|::||:|||||::|||||||||#| |||||||**

**ATGTAGTAGAGAAGATGACGAAATAGTCAAATCAAT-C--AGTAATATATA 5’(194-235)**

**ATGTAGCAGAGAAGATGACGAAATAGTCAAATCAAT-C--AGTAATATATA 5’(198-235)**

**:||:|:|:|:|::|||::|:|||:|:|:|||||:| |||||||**

**_14_TaTAATAGAGAGagTAACGGAGTAATCGAGTCAATGC--AGTAATATAtaTA 5’ pRPS12(203-246)**

**_07_TATAATAGAGAGAGTAATGGGATAGTTAAATCAATAC--AGTAATTAAAATA 5’ bsRPS12(203-245)**

**300 310 320 330 340 350**

**aguauuauaca*cg**Uauuguaaguuaga*UUUagauaUaagaUaUgUUUUU[aaua]polyA**

**|:|||:||||| || |||||||**

**TTATAGTATGT-GC--ATAACATATA 5’ (267-322)**

**TCATAATATATA 5’(269-308)**

**TTATAATGTGT-GC--ATAACATATA 5’bsRPS12(288-322)**

**|| |: ||||::|||||||| ||:||||||||||||||||**

**_16_TAAGT-GT--ATAATGTTCAATCT-AAGTCTATATTCTATACAATATAAA 5’ pRPS12(309-349)**

**_24_TAAGT-GT--GTAATGTTCAATCT-AAATCT-TAGTCTATACAAAATAAA 5’bsRPS12(309-336)**
