# Supplementary material for: Analysis of the Trypanosoma brucei EATRO 164 Bloodstream Guide RNA Transcriptome
Source: PLoS Negl Trop Dis. 2016 Jul 11;10(7):e0004793. doi: 10.1371/journal.pntd.0004793 (PMC4939953; doi:10.1371/journal.pntd.0004793)
Supplement: S1 Table — Populations of gRNAs are bordered boxes. A) ATPase 6; B) Cytochrome Oxidase III; C) C-Rich Region 3; D) C- Rich Region 4; E) Cytochrome b; F) Maxicircle Unidentified Reading Frame II (Murf II); G) NADH Dehydrogenase Subunit 3; H) NADH Dehydrogenase Subunit 7; I) NADH Dehydrogenase Subunit 8; J) NADH Dehydrogenase Subunit 9; K) Ribosomal Protein S12. (DOCX) [file pntd.0004793.s003.docx]

Supplementary Table 1. All gRNA major classes identified in the EATRO 164 procyclic (shaded gray) and bloodstream (white) transcriptomes. Populations of gRNAs are bordered boxes. A) ATPase 6; B) Cytochrome Oxidase III; C) C-Rich Region 3; D) C- Rich Region 4; E) Cytochrome b; F) Maxicircle Unidentified Reading Frame II (Murf II); G) NADH Dehydrogenase Subunit 3; H) NADH Dehydrogenase Subunit 7; I) NADH Dehydrogenase Subunit 8; J) NADH Dehydrogenase Subunit 9; K) Ribosomal Protein S12.

A) ATPase 6

| 5' | 3' | Reads | ATPase 6 gRNA Sequences |
| --- | --- | --- | --- |
| 31 | 75 | 2,044 | AT ATAAACGTAACTGAAATGAATCACGAGAGAAAGATAAAGATATAT ATTTTTTTTTTTT |
| 29 | 72 | 1,630 | ATATAC AACGCAACCAGAGTAAATCATGAAGGGAAAGTGAAGGCATATTT TTTTTTTTTTT |
| 31 | 75 | 143 | AT ATAAACGTAACTGAAATGAATCGCGAGAGAAAGATAAAGATATAT ATTTTTGTTTTTTTTTTTTTTT |
| 31 | 75 | 489 | AT ATAAACGTAACCAAAATGGATCATGGAAGAGAAGTAAAGATATGT ATTTTTTTTT |
| 29 | 75 | 395 | AT ATAAACGTAACCAAAATGGATCATGGAAGAGAAGTAAAGATATGTTT TTTTTTTTTTT |
| 35 | 75 | 224 | AT ATAAACGTAACCAAAATGGATCATGGAAGAGAAGTAAAGAT TTTTTTTTTTTTT |
| 29 | 62 | 203 | ATATACAACGCAACC AGATAAATCATGAAGAGAAAGTGAAGGTATATTT TTTTTTTTT |
| 33 | 75 | 144 | AT ATAAACGTAACCAAAATGGATCATGGAAGAGAAGTAAAGATAT TTTTTTGTTTTT* |
| 31 | 62 | 95 | AACGCAACC AGATAAATCATGAAGAGAAAGTGAAGGTATAT ATTTTTTTTTTTTTT |
| 39 | 75 | 82 | AT ATAAACGTAACCAAAATGGATCATGGAAGAGAAGTAA TTTTTTTTTTTTTTTT |
| 37 | 75 | 40 | AT ATAAACGTAACCAAAATGGATCATGGAAGAGAAGTAAAG TCAACTTAATTTTTTTT* |
|  |  |  |  |
| 62 | 102 | 1,435 | ATACA ATCATACACAGTAGTACATATATAGTGATAGACGTGATTAA TTTTTTTTTTT |
| 62 | 100 | 154 | ATAAAA CATACACAATGATATATACATAGTAATAGATGTGATTAA TTTTTTTTTATTTTTTTTTTTTT |
| 62 | 102 | 92 | ATATAA ATCATACACGATAATATATGCGTAGTAACAGATGTGATTAA TTTCTTTTTTTTTTTTTT |
| 74 | 102 | 10 | ATATAT ATCATACACGATAATGCATATGTAGTAAC TTTTTTTTTTTTT |
| 64 | 100 | 9 | ATAAAA CATACACAATGATATATACATAGTAATAGATGTGATT TTCTTTTTTTTTTT* |
|  |  |  |  |
| 86 | 127 | 2,158 | ATAT AAATACACAGTAGAATATGATCTAGGTTATGTATGATGATAT TTTTTTTTTTT |
| 90 | 129 | 177 | ATATA TAAAATACACGATAGAGCATAACTTAGATTGTATATGATA TTTCATTTTTTTTTTTTTTT |
| 84 | 118 | 85 | ATAATAATACAC ATAGAACATGACCTAGATTGTACATAGTGATATAT TTTTTTTTTTTT |
| 82 | 118 | 38 | ATATAATAATACAC ATAGAACATGACCTAGATTGTACATAGTGATATATAT TTTTTTTTTTTTT |
| 86 | 118 | 28 | ATATAATAATACAC ATAGAACATGACCTAGATTGTACATAGTGATAT TTTTTTTTTTTTTTT |
| 83 | 118 | 22 | ATAATAATACAC ATAGAACATGACCTAGATTGTACATAGTGATATATA ATTTTTTTTTTTTTTTTTT |
| 91 | 129 | 21 | ATATA TAAAATACACGATAGAGCATAACTTAGATTGTATATGAT TTTTTTTTCTTTTTTTTT |
|  |  |  |  |
| 113 | 152 | 743 | ATAC ATCAAAAATCAACGTTAGACAGTTAAGATATGTGATAGAA GATAATTTTTTTTTTTT |
| 105 | 152 | 54 | AC ATCAAAAATCGACATTAGATAATTGAGGTATGTGATAGAGTATAATTT TTTTTGTTTTT |
| 105 | 148 | 128 | ATATC AAAATCAACATTGAGCAGTTAAAGTACGTGGTAAGATATAATTT TCTTTTTTTTTT |
| 116 | 152 | 84 | AC ATCAAAAATCAACATTGAGCAATTGAGGTACATGATA TGATATAATTTTTTTTT |
| 104 | 148 | 16 | ATATC AAAATCAACATTGAGCAGTTAAAGTACGTGGTAAGATATAATTTA TTTTTTT* |
|  |  |  |  |
| 138 | 183 | 430 | AT ATACAAATCAAACAGACAGAGTAATAGAAGGTTGAAGATTGATAT AGTTTTTTTTTTT |
| 144 | 177 | 210 | ATATC ATCAAACAAACAGAATAATAGAGAATCAGAGGT GAATGTTAAGTTTTTTTTTTTTTTT |
| 139 | 175 | 3,468 | ATAAA TAAACAAACAAAATGATAAAAGGTCAGAGATTGATG GTGAATAATTTTTTTT* |
| 132 | 177 | 205 | ATAT ATCAAACAAACAAAGTAATAGAAAGTCAGAGATTGATGTTAAATA TTTTTTTTTT |
|  |  |  |  |
| 164 | 208 | 54 | ATAT ATAAACACAAATCAACGAATAGATATAAGTCAGATAGATGG TGTATTATTTTTTTTTTTTAAAAAAAAAAA |
| 176 | 210 | 36 | AT AAACAAACACAAATCAGTAGACGAGTACAAGT GAGATGGACGTATAGATTTTTTT |
| 165 | 208 | 24 | ATAAT ACAAACACAAACTGATAGACGAATACGAGTTAGATGGACG TATTTTTT |
| 158 | 208 | 14 | ATAT ACAAACACAAACTGACGAATAGATACAGATTAAGTGAATGAAATAAT TTTTTTTTTTT |
| 164 | 208 | 218 | AT ATAAGCACAAACCAATAGACAGATATAAGTCAGATAGATGA TTATTTTTTTTTTTTTT |
|  |  |  |  |
| 192 | 243 | 172 | ATATAAATTAAACAACATAGATTACAGTGATAGAAGTAAATGTGAATTA TTTT |
| 218 | 248 | 147 | ATC AGACTATGTGAGTTAGATGACGTGAATTATA CTGTATATTTTTTTTTTTT |
| 190 | 243 | 52 | ATATAAATTAAACAACATGAACTATGATGATAAAGGTAAATGTGAATTAAT TTTTTTTTTTTTTTTTTTT |
| 189 | 243 | 10 | ATATAAATTAAACAACATGAACTATGATGATAAAGGTAAATGTGAATTAATG TACTATGATAACTT* |
| 207 | 243 | 7 | ATATAAATTAAACAACATGAACTATGATGATAAAGGT TTTTTTTTTT |
| 193 | 246 | 5 | *ATTGTATAAATTAAACAACATGAACTATGATGATAAAGGTAAATGTGAATT TTT |
|  |  |  |  |
| 224 | 269 | 864 | ACATAA TAATACAATAATACGAGATTAGACTATGTGAATTAAATGATATGA TTTTTTTTTTTG |
| 226 | 269 | 808 | ACATAA TAATACAATAATACGAGATTAGACTATGTGAATTAAATGATAT TTTTTTTTGTTTT |
| 221 | 262 | 149 | ATATAT ATAATACAAAATTGAACTGTATAAGTTAGACAATGTGAATT TT |
| 219 | 262 | 105 | ATATAT ATAATACAAAATTGAACTGTATAAGTTAGACAATGTGAATTAT TTTTTTCTTTTTTT |
| 218 | 262 | 57 | ATATAT ATAATACAAAATTGAACTGTATAAGTTAGACAATGTGAATTATA TTTTTTTTTTTTTTTTTTTTT |
| 221 | 267 | 32 | AC ATACAATAATACAGAATTAAACTGTGTAAGTTAGATAGTGTAAATT TTTTTTTTTT* |
|  |  |  |  |
|  |  |  |  |

| 5' | 3' | Reads | ATPase 6 gRNA Sequences cont. |
| --- | --- | --- | --- |
| 248 | 292 | 25,157 | ATATA AAATACAAATTCGAGTAGGTAGTACAATGATATGAGATTA TTTTTTTTTTTTT |
| 253 | 298 | 5,405 | ATATAT AACAACAAATATAGATTCAAGTAAGTGATGTAGTAATATGA TTTTTTTTTTT |
| 255 | 292 | 244 | ATATA AAATACAAATTCGAGTAGGTAGTACAATGATAT TATTATTAATTTTTTTTTTTTTTT |
| 252 | 292 | 134 | ATATA AAATACAAATTCGAGTAGGTAGTACAATGATATAGA TTATTAATTTTTTT |
| 262 | 304 | 4,881 | ATAT ATACAAAACAACAGATATAGATTCGGATAGGTAATATGA GATCTTTTTTTTTTTTT |
| 259 | 304 | 395 | AT ATATAAAACAACAGATATGAATTCAAGTGAGTGATACAGTA TATTTTTTTTTTTTTTTATA |
| 254 | 298 | 290 | ATATAT AACAACAAATACGAATTCAGATAGGTAGTATGATGATATA TTTTTTTTTTTTTTTGT |
|  |  |  |  |
| 266 | 313 | 586 | AAAAAA AAAAAAACAATACAAGATGACAGGTATAAGTTTGGATGAGTAAT TTTTTTTTTTTTG |
| 281 | 313 | 14 | AAA AAAAAAACAATACAGAATAGTAGGTATAGATT AGATATGTGATTTTTTTTT* |
| 284 | 310 | 13 | ATAT AGAACAATACAAAATAACGAGTACAG TTTGTTTT |
| 283 | 310 | 2 | TAT AGAACAATACAAAATAACGAGTACAGG ATAAGTGATATTTTTTTT |
| 281 | 312 | 2 | AT AGAAAACAATACAGAATAGTAGGTATAGATT AGATATGTGATTTTTTTTTTTTTTTTTTT |
|  |  |  |  |
| 291 | 329 | 133 | AAATATAT AAATGCAATATACGATAGAGAAATGATATAAGATGATAA TTTTTTTGTTTTTTTT* |
| 293 | 329 | 12 | AAATATAT AAATGCAATATACGATAGAGAAATGATATAAGATGAT TTTTTTTTTTTTTT |
|  |  |  |  |
| 301 | 345 | 647 | ATAT AACAAAACAAAAGTAGAAGTGCAGTATATGATAGAAAAATGATGT CAAATTTTTTTTTTT |
| 300 | 346 | 263 | ATAT AAACAAAACAGAAATAGAAATGCAATATACGATAAGAAAATGGTATA TTTTTTTTTTTT |
| 301 | 335 | 125 | ATAT ACAAAACAT AAATAAAAGTGCAGTATATGATAAAGAGATAATAT TTTTTTTTTTT |
| 301 | 345 | 33 | ATAT AACAAAACAAAAGTAAAAGTGCAGTGTATGATAGAAAAATGATGT CAAATTTTTTTTTTTTTTT |
|  |  |  |  |
| 331 | 375 | 24,736 | ATAT AATTATTAAACAAGAGAAAGTCACGTAAAAGGTAGAATGAAGATA TTTTTCTTTTTT |
| 332 | 378 | 8,776 | AT ATAAATTATTAAACAGAAAGAGATCATGTAGAAAGTGAGATAGAAAT TTTTTTTTTTTTCT |
| 331 | 371 | 3,561 | ATATAA ATTAAACAAAAAGAAATCACGTAGAAGACAGAATAGAGATA TTTTTTTTTTTTG |
| 331 | 375 | 712 | ATAT AATTATTAAACAAGAGAAAGTCACGTAAAAAGTAGAATGAAGATA TTATTTTT |
| 332 | 378 | 387 | AT ATAAATTATTAAACAGAAAAGAGTCATATAGAAAATAAGATAGAAAT TTTTTTTTTTTT |
| 331 | 374 | 302 | ATAT ATTATTAAACAAAGAGAAATCATATAAGAGACAGAATGAGAATA TTTTTTTTTATTTTT |
| 332 | 378 | 144 | AT ATAAATTATTAAACAGAAAGAGATCATGTAGAAAGTGAGATAAAAAT TTT |
| 331 | 371 | 1,547 | ATATAA ATTAAACAAAAAGAAATCACGTAGAAGACAGAATAGAGATA TTTTTTTTTTTCTTT* |
|  |  |  |  |
| 360 | 407 | 181 | ATATAT ACATCCATAAAATTATCATCAGTTAATAGATTGTTAAATGAAAA TTTT |
| 349 | 389 | 41 | ATATAA ATCACCAACTAATAAGTTATTGAATGAGAGAAAGTTATATA TTTTTTTTTTTT |
| 352 | 401 | 705 | ATATA ATAAAACTATCACTAACTAATGGATTGTTAAGTAGAAGAGAATCAT TTTTTTTTTTT |
| 360 | 407 | 429 | ATATAT ACATCCATAAAATTATCATCGGTTAATAGATTGTTAAATGAAAA TTTTTTTTTTT |
| 354 | 401 | 160 | ATATA ATAAAACTATCACTAACTAATGGATTGTTAAGTAGAAGAGAATC CTTTTTTT |
| 362 | 407 | 143 | ATATAT ACATCCATAAAATTATCATCGGTTAATAGATTGTTAAATGAA TTTTTTTTTTTT |
| 349 | 389 | 104 | ATATAA ATCACCAACTAATAAGTTATTGAATGAGAGAAAGTTATATA TTTTTTTTT |
| 361 | 407 | 74 | ATATAT ACATCCATAAAATTATCATCGGTTAATAGATTGTTAAATGAAA TTTTTTTTTTTTTTT |
|  |  |  |  |
| 387 | 435 | 1,428 | ATATAT AACACAACAAGAAACGAATGAGAGAAGTATCTATGAGATTATT TTTTTTTTTCGTTT* |
| 387 | 435 | 1,049 | ATATAT AACACAACAAGAGACGAATAGAAAAGATATCTGTGAAATTATT TTTTTTTTTTATT |
| 387 | 437 | 934 | ATATAT AAAACACAATAGAAAACGGATAAGAGAGATATTCATAGAGTTATT TTTTTTTTTGTTT |
| 387 | 435 | 664 | ATATAT AACACAACAAGAGACGAATAGAAAAGATATCTGTGAAATTATT TTTTTCTTTTTT |
| 390 | 435 | 635 | ATATAT AACACAACAAGAGACGAATAGAAAAGATATCTGTGAAATT TTTTTTTTTTT |
| 398 | 435 | 28 | ATATAT AACACAACAAGAGACGAATAGAAAAGATATCTGTGA TTTTT |
| 397 | 435 | 23 | ATATAT AACACAACAAGAGACGAATAGAAAAGATATCTGTGAA TTTTTTTTTTTTT |
|  |  |  |  |
| 424 | 464 | 25,624 | ATAT ATGACACAACGAGGGAAGATACTCTAAAGGACACAGTGAAA TTTTTTTTTTTT |
| 427 | 467 | 2,307 | ATAT ATAACGACACAATAGAGAAAGATGCTCTGAGAGATGTAATA TTTTTTTTTTTTG |
| 421 | 460 | 1,864 | ATAAAT TACAACAAAGAAAGATACTCTAGAAAGCACAGTGAGAAAT TTTTTTTTCTTTTTTT |
| 424 | 457 | 368 | AAATTAACGACA AACAAAGAGAAATACTCTGAGAAATATGATGAAA TTTTTTTTTTTT |
| 424 | 464 | 6,879 | ATAT ATGACACAACGAGGGAAGATACTCTAAAAGGTACAGCGAAA TTTTTTTTTTTTT* |
| 427 | 468 | 1,781 | ATAT AACAACGATACGACAGAGAAAGATATTCTAAGAGATATGACA TTTTTTTTTTTTT* |
| 424 | 457 | 232 | AAATTAACGACA AACAAAGAGAAATACTCTGAGAAATATGATGAAA TTTTTTTTTTTT |
|  |  |  |  |
| 455 | 491 | 368 | ATATATAATTAC AAACAAACGCAGAGATGTCGGTAAATAATGATATAAT TTTTTTTTTTT |
| 455 | 497 | 22 | ATAT ATTACAAAACAGACGTAAAGATGTCGATGAATGGTGGTATAAT TTTTTTTTTTTTTT |
| 452 | 477 | 54 | AATT ACGTCGATAGATAACGATACAATGAG ATTAATTTT |
| 476 | 500 | 14 | AAATT TAAATTACAAGACAAACGTAGAAGC TTTTTTTTTTTTTTTTTTTTTTTT |
| 458 | 500 | 1 | AAATT TAAATTACAAGACAAACGTAGAAGCGTCGATAGATAATGATATTTTTTTTTTTTTTT |
|  |  |  |  |
| 487 | 526 | 8,723 | ATACAA ATCAACAATAGAAGATGGGATGATAATAGATTGTGAGATA TTTTTTTTTTTTTTTTG |
| 487 | 528 | 1 | ATAC ACATCAACAATAGAAGATGGGATGATAATAGATTGTGAGATA TTTTTTTTTTTTTTTTTTTTTTTTTTT |
| 487 | 526 | 635 | ATACAA ATCAACAATAGAAGATGGGATGATAATAGATTGTGAGATA TTTTTTTTTCTTTTTT |
|  |  |  |  |
| 521 | 567 | 232 | AA AAAAAAAAAAAACAAAAATAGAATAAAGAAAGTCAGAGAATGTTAAT TTTTT |
| 520 | 553 | 85 | AAAAAAAAAAAC AAAATAAAGTAAGAAGAATCAGAGAGTGTCAATA TATTTT |
| 521 | 553 | 11 | AAAAAAAAAAC AAAATAAAGTAAGAAGAATCAGAGAGTGTCAAT TTTTTTTTTTTT |
|  |  |  |  |
|  |  |  |  |
|  |  |  |  |
| 5' | 3' | Reads | ATPase 6 gRNA Sequences cont. |
| 557 | 593 | 69,619 | AATAAATCGATAACAAAGAACACTGTAAAAGAGAGAA TGAGAGTAAATATTTTTTTTT |
| 549 | 593 | 2,587 | AC AATAAATCAATAACAGAGAATATCATAGAGAGGAAAGATAGAAAT TTTTTTTTTTTTGTTTGTACTT |
| 549 | 592 | 181 | ATAT ATAAATCAATGACAAGAAGCACTGTAGAAAAAGAGAGTGAAAAT TTTTATTTTTTTT |
| 546 | 592 | 313 | AT ATAAATCAATAACAGAAGATGCCATAGAGAGAGAAAGTGAGAGTAAA TTTTTTTTTCT |
| 546 | 593 | 76 | AT AATAAATCAATAACAAAGAACATTGTAAAAGAGAAAAGTGAGAATAAA TTTTTTTTTTTTT |
| 549 | 592 | 30 | ATAT ATAAATCAATGACAAGAAGCACTGTAGAAAAAGAGAGTGAAAAT TTTTGTTTTTTCT |
|  |  |  |  |
| 568 | 611 | 670 | ATACT AAACACAAAAATGAATAAAATAAGTCAGTGATAGAAGATATTAT TTTTTTTTTTTT |
| 576 | 616 | 115 | AT AAACAAAACACAAAAATAAGTAAAGTAGGTCAGTGATAAGA TATACATTTTTTT* |
|  |  |  |  |
| 589 | 629 | 854 | AT AAATAATAAACAGAAACGGAATACGAGAATAAGTAAAGTGA TTTAATTTTTTTTTTTTT |
| 589 | 629 | 3,292 | AT AAATAATAAACAGAAACGGAATACGAGAATAAGTAAAGTGA TTTAATTTTTTTTTT |
|  |  |  |  |
| 613 | 654 | 618 | ATATAT AATCCAACAGATATAAGAGCATGTAAAATAGTAAGTGAAAAT TTTTTTTTTTAT |
| 613 | 657 | 183 | AT ATAAATCCAACAAGTATAAGAACATATAGAATAGTAGGTGAAAAT TTTTTTTCTTTT |
| 613 | 654 | 459 | ATAT AATCCAACAGATATAAGAGCATGTAAAATAGTAAGTGAAAAT TTTTTTTTTTT |
| 613 | 657 | 399 | ATAT ATAAATCCAACAAGTATGAAGACACGTAAAATAGTAAATGAAAAT TTTTTTTTTTTTTT* |
|  |  |  |  |
| 640 | 689 | 39,063 | ATAT ATAAATAACTGTAGTATGGTGGTAGATGAGTTTGATAGATATA TTTTTTTTTTTT |
| 647 | 689 | 678 | ATAT ATAAATAACTGTAGTATGGTGGTAGATGAGTTTGAT TTTTTTTTTTT |
| 640 | 689 | 131 | ATAT ATAAATAACTGTAGTATGGCGGTAGATGAGTTTGATAGATATA TTTTTTTTTTT |
| 654 | 689 | 234 | ATAT ATAAATAACTGTAGTATGGTGGTAGATGA TTTTGATAGATATATTTTTTTTTTTT |
| 643 | 667 | 4,401 | ACATATATAAATAACTGTGATATTGCGGTAGATGGATCTGATGAAT TTTTTTTTTTTTTT† |
| 640 | 668 | 250 | ATATAATAAATAACTATAATAAGGTGGTAAGTGAGTTCAGTGAATATA TTTTTTTTTATTTT† |
| 640 | 662 | 165 | AAAACTGTAATATGGAGGTAAGTGAATTTGATAGATGTA TTAATTTT† |
|  |  |  |  |
| 680 | 714 | 7,581 | AAAATA CAACTGCAAGATCGTGTTATAGAGGATAAGTGATT TAATTTTTTTTTTTTT |
| 680 | 719 | 1,291 | ATATAA ATTATCAACTGTGAGATTATATTACAAGGAATAAGTGATT TTTTTTTTTTTAT |
| 672 | 716 | 119 | ACACA ATCAACTGCAGAATTATATTACAGAGAGTGAGTAATTGTAA AATTTTTTTTTTTT |
| 680 | 714 | 105 | AAATA CAACTGCAAGATCGTGTTGTAGAGGATAAGTGATT TAATTTTTTTTTTT |
| 671 | 714 | 319 | AGATA CAACTGCAAGATCATATTATAAGAGGTGAATGATTGTAAT TTTTTTTTTTTTTT* |
| 685 | 714 | 309 | ATATA TAACTGCAGAATCATATTATAAGGGATGAA CGATTGTTTTTTTTTTTTT |
|  |  |  |  |
| 698 | 728 | 740 | ATT AAAATCCATTATCGATTGTAGAGTTATGT GATAGAGAATAATTTTTTTTTTT |
| 686 | 728 | 12 | ATATATT AAAATCCATTATCGATTGTAGAGTTATGTTATAGAGAATAA TATTTTTTTTTTTTTTTTTTTTT |
| 699 | 727 | 88 | AAATCCATTATCAGTTGCGAGATTGTA GTATAAAGAATAATTTTTTTTTTTTTT |
| 699 | 727 | 24 | ATATAT AAATCCATTATTAACTGTAAGATTGTA GTATAGTTTTTTTTTTTTTTTTT |
|  |  |  |  |
| 720 | 767 | 4,588 | AT AAATCAAATACAGAACTGAATAGACGATAAAGATAGTGAGAAATTT TTTTTTTTTTG |
| 715 | 755 | 2,272 | ATATATAT AAACTAAACAAATAGCAGAGACAGTGAGAGATTCGTTAT AATTTTTTTTTTTTT |
| 728 | 765 | 920 | AT ATCAAATACAAAACTGAGCAGATGACAGAGATAGTAAA TGATTTATTTTTTTTTTTG |
| 720 | 767 | 165 | AT AAATCAAATACAGAACTAGATGAACAATAGAGATAGTGAGAAATTT TTTTTCTTTTTT |
| 717 | 763 | 1,613 | ATATA TAAATACAAAACTAGATGAATGACAGAAACGATGAGAGATTTATT TTTTTTTTTTTTTT* |
| 718 | 763 | 488 | ATA TAAATACAAAACTAGATGAATGACAGAAACGATGAGAGATTTAT AATTTTTTTTCTTTTTTTT |
| 720 | 763 | 452 | ATATA TAAATACAAAACTAGATGAATGACAGAAACGATGAGAGATTT TTTTTTCTTTTT |
| 720 | 767 | 269 | AT AAATCAAATACAGAACTAGATAGACGATAGAGATAGTGAGAAATTC TTTT* |
| 720 | 767 | 177 | AT AAATCAAATACAGAACTAGATAGACGATAGAGATAGTGAGAAATTT TTTTTTTTTATCTTTCT |
|  |  |  |  |
| 747 | 789 | 13 | ATAAAT ACAACAATATAATAACTGTCGAAGGTTGAATATGAGATTAAAT TTTTTTTTTTT |
| 747 | 789 | 587 | ATATAT ACAACAATATAATAGCTATCAGAGGTTGAATGTGAGATTAAAT TTTTTTTTTTTGG |
| 745 | 789 | 91 | ATATAT ACAACAATATAATAGCTATCAGAGGTTGAATGTGAGATTAAATGA TTTTTTTTTTT |
|  |  |  |  |
| 790 | 822 | 8,663 | ATA CTATAACTCCAATGACGAAATCAGTTTTA CAGTGATATGATAATT TTTTTTTTTTTT |
| 770 | 822 | 1 | GGA CTATAACTCCGATAACGAATCAGATTTTGACAGTGATATGATAATTATT TCCCTTTCTTCTC |
| 773 | 822 | 428 | ATATA CTATAACTCCGATAACGAATCAGATTTTGACAGTGATATGATAATT TTTTTTTTT |
| 774 | 822 | 223 | ATATA CTATAACTCCGATAACGAATCAGATTTTGACAGTGATATGATAAT |
| 777 | 822 | 195 | ATATA CTATAACTCCGATAACGAATCAGATTTTGACAGTGATATGAT TTTTTTTTTTTTTT |

B) Cytochrome Oxidase III

| 5' | 3' | Reads | COIII gRNA Sequences |
| --- | --- | --- | --- |
| 35 | 70 | 1,179 | ATAATT AATATACAACGAGATAGAGACGTAAAAGAAT TGATGTATTTTTTTTTTTG |
| 36 | 73 | 826 | AT ATAAATATACAACGAGATGAAGGCATAGAGAAA AGATGGTATATAATTTTTTTTTTTTTT |
| 29 | 70 | 112 | ATATAC AATATACAACGGAATGAGAATATAAGAAAGTGATGATA TTATTTTTTTTTTT |
| 36 | 70 | 14,200 | ATATAT AATATACAACGAGATAAGAACATAGAGAAA AGATGGTATATATTTTTTTTTTTTG |
|  |  |  |  |
| 54 | 101 | 1,386 | ATAT AAAACAAAAACATCACTGATATTGACGGATATATGATGA TAAATTTTTTTTTTTG |
| 51 | 99 | 721 | ATATAT AACAAAAACACTACTAGCGTTGACAGATATATGATGAAAT TTTTTTTTTTTG |
| 51 | 99 | 364 | ATAT AACAAAAACACTACTAGCATTGACAAATATATGATGAAAT TTTTTTTTCTTTT* |
| 52 | 101 | 229 | AT AAAACAAAAACACTGCTAATATCGACGAATATATGATGGAA AATTTTTTTTTTTATT* |
| 50 | 92 | 122 | ATATAAAAT ACACCACTGATATCAACGAGTATATGATGAGATA TTTTTTTTTTTTTT |
| 49 | 95 | 78 | ATATAT AAAACACCACTGACATCGATAAGTATATAGTGAAGTGA TTAATTTTTTTTTTTTTTT |
|  |  |  |  |
| 81 | 112 | 834 | GTA GAGTGAAGATAGAGAAATAAAGATATCGTT TTTTTTTTTTTTT |
| 81 | 116 | 550 | ATATATAATAACAATA GCAGGTAAAGGTGAGAAAGTGAAGATATCATT TTTTTTTTTT |
| 81 | 131 | 1 | TACATAATAACAGTGGCGGGTAGAGATAGAAGAATAAAGATACTATT TTTTTTTT |
| 88 | 115 | 443 | AACGATGGA TAGGTAGAGATAGAGAAATGAAGATATT TTATTTTT* |
| 88 | 115 | 117 | AATAACAATGGA TAGGTAGAGATAAAGAAATGAAGATATC TTTTTT |
| 88 | 115 | 96 | AATAACGATGGA TAGGTAGAGATAGAGAAATGAAGATATC TTTTTTT |
| 81 | 112 | 64 | ATACATAACAGTGGCAGA GTGAAGATAGAGAAATAAAGATATCATT TTTTTTTTTTT* |
|  |  |  |  |
| 108 | 132 | 6 | AT ATATACAATAACAGTGGTAGGTAGA TTTTTTTTT |
|  |  |  |  |
| 117 | 156 | 104 | ATATATA TCCAACAAACAGAGTAACCGATACATAGTGATAGTG ATATTTTTTTTTTTTT |
| 117 | 155 | 465 | ACATATA CCAACAAACAGAATAACTAGTGCACAGTGATGATG ATAGTTTTTTTTTTTTTTTT |
| 118 | 150 | 36 | ATATA CAACAAACAAAATAATCGATGCACAGTGATAGT AGTAGTTTTTTTTTTTTT |
|  |  |  |  |
| 141 | 185 | 126,513 | ATAT ATTACCAAACAATAGACGAGTAGATTCTAATAGATGA TTTAATTTTTTTTTTTTG |
| 141 | 185 | 761 | ATAT ATTACCAAACAATAGATGAGTAGATTCTAATAGATGA TTTAATTAAGTTTT* |
| 134 | 188 | 542 | ATAT AAAACTACCAAACAGTAAATAGATAAGTTCTAATAAGTGAGATAATT TTTTTTTTGTT |
| 146 | 185 | 350 | ATAT ATTACCAAACAATAGACGAGTAGATTCTAATA TATGATTTAATTTTTTTCTTTT |
| 142 | 185 | 199 | ATAT ATTACCAAACAATAGACGAGTAGATTCTAATAGATA TTTAATTATTTTT |
| 141 | 185 | 183 | ATAT ATTACCAAACAATAGACAAGTAGATTCTAATAGATGA TTTAATTTTTTTTATTTT |
| 145 | 185 | 172 | ATAT ATTACCAAACAATAGACGAGTAGATTCTAATAG CTGATTTAATTTTGTTTTTTTT |
| 143 | 185 | 116 | ATAT ATTACCAAACAATAGACGAGTAGATTCTAATAGAT CATTTAATTATTTTT |
| 131 | 188 | 109 | ATAT AAAACTACCAAACAGTAAATAGATAAGTTCTAATAAGTGAGATAATTAAT TGTTATTTTTTTTTTTTTATC |
| 147 | 185 | 106 | ATAT ATTACCAAACAATAGACGAGTAGATTCTAAT CGATGATTTAATTAATTTTTTTTTTTTTTTTT |
| 141 | 185 | 99 | ATAT ATTACCAAACAATAGACGAGTAGATTCTAATAAATGA TTTAATTAATTTTTTTT |
| 141 | 185 | 62,901 | ATAT ATTACCAAACAATAGACGAGTAGATTCTAATAGATGA TTTAATTTTGTTTTTTTTT |
| 141 | 187 | 810 | ATAT AAACTACCAAATAATGAACAGATAAATTTCAGTGAGTGA TTTAATTTTTTTTTTTTT* |
| 143 | 185 | 685 | ATAT ATTACCAAACAATAGACGAGTAGATTCTAATAGAT TTTTTTTGTTTTT |
| 141 | 185 | 497 | AT ACTACCAAACGATAAGCAGATAAGTCTCAGTGAATGA TGTAACTTTTTTTTTTTTTTT |
| 134 | 188 | 332 | ATAT AAAACTACCAAACAGTAAATAGATAAGTTCTAATAAGTGAGATAATT TTTTTTTTTT |
| 141 | 188 | 308 | ATAT AAAACTACCAAACGATAGACGAATAAGTTCTGATAAGTGA TATATTTTTTTTTTTT |
| 142 | 185 | 140 | ATAT ATTACCAAACAATAGACGAGTAGATTCTAATAGATG TTTTTTTCTTTTTTT |
|  |  |  |  |
| 163 | 203 | 2,487 | AAA ATAATCAACAAATAGAGAACTGCTAGATGATAGGTGA TATAGATTTTTTTTTTGTT |
| 163 | 211 | 861 | ATATT AACCACAATCAATAAGTAAGAGACTACTAGATGATAGATAA TTTTTTTTTTTTTT |
| 168 | 195 | 232 | ATATATAAAACCACAATCAT CAGATAAGAGACTATTAAGTGATA TTTTTTTTTTTT*† |
| 185 | 216 | 138 | ATATAT AATAAAACCACAATTAGCAAGTAAGAAG GTATCAGATGATAATTATTTTTT† |
|  |  |  |  |
| 204 | 247 | 5,008 | ATATAT ACAAACAAATACAGAGATCGACGAGAAAGAAAGTGAGATT TATTTTTTTTTTTT |
| 195 | 247 | 688 | ATAAATAAATACAAAAATCAGCAAGAAAGAGAGTAAGATTGTGATTAAT TTTTTTTT |
| 199 | 243 | 462 | ATAT ACAAATACAAAAATCGATAGAAAAGAAAGTGAGATCATGATT TATTTTTTTTTTTT |
| 195 | 247 | 457 | ATAAATAAATACAAAAATCGACAGAGAGAAAAGTAGGATTGTGATTAAT TTTTTTTATTTT |
| 195 | 244 | 83,016 | ATAT AACAAATACAAGAGCCGATGAAGAAAAAGGTAGAACTGTGATTAAT TTTTTTTTTTTT |
| 199 | 243 | 9,810 | ATATAT ACAAATACAGAAACTGACGAAAGAGAGAATGAAGTTATGAT CTTTTTTTTTTTTTTTGG |
| 202 | 247 | 2,126 | ATATAT ACAAACAAATATGAGAACTAACAAGAGAGAAAGTGAGATTAT TTTTTTTTTTTT* |
| 201 | 247 | 1,676 | ATATAT ACAAACAAATATGAGAACTAACAAGAGAGAAAGTGAGATTATA TTTTTTTTTTTTTCTCT |
| 204 | 247 | 1,379 | ATATAT ACAAACAAATATGAGAACTAACAAGAGAGAAAGTGAGATT TTTTTTCTTTTTT |
| 199 | 244 | 363 | ATATAT AACAAATACAGAAGCCAACGAGAGAAGGAATAAGATTGTAAT TTTTTTTTTT |
| 202 | 243 | 269 | ATATAT ACAAATACAGAAACTGACGAAAGAGAGAATGAAGTTAT TTCTTTTTTTTTTT |
| 204 | 244 | 149 | ATAT AACAAATACAAGAGCCGATGAAGAAAAAGGTAGAACT TTGATTAATTTTTTTTTTTT |
| 195 | 243 | 130 | ATAT ATAAATACAAAAACTAACGAAAGAAAAGATGGAACTGTGGTTAAT TTTTTTTCTTTT |
| 204 | 243 | 126 | ATAT ACAAATACAGAAACTGACGAAAGAGAGAATGAAGTT TTTTTTTTTTTTTTTTTTT |
| 202 | 244 | 102 | ATAT AACAAATACAAGAGCCGATGAAGAAAAAGGTAGAACTGT TATTATTTTTTTTTTTTTT |
|  |  |  |  |
|  |  |  |  |
|  |  |  |  |
|  |  |  |  |
| 5' | 3' | Reads | COIII gRNA Sequences cont. |
| 229 | 274 | 289 | ATA TACAAAACAAATCTAACAGTGATAGTAACAGATAGATATAGAGATT TTTTTTTTTT |
| 236 | 279 | 575 | ATAT AAAATCACAGAACAGATCTGATAGTAACAGTAATAAGTAAATAT TTTTTTTTTTT |
| 229 | 270 | 559 | ATATAT AAACAAATCTAATGATAACGATGACGGATAGATATAGAGATT TATTTTTTTTTTTTTTTT |
| 238 | 268 | 120 | ATATAAACCACAAT ACAGATCTGACAGTAATGATGATAGGTAAAT TTTTT |
| 238 | 279 | 37 | ATAT AAAATCACAGAACAGATCTGATAGTAACAGTAATAAGTAAAT TTCTTTTTTTTTTTTTT |
| 234 | 264 | 33 | ACAAAACAT ATCTAGCAGTAACAGTGACGAATAGATACAA TTTTTTT |
| 234 | 279 | 29 | ATAT AAAATCACAGAACAGATCTGATAGTAACAGTAATAAGTAAATATAA TTTT |
|  |  |  |  |
| 258 | 299 | 18,740 | ATATAT AAATCAAATAAACTATGTAGAAAGTTACGAGATAGATTTAATA TTTTTTTTTT |
| 265 | 308 | 4,452 | AAA AAAACACAAAAATCAAGTGAACTATGTAGAGGATTGTAAGATAA TTTTTTTTTTT |
| 265 | 310 | 4,418 | ATAAAACACAAAAATCAAGTGAACTATGTAGAGGATTGTAAGATAA TTTTTTTTTTTTT |
| 258 | 300 | 814 | ATATAT AAAATCAAATAAATTACGTAGAGAGTTACAGAATAAGTTTAAT TTTTTTTTTT |
| 267 | 306 | 435 | ATATAT AACACAAAAATCAGATAGACTATGTAGAAGATTGTGAAAT TTTTTTTTTTT |
| 261 | 299 | 181 | ATATAT AAATCAAATAAACTATGTAGAAAGTTACGAGATAGATTT TTTTTTTT |
| 258 | 300 | 347 | ATATAT AAAATCAAATAGATCACGTGAAGAGTTATAGAATAGATTTAAT TTTTTTCTTTTTTT |
| 261 | 307 | 328 | ATAC AAACACAGAAATCAGATAGATCACGTAGAGAGTTATAAGATAAATTT TTTTTTTT |
| 262 | 300 | 244 | ATATATATT AAAATCAGATAAGCCACGTAGAAGATTGTAAAGTGAATT ATTTTTTTTTTTT |
| 261 | 300 | 195 | ATATATATT AAAATCAGATAAGCCACGTAGAAGATTGTAAAGTGAATTT TTTTTTTTT |
| 258 | 289 | 182 | AATCACGTGAAAGATCGTAGAATGAGTTTAAT TTTTTTTTTTTTT |
| 263 | 307 | 71 | ATAC AAACACAGAAATCAGATAGATCACGTAGAGAGTTATAAGATAAAT ATTTTTTTT |
| 257 | 300 | 39 | ATATAT AAAATCAAATAGATCACGTGAAGAGTTATAGAATAGATTTAATA TTTTTTTTTTTT |
|  |  |  |  |
| 293 | 320 | 1,024 | AATACTGT ATATGATGTAGTAAGATATAGAGATTAA TTTTTTTTTT |
| 284 | 321 | 21 | ATATAAA GATACAACGTAATAAGGCATAGAAGTTAAGTGAATTAT TGTTTTTTTTTTTT |
| 293 | 335 | 1 | AAAAAACAATACTGGATATGATGTAGTAAGATATAGAGATTAA TAACTTTTTT |
| 291 | 332 | 1,923 | ATATAT AAACAATACTGGGTACGATGTAATAGAATGTGAAAGTTAAAT TTTTTTTTTTTAAT |
| 293 | 321 | 151 | AATACTGA GATACGACGTGATAAGATATAGAAGTTAA TTTTTTTTTTT |
|  |  |  |  |
| 323 | 365 | 33,179 | ATAT ATAAAACAAACTCGCTATGTAAGAACTGTAAAAAGTGATATT ATTTTTTTTTTTG |
| 330 | 365 | 856 | ATAT ATAAAACAAACTCGCTATGTAAGAACTGTAAAAAA GTGATATT TTTTTTTTT |
| 323 | 365 | 301 | ATAT ATAAAACAAACTCGCTATGTAAGAACTGTAAAAAGCGATATT ATTTTTTTTCTTTTT |
| 323 | 365 | 4,275 | ATATAT ATAAAACAAACTCACTGTGTAAAGATTGTAGAAAGTGATATT ATTTTTTTTTATTTTTTTTTTTTTT |
| 323 | 360 | 139 | ATACAT ATAAACTCACTGCATAAGAATCATAGAGAGTGATATT ATTTTTTTTTTT* |
|  |  |  |  |
| 345 | 391 | 522 | ATATAT ATAATACAACAAGGAGCGTCATAAGTAAAGTGAATTCGTTATAT TTTTTTTTTTTT |
| 345 | 391 | 203 | ATATT ATAATACAACAGAAAATGTCATAAGTGAGATGAATTCGTTATAT TTTTTTTT |
| 345 | 389 | 490 | ATATAA AATACAACAAGAGACGTCGTAAATAGAGTAAATTCGTTATAT TTT |
| 349 | 389 | 461 | ATATATAA AATACAACAAGAGACGTCGTAAATAGAGTAAATTCGTT TTTTTT |
| 347 | 389 | 365 | ATATATAA AATACAACAAGAGACGTCGTAAATAGAGTAAATTCGTTAT TTTTTTTTTTT |
| 343 | 389 | 262 | ATATATAA AATACAACAAGAGACGTCGTAAATAGAGTAAATTCGTTATATAA TTTTTTTTTTTTTTT |
| 357 | 391 | 106 | ATATT ATAATACAACAGAAAATGTCATAAGTGAGATGA TTCGTTATTTTTT |
| 353 | 389 | 79 | ATATAA AATACAACAAGAGACGTCGTAAATAGAGTAAATTT TTT |
| 352 | 388 | 48 | ACAAAT ATACAACAAAAGATGCCGTAGATAAGATAGATTTG GTATATTTT |
| 354 | 390 | 41 | ATATAT TAATACAACAGAAGACGCTATAAGTGAGATAGATT GATTATAT_27_ |
|  |  |  |  |
| 362 | 406 | 101 | ATATAT ATAAACATAAATCAGATAGTACAATGAAGAGTGTTATAGATAA TTTTTTTTT |
| 362 | 406 | 139 | ATAT ATAAACATAAATCAGATAATACAGTGAAGAGTGTCATAGATAA TTTTTT |
|  |  |  |  |
| 376 | 418 | 9,942 | ACA TATAACACAAAAATAGACATAGACTGAATGATGCAGTGAAA TTTTTTTTTTTTG |
| 378 | 422 | 1,634 | ATAT AACTTACAACACAGAGATAGACATAGATCAGATAATGTGATAA TTTTTTTTTTTTT |
| 384 | 418 | 185 | ACA TATAACACAAAAATAGACATAGACTGAATGATGCA TTGAAATTTTTTTTTTT |
| 376 | 418 | 169 | ACA TATAACACAAAAATAGACATAGACTGAATGATGCAGTAAAA TTTTAACTTTTTT* |
| 378 | 422 | 375 | ATAT AACTTACAACACAGAAATAAGCATAGATCAGATAGTGTGATAA TTAATTTTTTTTTTTTTTTTT |
|  |  |  |  |
| 397 | 426 | 15 | AAAACGAT AGCAAATTCATGACGTGAAAATAGATGTAA TTTTTTTTTTT |
| 397 | 436 | 10 | AAAA AAAAAACGAAAGCAGATTCACGGTACAGAGATAGATATAG TTTTTTTTTG |
|  |  |  |  |
| 411 | 449 | 20,772 | ATATATA ATATAAGGTAAATGAGAGACGAGGGTAGACTTGTGATAC TATTTTTTTTTTTT |
| 409 | 438 | 1,420 | ATAATAAGGTAT ACAGAGAACGGAAGCAGACTTATGATATAA TTTTTTTTTTTT |
| 413 | 449 | 224 | ATATATA ATATAAGGTAAATGAGAGACGAGGGTAGACTTGTGAT TTTTTTTTTT |
| 410 | 449 | 125 | ATATA ATATAAGGTAAATGAGAGACGAGGGTAGACTTGTGATATA TTTTTTTTT |
| 413 | 452 | 545 | ATATAT AACATATAAGGTAAATAGAAGATGGAAGCGAATTTGTGAC TTTTTTTTTTTTTTTT |
| 418 | 453 | 60 | ATATATATAAACAAC AGACATATAAGGTAAGTAAGAGATGAAGGTAAATTT TTTTTTTTT |
| 413 | 452 | 20 | ATATAT AACATATAAGGTAAATAGAAGATGGAAGCGAATTTGTGAT TCTTTTT |
|  |  |  |  |
| 418 | 467 | 451 | ATAC TATAATAAACAACAAAATGTGTAAGGTAGATAAGAAGTGAAGGTAAATT ATATTTT |
| 437 | 469 | 238 | A TACATAATAAACAATGAGATATATAAGGTGAAT CGAAAGTGAAATATTTTTTTTTTTT |
| 427 | 460 | 218 | ATATTAT AACAACAAAACGTATAAGGTAAGTGAAAAATGGA TGTAAATTTTTTTTTTTT |
|  |  |  |  |
| 443 | 474 | 37 | A ATAATCACATAATAAATGATAGAACGTATAAG ATAGATGAAAATTTTTTTTTT |
|  |  |  |  |
|  |  |  |  |
| 5' | 3' | Reads | COIII gRNA Sequences cont. |
| 461 | 497 | 66,677 | ATACAT AATACCAATAGAAGACAGAATTGTAGTCATGTGATA TTCATTTTTTTTTTTTTT |
| 461 | 497 | 5,941 | ATACAT AATACCAATAGAAGACAGAATCGTAGTCATGTGATA TTCATTTTTTTTTTTTT |
| 456 | 499 | 936 | ATAT AAAATACCAATAAAGAACAGAATTATAGTTGTATGATAGATAA ATTTTTTTTTTTT |
| 462 | 497 | 371 | ATACAT AATACCAATAGAAGACAGAATTGTAGTCATGTGAT TTTTTTTTTTT |
| 461 | 497 | 139 | ATACAT AATACCAATAGAAGACAAAATTGTAGTCATGTGATA TTCATATTTTTTTTTTT |
| 457 | 499 | 6,621 | ATATT AAAATACCAATAGAAAATGAGACTGTGATTATATGATGAATA TTTTTTTTTTTTTT* |
| 458 | 499 | 3,602 | ATATT AAAATACCAATAGAAAATGAGACTGTGATTATATGATGAAT TTTTTGTTTTTTTT |
| 454 | 499 | 295 | ATAT AAAATACCAATAAAGAACAGAATTATAGTTACATGATAGATAATA TTTTTTTTTT |
| 462 | 499 | 185 | ATATT AAAATACCAATAGAAAATGAGACTGTGATTATATGAT TTTTTTCTTTTTTTT |
| 460 | 501 | 185 | AAA AAAAAATACCAGTAGAAGATAAGACCATAATCATGTGATAA TTTT |
| 461 | 499 | 107 | ATATT AAAATACCAATAGAAAATGAGACTGTGATTATATGATG TTTTTTGTTTTTTT |
|  |  |  |  |
| 483 | 522 | 288 | AAATA ATCAACAAATTAAATGAATCTAAAAGGTATCAGTGAAAA TTTTTTTTTTTTTT |
|  |  |  |  |
| 491 | 539 | 145,031 | ATA TAAATAAAATGTATTTGTCAATGGATTAGATGAATTTAGAGAATATT TTTTTTTTTT |
| 488 | 539 | 4,441 | ATA TAAATAAAATGTATTTGTCAATGGATTAGATGAATTTAGAGAATATTAAT TTCTTTTTTTT |
| 490 | 539 | 1,630 | ATA TAAATAAAATGTATTTGTCAATGGATTAGATGAATTTAGAGAATATTA TTTTTTTTCTTTTTT |
| 498 | 539 | 1,118 | ATA TAAATAAAATGTATTTGTCAATGGATTAGATGAATTTAGAA TATTAATAG TTTT |
| 495 | 539 | 1,052 | ATA TAAATAAAATGTATTTGTCAATGGATTAGATGAATTTAGAGAAT TTTTTTTTTTTGTT* |
| 491 | 539 | 849 | ATA TAAATAAAATGTATTTGTCAATGGATTAGATGAGTTTAGAGAATATT TTTTTTTTTT |
| 487 | 539 | 847 | ATA TAAATAAAATGTATTTGTCAATGGATTAGATGAATTTAGAGAATATTAATA TTATTTTTTTTTTT |
| 486 | 539 | 653 | ATA TAAATAAAATGTATTTGTCAATGGATTAGATGAATTTAGAGAATATTAATAG TTTT |
| 504 | 535 | 599 | ATAT ATAAAATGTATTTGTCGACGAGTTAAATGGAT GTAGAAGATTTTTTTTTTTT |
| 491 | 539 | 482 | ATA TAAATAAAATGTATTTGTCAATGGATTAGATAAATTTAGAGAATATT TTTTTTTTGTT |
| 502 | 539 | 448 | ATA TAAATAAAATGTATTTGTCAATGGATTAGATGAATTT TAGAATATTTTTTCTTTTT |
| 491 | 539 | 353 | ATA TAAATAAAATGTATTTGTCAATGGATTAGATGAATTTAGAAAATATT TTTTTTTTT |
| 504 | 535 | 1,604 | ATAT ATAAAATGTATTTGTCGACGAGTTAAATGGAT GTAGAAGATTTTTTTTTTTTTTT |
|  |  |  |  |
| 528 | 565 | 188 | ATATAT AAAATTAACAAGTGAATCACTAACAGATAGATAGAATG ATATTTTTTTTTTTT* |
| 528 | 564 | 181 | ATATT AAATTAACAGATAAGCCACTGACAAATAGATAGAGTG ATATTTTTTTTTTTT |
| 524 | 564 | 77 | ATATAT AAATTAACAAATAGACTACTAATAAGTGAGTAAGATGTATT AATTTATATATTTT* |
| 525 | 563 | 3,592 | ATATAT AATTAACAAGTAGATCACTGACAAATAGATGAGATGTAT AATTTTTTTTTTTTT* |
| 528 | 565 | 640 | ATATAT AAAATTAACAGATAGATCATTAACGAGTAGATAAAGTG ATATTTTTTTTTTT |
| 523 | 563 | 336 | ATATAT AATTAACAAGTAGATCACTGACAAATAGATGAGATGTATTT TTTTTTTT |
| 526 | 564 | 420 | ATATT AAATTAACAAATAAACTATTAATGGATGAGTGAGATGTA ATTATTTTTTTTTTTTTTT |
| 527 | 563 | 144 | ATATAT AATTAACAAGTAGATCACTGACAAATAGATGAGATGT TTTTTTTTTTTTATTT* |
|  |  |  |  |
| 548 | 592 | 99,540 | ATATAT AAACCTAAATCAAGAACATAGAACAGAGAGATTAGTGAGTAAATT TTTTTTTTTTTG |
| 555 | 594 | 27,720 | ACAT AAAAACCTAAACTGAGAATACGAGACAAAGAAATTAGTGA TTAAATTTTTTTTTTTT |
| 547 | 592 | 1,442 | ATATAT AAACCTAAATCAAGAACATAGAACAGAGAGATTAGTGAGTAAATTA ATTTTTTTTTTT |
| 551 | 592 | 599 | ATATAT AAACCTAAATCAAGAACATAGAACAGAGAGATTAGTGAGTAA TTTTTTTTTTTTT |
| 554 | 592 | 504 | ATATAT AAACCTAAATCAAGAACATAGAACAGAGAGATTAGTGAG AAAATTTTTTTTTTTTT |
| 558 | 592 | 309 | ATATAT AAACCTAAATCAAGAACATAGAACAGAGAGATTAG GGAGTAAATTTTTTTTTTTTTT |
| 548 | 592 | 238 | ATATAT AAACCTAAATCAAGAACATAGAACAGAGAGATTAGCGAGTAAATT TTTTTTTTT |
| 550 | 592 | 176 | ATATAT AAACCTAAATCAAGAACATAGAACAGAGAGATTAGTGAGTAAA ATTTTTTTTTTT |
| 545 | 592 | 150 | ATATAT AAACCTAAATCAAGAACATAGAACAGAGAGATTAGTGAGTAAATTATT TTTTTATTTT |
| 558 | 594 | 147 | ACAT AAAAACCTAAACTGAGAATACGAGACAAAGAAATTAG GGATTAAATTTTTTTTTTT |
| 558 | 593 | 128 | ACATT AAAACCTAAACTGAGAATACAAGACAGAGAGATTAA GGATTAAATTTTTTTTTTTTT |
| 548 | 592 | 114 | ATATAT AAACCTAAATCAAGAACATAAAACAGAGAGATTAGTGAGTAAATT TTTTTTTTTT |
| 551 | 593 | 84,835 | ACAAT AAAACCTAAACCGAGAACATAGAGCAGAGAAGTTAGTAGATAA TTTTTTTTTTTTT |
| 555 | 593 | 6,897 | ACATT AAAACCTAAACTGAGAATACAAGACAGAGAGATTAATGA TTAAATTTTTTTTTTTTTT |
| 552 | 593 | 4,207 | ACAAT AAAACCTAAACCGAGAACATAGAGCAGAGAAGTTAGTAGATA TTTTTTTTTTTTT |
| 556 | 593 | 1,803 | ATATAT AAAACCTAAATCAGAGACGCAGAATAGAGAGATTGATA TATTTTTTTTTT* |
| 553 | 593 | 1,137 | ACAAT AAAACCTAAACCGAGAACATAGAGCAGAGAAGTTAGTAGAT TTTTTTTTTTTT |
| 551 | 596 | 722 | A AAAAAAACCTAAACCGAGAACATAGAGCAGAGAAGTTAGTAGATAA TTTTTTCTTTTTTTT |
| 557 | 593 | 486 | ACATT AAAACCTAAACTGAGAATACAAGACAGAGAGATTAAT TTTTTTTTTTTT |
| 551 | 603 | 456 | ACAAAAAAACCTAAACCGAGAACATAGAGCAGAGAAGTTAGTAGATAA TTTTT |
| 555 | 593 | 206 | ATAT AAAACCTAAACCAAAGATATGAGACAGAGAGATTAGTGA TATGTTTTTTTTTTTTT |
|  |  |  |  |
|  |  |  |  |
|  |  |  |  |
|  |  |  |  |
|  |  |  |  |
|  |  |  |  |
|  |  |  |  |
|  |  |  |  |
|  |  |  |  |
|  |  |  |  |
| 5' | 3' | Reads | COIII gRNA Sequences cont. |
| 585 | 629 | 85,916 | ATATA GAACTCAATCATAATATGAAGCAATAACAATGAAGAGATTTAA TTTTTTTTTTTT |
| 587 | 629 | 9,106 | ATATA GAACTCAATCATAATATGAAGCAATAACAATGAAGAGATTT TTTTTTTTTTT |
| 586 | 629 | 1,991 | ATATA GAACTCAATCATAATATGAAGCAATAACAATGAAGAGATTTA TTTTTTTTTTTC |
| 585 | 631 | 1,447 | ATAT ATAAACTCAATCATAGTATAAGATGACGACAATGAGAAGATTTAA TTTTTTTTTTTTC |
| 585 | 629 | 276 | ATATA GAACTCAATCATAATATGAAGCAATAACAATGAAAAGATTTAA TTTTTTTTTTTT |
| 592 | 629 | 269 | ATATA GAACTCAATCATAATATGAAGCAATAACAATGAAGA TTTAATTTTTATTTTTTTT |
| 591 | 629 | 263 | ATATA GAACTCAATCATAATATGAAGCAATAACAATAAGAGA TTTAATTTTTTT |
| 588 | 629 | 221 | ATATA GAACTCAATCATAATATGAAGCAATAACAATGAAGAGATT AATTTTTTTTATTTT |
| 594 | 629 | 150 | ATATA GAACTCAATCATAATATGAAGCAATAACAATGAA TAGATTTAA TTTTTT |
| 580 | 622 | 145 | ATATAT ATCATAATACAAGGCAATGACGACGAGAAGATTTAGATTAA TTTTTTTTTTT |
| 590 | 629 | 131 | ATATA GAACTCAATCATAATATGAAGCAATAACAATGAAGAGA ATTAATTATTTTTT |
| 603 | 634 | 17,313 | AT ATAACAAACTTAATCGTAATATGAAACAACGA GAATGAGAAAATTTTTTTTTTTTC |
| 585 | 628 | 8,519 | ATATAT AACTCAATCATAATACGAGATGATAATGACGAAGAGATTTAA TTTTTTTTTTTTT |
| 580 | 624 | 3,564 | ATATA TAATCATAATACAGAACGATGGCAGTGAAGAGATTTAGATTAA TTTTTTTTTTTT |
| 587 | 630 | 2,850 | ATATATAT CAAACTCAATTGTAGTACGAGACAATAATGATGAGAAGATTT TTTTTTTTTT |
| 587 | 628 | 628 | ATATAT AACTCAATCATAATACGAGATGATAATGACGAAGAGATTT TTTTTTTT |
| 585 | 630 | 582 | ATATATAT CAAACTCAATTGTAGTACGAGACAATAATGATGAGAAGATTTAA TTTTTTTTTTT |
| 586 | 628 | 231 | ATATAT AACTCAATCATAATACGAGATGATAATGACGAAGAGATTTA TTTTTTTTTTT |
| 587 | 631 | 135 | ATATAT ACAAACTCAGTCATAGTATAAGACAGTGATAATGAGAGAATTT TTTTTTTTTTTT |
| 591 | 630 | 112 | ATATATAT CAAACTCAATTGTAGTACGAGACAATAATGATGAGAAG TTTTTTTTTTTTT |
|  |  |  |  |
| 604 | 647 | 10,315 | ATACAAAAAC AAAAACCAAACGATGAACTTGATTGTAGTATAAGATAATA TTTTTTTTTTTTT |
| 605 | 647 | 7,606 | ATACAAAAAC AAAAACCAAACGATGAACTTGATTGTAGTATAAGATAAT TTTTTTTTTTTAT |
| 604 | 643 | 250 | ATACAAAAACAAAAT ACCGAGCGACAGATTTGATTGTAGTATAAGATAATA TTTTTTTTTTT |
| 607 | 647 | 637 | ATACAAAAAC AAAAACCAAACGATGAACTTGATTGTAGTATAAGATA TTTTTTTTTGTTTTTTTTTTT |
| 604 | 643 | 173 | ATACAAAAACAAAAT ACCGAGCGACAAGTTTGATTATAGTATAAGATAATA TTTTTTTTTTTTT* |
| 605 | 643 | 150 | ATACAAAAACAAAAT ACCGAGCGACAAGTTTGATTATAGTATAAGATAAT TTTTTTTTTTTTTTT* |
|  |  |  |  |
| 635 | 669 | 482 | ATATACAATGCAAACTTA CATGACTGGTTTTATAGAGATGAGAGATTAA TTTTTTTTTTTCTT |
| 635 | 669 | 268 | ATATACAATGCAAACTTA CATGACTGGTTTTATAGAGATGAGAGATTAA TTTTTTTTTTTCTCT |
| 635 | 676 | 60 | ATATACAATGT ACTCTCATAATTGGTTTCATAGAGATAGAAGATTAA TTTTTTTTTTTTTTT |
| 637 | 669 | 42 | ATATACAATGCAAACTTA CATGACTGGTTTTATAGAGATGAGAGATT TTTTTTTT* |
| 633 | 679 | 25 | ATATA CAAACTCTTATAACTGGTTTTACGAGAATGAGAAATTAAAT TTTATTTTTTTTTTT |
|  |  |  |  |
| 659 | 691 | 649 | ATAT TAAATAACAATGCGAATTTTCATAGTTGGTT CATAGATACAATTTTTTTTTTTT |
| 653 | 682 | 39 | AT ATGTGAACTCTTATAACTGGTTTTGTAG TGATGATAGATTTTTTTTTTTTTTT |
|  |  |  |  |
| 669 | 717 | 758 | ATAAT AGAACACCACAGCTTAATGTAGTAGATGGCAGTGTAAATTTTT TTTTTTTATT |
| 669 | 722 | 374 | ATAT AATCAAAAACACCGTAACTTGATGTAGTAGATAGTAGTGTAAATTTTT TTTTTTT |
| 669 | 706 | 354 | ATATAGAACCAAAAACAG TGCAATTTAGTGTGATAGATGATAGTGTAAATTTTT TTTTTTT* |
| 669 | 715 | 160 | ATATAGAACCAAT AACATCGCGACTTAGTGTGATAAGTAATAGTGTAAATTTTT TTTTTTT |
| 684 | 722 | 1,329 | ATATAT AACCAAAAACACTGCGATTTGATGTAATAAGTGAC TGTGTAAATTTTTTTTTTT |
| 689 | 726 | 1,181 | ATAT ATAGAACCAAGAACACCATAGTTTGATGTGATAG TGATAGTGTAAATTTTTTTTTTTTTT* |
| 669 | 715 | 911 | ATATAGAACCAT AACACCATGATTTGATGTAGTAAATGATGATGTAAATTTTT TTTTTTTTT |
| 669 | 722 | 738 | ATAT AACCAAAAACACTGTAACTTGATGTAGTAGATAGTAGTGTAAATTTTT TTTTTTTT* |
| 695 | 730 | 129 | ATAT TAAAATAGAACTAAAGACACTGTAACTTAGTG AGTAAATGATATTAATTTTTTTTTT* |
| 675 | 715 | 107 | ATATAGAACCAT AACACCATGATTTGATGTAGTAAATGATGATGTAAAT ATTTTTTTT |
| 677 | 715 | 102 | ATATAGAACCAT AACACCATGATTTGATGTAGTAAATGATGATGTAA TTTTTTTTTTT |
| 675 | 717 | 90 | ATAAATT AAAACACCATAATTTGATGTGATAAGTAATGATGTAAAT ATTTTTTTTGTTTTTTTT |
|  |  |  |  |
| 706 | 753 | 31,331 | ATATAT AAATGTAATAGATCTGATGAAAGTGAGGTAGAATTGAGAATATT TTTTTTTTTT |
| 707 | 753 | 8,037 | ATATAT AAATGTAATAGATCTGATGAAAGTGAGGTAGAATTGAGAATAT ATTTTTTTTTTTTTT* |
| 706 | 753 | 6,744 | ATATAT AAATGTAATAGATCCAATGAAGGTAAGATAGAACTGAGAATATT TTTGTTTTT |
| 707 | 753 | 3,791 | ATATAT AAATGTAATAGATCCAATGAAGGTAAGATAGAACTGAGAATAT AATTATTTTTTT |
| 701 | 748 | 2,726 | ATA TAATAAATCCAATGAAGATAAAGTAGAGTCAGAGATATTATGAT ATTTTTTTTTTTTT |
| 706 | 753 | 1,214 | ATATAT AAATGTAATAGATTCAATGAAGGTAAGATAGAACTGAGAATATT TTTTCTTTT |
| 707 | 753 | 1,124 | ATATAT AAATGTAATAGATTCAATGAAGGTAAGATAGAACTGAGAATAT AATTTTTTTTTTTTTT |
| 699 | 748 | 977 | ATATA TAATAAATCCAATGAAGATAAAGTAGAGTCAGAGATATTATGATTT TTTTTTTTTT |
| 707 | 752 | 848 | ATATAT AATGTAATAAATCTAATAGAGATAAGATAGAACTGAGGATAT ATTTTTTTTTTTT |
| 713 | 753 | 595 | ATATAT AAATGTAATAGATCTGATGAAAGTGAGGTAGAATTGAGAAT TTTTTTTTTAACCC |
| 707 | 753 | 206 | ATATAT AAATGTAATAGATCTGATAAAAGTGAGGTAGAATTGAGAATAT ATTTTTTATTTTTT |
| 715 | 753 | 182 | ATATAT AAATGTAATAGATCTGATGAAAGTGAGGTAGAATTGAGA TTTATTTTTTTT |
| 713 | 753 | 154 | ATATAT AAATGTAATAGATCCAATGAAGGTAAGATAGAACTGAGAAT TTTTTTTGTTTTTT |
| 719 | 753 | 131 | ATATAT AAATGTAATAGATCTGATGAAAGTGAGGTAGAATT TAGAATATATTTTTTTT |
| 715 | 750 | 1,457 | ATATAT TGTAATAAATCCGATAGAAGTAAGATAGAACTGAGA TATTTTTTATTTTTTTT |
| 714 | 748 | 688 | ATATA TAATAAATCCAATAAGAATAAGATGGAACTGAAGA GATTATGATTTTTTTTTTT |
| 719 | 746 | 304 | AT ATAAATCCAATAAAAGTGAAATAGAATC TGAGTTTT |
| 707 | 753 | 416 | ATAT AAATGTAATAAATTCAGTAGAAGTAAGATAGAATTGAAGATAT ATATAGT_26_ |
|  |  |  |  |

| 5' | 3' | Reads | COIII gRNA Sequences cont. |
| --- | --- | --- | --- |
| 723 | 765 | 2,739 | ATATAT AACATGCATAAGATGTAGTGAATCTAGTAAGAGTAAGATAG TTTTTTTTTTTTCT |
| 728 | 767 | 1,193 | ATATAT AAAACATGCATAGAGTGTAGTAAGTTCAGTGAAAGTGA TATAGTTTTCTTTT |
| 724 | 765 | 2,604 | ATATAT AACATGCATAAGATGTAGTAGATTCAGTGAAGATAAGATA TTTTTTTTTTGTTT |
| 725 | 765 | 456 | ATATAT AACATGCATAAGATGTAGTAGATTCAGTGAAGATAAGAT TTTTTTTTTT |
| 728 | 767 | 284 | ATATAT AAAACATGCATAGAGTGTAGTAAGTTCAGTGAAAGTGA TATAGTTTT |
|  |  |  |  |
| 736 | 781 | 495 | ATAT ACAAAACACCTAAGAAGATGTGCGTAGAATGTGATAGATTTAAT TTTTCTTTTTTTTTTT |
| 737 | 781 | 159 | ATAT ACAAAACACCTAAGAAGATGTGCGTAGAATGTGATAGATTTAA ATTTTTTTTTTTTTTT |
| 739 | 781 | 40 | ATAT ACAAAACACCTAAGAAGATGTGCGTAGAATGTGATAGATTT TTTTTCTTTTTTTTTTTTTTTTT |
| 735 | 779 | 19 | AAAACACCTAAGAAGATGTGCGTAGAATGTGATAGATTTAATA TTTTTT |
| 750 | 778 | 19 | AAACACCTAGAGAAACGTGCATGAGAT TGTGATAGATTAATTTTTTT |
|  |  |  |  |
| 754 | 790 | 153 | ATATAT TAAACAACAACAGAACGTCTAAGAGAGTATGTATA TGATGTAATTTTTTTTTTTTTTT |
| 754 | 790 | 89 | ATATAT TAAACAACAGCAGAACATCTAAGAGAGTATGTATA TGATGTAATTTTTTTTTTTT |
|  |  |  |  |
| 772 | 815 | 111,116 | ATAT AAATTAAACAGACGTATGAAGCAAGTAGATAGTGATAAGATACT ATTTTTTTTTTTTTT |
| 771 | 815 | 25,628 | ATAT AAATTAAACAGACGTATGAAGCAAGTAGATAGTGATAAGATACTT TTTTTTTTTT |
| 775 | 815 | 323 | ATAT AAATTAAACAGACGTATGAAGCAAGTAGATAGTGATAAGAT TTTTTTTTTTTTTG |
| 778 | 815 | 315 | ATAT AAATTAAACAGACGTATGAAGCAAGTAGATAGTGATAA TTTTTTTATTTT |
| 772 | 815 | 281 | ATAT AAATTAAACAAACGTATGAAGCAAGTAGATAGTGATAAGATACT ATTTTTTTTTTT |
| 772 | 815 | 230 | ATAT AAATTAAACAGACGTATGAAGCAAGTAGATAGCGATAAGATACT ATTTTTTTTATTTTTT |
| 777 | 815 | 188 | ATAT AAATTAAACAGACGTATGAAGCAAGTAGATAGTGATAAG TTACTATTTTT |
| 771 | 814 | 138 | ACATAT AATTAAACAAACGTATAGAGCAAGTAAGTAGCAGTGAAATATTT TTTTTTTGTTT |
| 773 | 815 | 136 | ATAT AAATTAAACAGACGTATGAAGCAAGTAGATAGTGATAAGATAC AATTTTTTTTTTTA |
| 773 | 814 | 1,459 | ATAT AATTAAACAAACGTATAAGACAAGTAGATGGCAGTGAAATAT ATTTTTTTTTTT |
| 771 | 814 | 527 | ATAT AATTAAACAAACGTATAAGACAAGTAGATGGCAGTGAAATATTT TTTTTTTTTTTGT |
|  |  |  |  |
| 796 | 829 | 24 | ATAACAAAACGTGA TATCCATATACAGAGAATTAGATAGATGTATAAA TTTTTT |
| 788 | 829 | 5 | ATATA TATCCATACACAGAAAATTAGATGAACGTGTAAAATGAGTAA TTATTTTTTATTTTTT |
| 788 | 829 | 92 | ATATA TATCCATACACAGAAGATTAAATAGACGTGTAAAGTGAATAA TTTTTTTTTTTTTTT |
|  |  |  |  |
| 802 | 842 | 116 | ATAT AAAACAAAACGTGTATTCATATATGAGAGATTAAGTAAATG ATTTTATTTTTTTTTT |
| 798 | 842 | 386 | AT AAAACAAAACGTGTATCTGTGTATAGGGAGTTAAATGGATGTATA TATTTTTTTTTGTTTT |
| 799 | 842 | 156 | ATAT AAAACAAAACGTGTATCTGTGTATAGGGAGTTAAATGGATGTAT TTTTTTGTTTTTTTTTTTTT |
|  |  |  |  |
| 814 | 854 | 66 | ATAT ATACAATACAAAGAAGCGAAACGTGTATCTATATATGGAAA TTTTTT |
| 828 | 855 | 5 | ATATAT AACACAATACAGAGAGATGAAACGTGTA GATGTATTTTTTTTTTTTTTTTTTTTTTT |
| 829 | 855 | 1 | ATATAT AACACAATACAGAGAGATGAAACGTGT TTTTTTTTTTTTTTTTTTTTTT |
| 830 | 854 | 1 | AT ACATAATACAAAGAGACAGAACGTG ATATCTTTTTTTTTTTTTTTT |
|  |  |  |  |
| 848 | 890 | 9,675 | ATATAT AATCAAACTAAATTGACGAGATGTTGATGTAGATAGATATAAT ATTTTTTTT |
| 846 | 891 | 138 | ATAT AAATCAAACTAAATTAGCAAGGTGTCAGTGTAAATGAGCATGATAT TTTTTTGTTTTTT |
| 838 | 882 | 109 | ATACAA TAAATCAACAAGATGTCGATATAGATGGATATGATATGAGAGAAT TTTTTTCTTTTTTTT |
| 845 | 889 | 2,508 | AT ATTAAACTAAATCAATAAAGTGTCGATGTAGATGAATGTGATATA TATTTTTTTTTTT |
| 846 | 889 | 202 | AT ATTAAACTAAATCAATAAAGTGTCGATGTAGATGAATGTGATAT TTTTTTTTTTT |
| 846 | 891 | 127 | ATAT AAATCAAACTAAATTAGCAAGGTGTCAGTGTAAATGAGCATGATAT TATTTTTTTTTTTTT |
|  |  |  |  |
| 880 | 918 | 1,822 | ATATAA ATCAGAATAAACAGATCGCAATAGAGAGAATTAAGTTAA TATTTTTTTTTTTTTC |
| 882 | 927 | 600 | AA ATATAAAACATCAAGATAAATGGATTGTGATAGAGAAAGTTAAATT TTTTTTTTTTT |
| 880 | 929 | 222 | ATATATAAAACATCAGAATAGACAAATCGTAATAGAGAAAGTTAAGTTAA TTTTTTTTTTTTTT |
| 880 | 921 | 209 | ATAT AATATCAAAATAAACAGATCGTAGTAAAAGAAGTTAGATTAA TTTTTTTTTTTT |
| 882 | 929 | 159 | ATATATAAAACATCAGAATAGACAAATCGTAATAGAGAAAGTTAAGTT TTTTTTTTTTTTT* |
| 881 | 929 | 28 | ATATATAAAACATCAGAATAGACAAATCGTAATAGAGAAAGTTAAGTTA TTTT |
|  |  |  |  |
| 907 | 947 | 787 | ATATAA TATACACACAGATACATAATACGTAGAATGTTAAGATAAGT TTTTTTTTTTTTTGTT |
| 909 | 950 | 126 | ATATA ATTACACACACAGATACGTGATATATAGAATGTTAAGGTAA TATAATTTTTTTTTT |
| 913 | 946 | 558 | ATAC ATACACACAAATATATAACATATAGAGCATTGAG TTAGATAATTTTTTTTTTTTTTT |
| 905 | 944 | 3,472 | ATATAA ACACACAAATATATGGCATATAGAGCATTGAAGTAGATAA TTTTTTTTTTTTT* |
| 905 | 944 | 1,321 | ATATAA ACACACAAATATATGACATATAGAGCATTGAAGTAGATAA TTTTTTTTTATTTTT |
| 920 | 952 | 162 | ATAG AAAATTACACACATGAATACATAGTACATAGAA GATTGATATATTTTTTTTTTTTT |
|  |  |  |  |
| 935 | 977 | 2,920 | ATATA AATCAACAACTGAAAAGATATCAATGAGATTGTACATGTAAAT TTTTTTTTTTTTGTT |
| 940 | 977 | 522 | ATATA AATCAACAACTAAGAAGACACTGATAGAGTTATATGTG ATTAATTTTTTTTTTTTTT* |
| 935 | 977 | 354 | ATATA AATCAACAACTGAAAAGATATCAATGAGATTGTACATGTAAAT TTTTTTTT |
| 939 | 977 | 28 | ATATA AATCAACAACTGAAAAGATATCAATGAGATTGTACATGT TTTTTTTTT |
|  |  |  |  |
| 942 | 983 | 1,160 | ATAT AACTAATCAACAGCTAAGAGAACGTCAATGAGATTATGTG ATTAATTTTTTTTTTTTTT |
| 951 | 981 | 127 | AT ACTAATCAACAACTAGAAGAATATCAGTGA TATACATGTAATTTTTTTTTT |
| 951 | 981 | 25 | AT ACTAATCGACAACTAGAGGGACATCAGTGA TTTATACGTATTTTTTTTTTTTTTT |
|  |  |  |  |
| 963 | 1003 | 111 | ATA TACAAACTACCAATATAAGTTAACTGATCGGTAATTAAGG TTATATTTTTTTTATTTTTT |
| 965 | 1003 | 68 | ATATA TACAAACTACCGATATAAGTTAACTGATTGATAATTAA TGTTCTTTTTTTTTTTTTT |

C) C-Rich Region 3

| 5' | 3' | Reads | CR3 gRNA Sequences |
| --- | --- | --- | --- |
| 34 | 62 | 18 | ATATGT ACAACAAAACCGAGCAATCAGATAT AGAGTGAAATTTTTTTTT |
| 34 | 62 | 3 | ATATAT ACAACAAAACTGAACAATCAAATGT AGTGTGATTTTTTTTT |
| 36 | 64 | 1 | ATCGCAAGGTCGT GGACAACAAAACTGAACAATCAAAT TTTTTTTTTTT |
| 34 | 62 | 1 | ATATAT ACGACAAAACTGAACAATCAAATGT AGTGTGTTTTTTTT* |
|  |  |  |  |
| 41 | 88 | 140,541 | ATATAT AAAATGTACAAACGGACAATGAGAGAACAGTGAAATTAGATGAT ATTTTTTTTTTTTTC |
| 40 | 88 | 34,162 | ATATAT AAAATGTACAAACGGACAATGAGAGAACAGTGAAATTAGATGATT TTTTTTTTTTTT |
| 48 | 87 | 3,434 | ATATAATT AAATGTACAGACAAATGATAGAGAGACGATGAGATTAAGT TATATTTTTTTTTTTT |
| 51 | 86 | 3,016 | AAAAATT AATGTACAAATAAACGATAGAGAGACAGTGAGATTA TGATTTTTTTTTTTTT |
| 51 | 88 | 2,553 | ATAT AAAATGTACAGACGAGCAGTGAAGAGACAGTGAGATTA TACATTTTTTTTTTT |
| 47 | 88 | 1,270 | ATATAT AAAATGTACAAACGGACAATGAGAGAACAGTGAAATTAGATG TTTTTTTTTTT |
| 48 | 88 | 902 | ATATAT AAAATGTACAAACGGACAATGAGAGAACAGTGAAATTAGAT TTTTTTTTGTTTTT |
| 41 | 88 | 693 | ATATAT AAAATGTACAAACGGACAATGAGAGAACAGTAAAATTAGATGAT ATTTTTTTTTTTT |
| 52 | 88 | 648 | ATATAT AAAATGTACAAACGGACAATGAGAGAACAGTGAAATT TTTTTTTTT |
| 51 | 88 | 632 | ATATAT AAAATGTACAAACGGACAATGAGAGAACAGTGAAATTA TATGATAATATTTT |
| 46 | 88 | 468 | ATATAT AAAATGTACAAACGGACAATGAGAGAACAGTGAAATTAGATGA AATATTTTTTTTTTTTTT |
| 40 | 77 | 213 | ATATAT AAAATGTACATACGAACGATAAAAGGGCAGTGAAATTAGATAATT TT |
| 41 | 88 | 212 | ATATAT AAAATGTACAAACGGACAATGAGAGAACAGCGAAATTAGATGAT ATCTTTTTTT |
| 50 | 88 | 182 | ATATAT AAAATGTACAAACGGACAATGAGAGAACAGTGAAATTAG TTGTTAATATTTTTTTTTTT |
| 41 | 88 | 181 | ATATAT AAAATGTACAAACGGACAATGAGAAAACAGTGAAATTAGATGAT ATCTTTTTTTTTTTTTTTT |
| 49 | 88 | 177 | ATATAT AAAATGTACAAACGGACAATGAGAGAACAGTGAAATTAGA AGATATATTTTTTTTTTT |
| 55 | 88 | 170 | ATATAT AAAATGTACAAACGGACAATGAGAGAACAGTGAA TTTTTTGTTTTTTTTTTTT |
| 41 | 88 | 155 | ATATAT AAAATGTACAAACGAACAATGAGAGAACAGTGAAATTAGATGAT ATTTTTTTTTTTTT |
| 40 | 88 | 151 | ATATAT AAAATGTACAAACGGACAATGAGAGAACAGTAAAATTAGATGATT TTTTTGTTTTTT |
| 41 | 88 | 135 | ATATAT AAAATGTACAAATGGACAATGAGAGAACAGTGAAATTAGATGAT AATATTTTTTT |
| 56 | 88 | 133 | ATATAT AAAATGTACAAACGGACAATGAGAGAACAGTGA TTTTTTTTTTTT |
| 41 | 88 | 106 | ATATAT AAAATGTACAAACAGACAATGAGAGAACAGTGAAATTAGATGAT AATATTTTTTTT |
| 40 | 89 | 598 | ATAT AGAAATGTACAAACGAGCAATAAGGGAACAGTGAAATTAGATGATT TCTTTTTTT |
| 41 | 89 | 526 | ATAT AGAAATGTACAAACGAGCAATAAGGGAACAGTGAAATTAGATGAT AATTTTTTTTTT* |
| 40 | 77 | 437 | ATATATAAAATGTACAT ACGAACGATAAAAGGGCAGTGAAATTAGATAATT TTTTTTTTT* |
| 47 | 83 | 285 | ATATAA GTACAAACAAACAGTGAGAAGATAACGAGACTGAGTA TATATTTT* |
| 50 | 88 | 255 | ATAT AAAATGTACAAATAAGCAGTAGAAGAGCAGTGAAATTGA TGATATTTTTTTTT |
| 47 | 89 | 237 | ATAT AGAAATGTACAAACGAGCAATAAGGGAACAGTGAAATTAGATG TTTAATTTTTTTTT |
| 51 | 88 | 152 | ATAT AAAATGTACAGACAAGCAGTGAAGAGACAGTGAGATTA TACAGTTTT |
| 39 | 77 | 112 | ATATATAAAATGTACAT ACGAACGATAAAAGGGCAGTGAAATTAGATAATTA TTTTTTTTTTTT |
| 51 | 86 | 70 | AAAATC AATGTACAAATAAGCGATAAAGAGACAGTGAGATCA TGATTTTTTTTTTTTTTT |
| 48 | 89 | 53 | ATAT AGAAATGTACAAACGAGCAATAAGGGAACAGTGAAATTAGAT TTTTTATTTTTT |
| 47 | 83 | 31 | ATATAA GTACAAACAGACAGTGAGAAGATAACGAGACTGAGTA TATTTATTTTTTTTTTTTTTTTT |
|  |  |  |  |
| 78 | 118 | 573 | ATATAT AATCACAAACAAATAGAAAATGAGAGAGGTGTATGA TACTATTTTTTTTTCTTTTT |
| 93 | 123 | 1,321 | ATATAT AAACAAATCACGAACGAGTAGAAAAT TGGAAGAATGTATTTTTTTTTTTT |
| 78 | 118 | 685 | ATATAT AATCACAAACAAATAGAAAATGAAAGAGGTGTATGA TACTATTTT |
| 89 | 123 | 251 | ATATT AAACAAATCACAAATGAGTAGAGAACGAGA TTGATGTATATTTTTTTTTTTTTTTT |
| 86 | 121 | 243 | ATAC ATAAATCACAAACGAATAAGGGGCAGAAGAG TTGTATGATATTTTT |
| 76 | 124 | 120 | ATATAT AAAACAAATCATAAACGAATAAAGAGTGAGGAAGGTGTATAAAT TTT |
|  |  |  |  |
| 105 | 140 | 15,770 | A TAGATAACAAACATAAGAGCAAGTCACGAG GTAGATATTGATATTTTTTTTTTTTTC |
| 105 | 140 | 692 | A TAGATAACAAACATAAGAGCAAGTCACGAG ATAGATATTGATTTTTATTTTTT |
| 98 | 142 | 442 | AT ATTAAATAACAAACATAGAAATAGATCACAGATAGATGA TTGATGAATTTTTT |
|  |  |  |  |
| 122 | 166 | 27,885 | ATAGAAATCCAATAAGAGACAGAAGCTAGATAATGAGTATAAGA TATTTTTTTTTTTTT |
| 124 | 166 | 212 | ATAGAAATCCAATAAGAGACAGAAGCTAGATAATGAGTATAA TTTTTTTTTTTT |
| 125 | 166 | 139 | ATAGAAATCCAATAAGAGACAGAAGCTAGATAATGAGTATA TTTTTTCTTTTTTTTTT |
| 127 | 166 | 114 | ATAT ACAAAAATCCAATGAAAAATAAAGACTGAGTGATGGATG CAATTTTCTTTTTTTTTT |
| 123 | 162 | 309 | ATATAT AAATCCAATAAGAAATGAAAGCTAGATAGTGAGTATAAG TTTTTTTTTTTTGTTT |
| 124 | 166 | 421 | ATATAT ACAAAAATCCAATGAAAAATAAAGACTGAGTGATGGATGTAA TTATTTATTTTTTTTTTTTTT |
| 129 | 167 | 370 | ATAT AACAAAAATCTAATAGAGAACAGAAACTAAGTAATGAG ATATTTTTTTTT |
| 123 | 161 | 348 | ATATTAT AATCCAATAGAAAGCAGAGACTAAATGATAGATGTAAA TTTTTTATT* |
| 132 | 168 | 110 | ATATAT AAACAAAAATTCAATAGAAAACGAGAACTGAGTAAT TGATATGTTTTT |
| 125 | 167 | 53 | ATATAT AACAAAAATCCGATAAAAGATGGAAACTAAGTGATAGATATA TTTTTTTTTTTTT |
|  |  |  |  |
| 154 | 196 | 1,492 | ATAT ATACAACAATAAACTCGTATTAAGTGAGAGATGAAGATTTAAT TTTTTTTTATTTT |
| 156 | 199 | 136 | ATAT TAAACACAACGATAGATCTATATTAAGTAGAAGATAGAAATTTA TTTATTTTTTTTTTTT |
| 162 | 200 | 2,176 | ATAT ATAAACACAACAGTAGACTCGTATTAGATAGAAGATAGA TATCATTTTTTTTTTTTTTT |
| 156 | 200 | 114 | ATAT ATAAACACGACAATAGATTCATATTAAATAGAAGATAGAGATTTA TTTTTTTTTTTT |
| 157 | 200 | 98 | ATAT ATAAACACGACAATAGATTCATATTAAATAGAAGATAGAGATTT TTTTTTT* |
|  |  |  |  |
|  |  |  |  |
| 5' | 3' | Reads | CR3 gRNA Sequences cont. |
| 190 | 230 | 2 | ATATACA ACATATCAAGTGGTAAGATAAGAGAAGAAAGTAGATGTAAT TTTTTT |
| 190 | 230 | 1,243 | ATATACA ACATATCAAGTGATAAGATAAGAGAAGAAAGTAGATGTAAT TTTTTATTTTTT |
| 192 | 232 | 279 | ATAGATA ACACATATCAGATGATAAGGTAAAGAGAGAGAATAGATATA TTTTTTCTCTTTT* |
|  |  |  |  |
| 226 | 277 | 19 | ATAT AATAACAATATAAACGAACTGGATGATGTATAGTACTTTGATATATA ATTTTTTTTTTTT |
| 231 | 265 | 2 | ATATAACAATAC AAATGAGCTAGATAATGGATGATAGTTTGATAT TTTTTTTTTTTT |
| 237 | 265 | 1 | TATAAAATAACAATAC AAGTGAATTAGATAATGGATGATG TTTCAATTTTTTTTTTTTTTT |
| 241 | 279 | 936 | AT AGAATAACAATATAAACGAACTAGATGATGGATAGTA TCTTTTTTTTTTTTTTTTT |
| 234 | 265 | 528 | ATATAACAATAC AAATGAACTGAGTAATGGATAGTAGTTTGA CAATTTTTTTTT |
|  |  |  |  |
| 293 | 308 | 2 | ATATAT AAATTATTTGCATACT GAGATTAGTGATAGTAAAGTGATTAATTTTTTTTTTTTT |
| 268 | 312 | 125 | AT ATAAAAATTATTTGCATGCTTAAGTGAGTTATAGAGGTAATGATA AATTTTTAT |

D) C-Rich Region 4

| 5' | 3' | Reads | CR4 gRNA Sequences |
| --- | --- | --- | --- |
| 25 | 64 | 121 | ATAAT AAAAATGCACAACTAGAATTGAAGTAAAGTGATGATA TATATTTTTTTTGTTTTT |
| 25 | 64 | 596 | ATAAT AAAAATGCACAACTAGAATTGAAGTAAAATGATGATA TATATTTTTTTTTTTTTT* |
| 25 | 64 | 308 | ATATT AAAAATGCACAACTAGAATTGAAATAAAGTGATGGTA TATATTTTTTTTTTTTT |
| 25 | 62 | 83 | ATATAATT AAATGCACAGCCAAAGTTAAGGTAGAATAGTGATA TATTTTTTTTTTTTTT |
|  |  |  |  |
| 48 | 103 | 296 | ATA TATATAAAACACAGACATACTAAGTAAGAGAAAGAGAGGTGTATGATT TTTTTCTTTTTT |
| 48 | 103 | 175 | *ATA TACATAAAACACAGACATACTAAGTAAGAAAAAGAGAGATGTATGATT TTTTTTTTTTTT* |
| 65 | 98 | 108 | ATATAT TAAAACACAAATACATCAGATAGAAAGAGA TTGAGTGTATAATTTTTTTTTTTTTTTTT |
| 51 | 103 | 18 | ATA TACATAAAACACAGACATACTAAGTAAGAAAAAGAGAGATGTATG TTTTTTTATTTTTTTTTTTTT |
| 59 | 103 | 11 | ATA TACATAAAACACAGACATACTAAGTAAGAAAAAGAGAGAT TTTTTTTTCTTTTTT |
| 52 | 103 | 10 | ATTATA TACATAAAACACAGACATACTAAGTAAGAAAAAGAGAGATGTAT TTTTTTTTTTTTTTT |
|  |  |  |  |
| 87 | 134 | 7,793 | ATATAT AAACAACAATAGAGTATATCATAGACTGTATATGAAGCATAAAT TTTTTTTTTT |
| 89 | 134 | 426 | ATATATAT AAACAACAATAGAGTATATCATAGACTGTATATGAAGCATAA CTTTTTTTT |
| 88 | 134 | 208 | T ATATAT AAACAACAATAGAGTATATCATAGACTGTATATGAAGCATAAA ATTTTTCTTTTTTT |
| 93 | 139 | 200 | AAACAAAACAACAGTGAAATATACCGTAGATTGTATGTGAAAT TATATATTTTTT |
| 90 | 134 | 143 | ATATATAT AAACAACAATAGAGTATATCATAGACTGTATATGAAGCATA TTTTTTTTATTTT |
| 93 | 142 | 2,052 | A AAAAAACAAAACAACAGTGAAATATATCGTAGATTGTATGTGAAAT TATTTTT |
| 90 | 138 | 584 | ATAT AACAAAACAACGATAAGATGTATCATGAGCTGTATATGAGATATA TTTTTTTTATTTTTTTTTTTTTTTT |
| 91 | 138 | 139 | ATAT AACAAAACAACGATAAGATGTATCATGAGCTGTATATGAGATAT TTTTTTTTTTTTT |
|  |  |  |  |
| 127 | 171 | 6,971 | ATAT ATATACTCACACAAATAGATGACAGAGATAGAAAGTAAGATGATA TATTTTTTTTTTTTTTC |
| 128 | 171 | 204 | ATAT ATATACTCACACAAATAGATGACAGAGATAGAAAGTAAGATGAT TTTTTTTTTTTTT |
|  |  |  |  |
| 154 | 192 | 3,603 | ATATTA AACTATAACAAGGCAGATAGAACGTACCTATATAGATAA TTTTTTTTTTTTTT |
| 174 | 200 | 6 | AT AAATAAACAACTATAATAAGACAAGTG CGATGTACTTTTTTTTTT |
| 166 | 196 | 5 | ATATA AAACAACTATAACAAAATAGATAGAATGTGC GTATTTTTT |
| 171 | 200 | 3 | AAATAAACAACTATAATGAAGTGAGTGAAG GTACGTTTTTTTTTTTT |
| 169 | 196 | 1 | ATATA AAACAACTATAACAAAATAGATAGAATG GGCGTATTTTTT |
| 171 | 196 | 1 | A AAACAACTATAATGAAGCAGATAGAA GGTACGTATATTTTTTTTTTTTTTTTT |
|  |  |  |  |
| 186 | 232 | 31 | ATAAAAAATCACAGCCTAAAATGACGAGAGAAAGTAAATGGTTATA TAGATTTTT |
| 186 | 225 | 22 | ATATAT ATCACAACCTAAGATAACGAAAAAGAGCAGATAATTGTA TATTTT |
| 186 | 228 | 7 | ATATAT AAAATCACAACTTAGAATGACGAAAGAGAATAGATAGTTGTA TATTGTTTTCTTTTTTTTTTTTTTTAT |
| 186 | 228 | 4 | ATATAT AAAATCACAACTTAGAATGACGAAAGAGAATAGATAGTT TTAAAAAATTTTTTTTTTTTTTTT |
| 186 | 232 | 166 | ATAAAAAATCACAGCCTAAAATGACGAGAGAAAGTAAATGGTTATA TTTTTTTCTTTTTTTT |
| 186 | 227 | 152 | ATAT AAATCACAACCTGAAATAGCAGAGAAGAGTAAATGATTATA TTTTTTTTTTTTT |
| 189 | 232 | 25 | ATAAAAAATCACAGCCTAAAATGACGAGAGAAAGTAAATGGTT TTT* |
| 189 | 227 | 12 | ATATAT AAATCACAACCTGAAATAGCAGAGAAGAGTAAATGATT TTTTTTTTT* |
|  |  |  |  |
| 213 | 261 | 14,358 | ATAT ATAAACTATACAATTGAAGCACTGATAGAAGGTTGTGATTTAA TTTTTTTTTTTT |
| 213 | 261 | 264 | ATAT ATAAACTATACAGTCAAGACACTGATGAGAGATCGTGATTTAA TTTTTTTTTTTTT |
| 216 | 261 | 114 | ATAT ATAAACTATACAATTGAAGCACTGATAGAAGGTTGTGATTT TTT |
| 222 | 261 | 112 | ATAT ATAAACTATACAATTGAAGCACTGATAGAAGGTTG ATTTAATATTTTTTTT |
| 213 | 258 | 5,062 | ATATAT AACTATACAGTCGAGACATCAATGAGAGATTATGACTTAA TTTTTTTTTTTTC |
| 213 | 259 | 1,370 | CCCTCGGCCGCAGCGATATA AAACTATACAATTAGAGCATCAGTAGAAGATTGTGACTTAA TTTTTTTTTTTTTT |
| 213 | 259 | 1,074 | ATATA AAACTATACAATTAGAGCGTCAGTAGAAGATTGTGACTTAA TTTTTTTTTTTTTTT* |
| 213 | 258 | 490 | ATATAT AACTATACAATTGAGATATCAGTGAAGAATTGTGATTTAA TTTTTTTTTTT |
| 215 | 258 | 340 | ATATAT AACTATACAGTCGAGACATCAATGAGAGATTATGACTT TTTTCTTTTTTTTT |
| 223 | 258 | 183 | ATATAT AACTATACAGTCGAGACATCAATGAGAGATT TTTTT |
|  |  |  |  |
| 251 | 300 | 23 | AAAAAAAAAAATAAACAGAATTGTGACGTCATAAAGAAGATAGATTATAT TTTTTTTTTTG |
| 242 | 284 | 9 | AAAAAAA AAAATTATAACGTCATAGAAGAGATAGACTATATGATTAA TTTTTTTTT |
| 251 | 295 | 77 | ATAT AGAAAATAAACAGAATTGTGACGTCATAAAGAAGATAGATTATAT TTTT |
| 250 | 295 | 64 | AT AGAAAATAAACAGAATTGTGACGTCATAAAGAAGATAGATTATATA TTTTTT |
| 253 | 295 | 25 | ATAT AGAAAATAAACAGAATTGTGACGTCATAAAGAAGATAGATTAT TTTTTTTCTTTTTTTT |
| 253 | 298 | 22 | AAAAAAAAATAAACAGAATTGTGACGTCATAAAGAAGATAGATTAT TTT |
| 244 | 295 | 20 | AT AGAAAATAAACAGAATTGTGACGTCATAAAGAAGATAGATTATATAGTT TTTTTTTTTTTT |
| 250 | 297 | 17 | AAAAAAAATAAACAGAATTGTGACGTCATAAAGAAGATAGATTATATA TTTTTTTTTTTT |
| 251 | 301 | 16 | AAAAAAAAAAAATAAACAGAATTGTGACGTCATAAAGAAGATAGATTATAT TTTTT |
| 255 | 295 | 11 | ATAT AGAAAATAAACAGAATTGTGACGTCATAAAGAAGATAGATT TTTT |
|  |  |  |  |
| 280 | 320 | 643 | AAAA AAAAAACACAAGGCGAGATAGAGAAAAGAGATAAATAAGAT GTTTTTATTTT |
| 279 | 320 | 93 | AAAAA AAAAAACACAAGGCGAGATAGAGAAAAGAGATAAATAAGATT TTTTT |
|  |  |  |  |
|  |  |  |  |
|  |  |  |  |
|  |  |  |  |
|  |  |  |  |

| 5' | 3' | Reads | CR4 gRNA Sequences cont. |
| --- | --- | --- | --- |
| 307 | 351 | 725 | ATAAAAT ACCAAACAGATCAGATAAAGGCAGTGATATAGAGAATATAAAAT TTTTTTTTTTT |
| 311 | 353 | 675 | ATAT AAACCAAACAAGCTGAATAAGAACAGTGATATAGAAGATATA TATTTTTTTTTTTTTT |
| 306 | 351 | 259 | AAAAT ACCAAACAGATCAGATAAAGGCAGTGATATAGAGAATATAAAATA TTTTTTTTTTTT |
| 309 | 351 | 214 | ATAAAAT ACCAAACAGATCAGATAAAGGCAGTGATATAGAGAATATAAA TTTTTTTTTTTT |
| 324 | 354 | 140 | AT AAAACCAGACAAACTGAATGAAGACAGTAACGTAGAAGATAT TTTTTTTTTT |
| 307 | 352 | 485 | TTGTTTGGTTGATTAAAT AACCAAACAAGTCGAGTAGAGACAGTGATATAAAAGGTATAAAAT TTTTTTTTT* |
| 310 | 353 | 374 | ATAT AAACCAAACAGACCAAGTGAAGATGGCAGTATAAGAGATATGA TATATTTTTTTTTTT |
| 309 | 352 | 134 | AAAT AACCAAACAAGTCGAGTAGAGACAGTGATATAAAAGGTATAAA TTTTTTTTTATTTTTT* |
| 310 | 352 | 42 | AAAT AACCAAACAAGTCGAGTAGAGACAGTGATATAAAAGGTATAA TTTTTTTTTTTT |
| 312 | 352 | 38 | AAAT AACCAAACAAGTCGAGTAGAGACAGTGATATAAAAGGTAT TTTTTTTTTTTTT |
|  |  |  |  |
| 343 | 388 | 863 | ATAT ATAACACAAAACATAACAGAGAGTATAGAGAGAAATTGAATGA TTTTTTTTTATTTTT |
| 340 | 390 | 2,308 | ATAT AAATAACACAAAATACGACGAGAAATATAAGAGAGAATTGAGTAAATT TTTTT* |
| 343 | 388 | 255 | ATAT ATAACACAAAACATAACAGAGAGTATAGAGAGAAATTGAATGA TTTTTTTTTTTTTTT* |
|  |  |  |  |
| 374 | 417 | 411 | AAAA AAAAAACAACATAGAAAGTGAATCAGAGAATGACATAAGATATA TTTTCTTTTTT |
| 377 | 417 | 447 | A AAAAAACAACATAGAAAATAAGTCAGAGAGTAATATGAGAT TGTTATAATTTTT |
| 374 | 415 | 423 | ATATAT AAAACAACATAAGAGATGAATCAAGAGGTAATATAAGATATA TTTTTTTTTTTTTTTTT |
|  |  |  |  |
| 404 | 457 | 4,211 | AAAAAAAAAAACAAAGACAAAGAAATCACTCAGAATAGAAGATGGTATAA TTTTTTTTTCTTTT |
| 405 | 456 | 620 | AAAAAAAAAACAAAGACAAAGAAATCACTCAGAATAGAAGATGGTATA TTTTTCTTTTTTT |
| 406 | 457 | 392 | AAAAAAAAAAACAAAGACAAAGAAATCACTCAGAATAGAAGATGGTAT TTTTTTTATTTT |
| 405 | 457 | 406 | AAAAAAAAAAACAAAAACAGAAAAACTGTCTAAGATAGAGAATGATATA TTTTTTTCTTTTTTT |
| 409 | 457 | 185 | AAAAAAAAAAACAAAGACAAAGAAATCACTCAGAATAGAAGATGG GATAAATTTTTTTT |
| 404 | 458 | 227 | AA AAAAAAAAAAAACAAAGACAAAGAAATCACTCAGAATAGAAGATGATATAA TTTTTTTT* |
| 405 | 456 | 186 | AAAAAAAAAACAAAAACAAGAAGACTATCTGAGGTAGAAAATGATATA TTTTTTTATTTTTT |
| 411 | 443 | 97 | ATATAAACAT AAACAAAGAGACCATCCGAAATAGAGAAT TTTTTTTCTTTTTTT |
| 405 | 457 | 34 | AAAAAAAAAAACAAAGACAAAGAAATCACTCAGAATAGAAGATGATATA TTTTTTTTT |
| 406 | 458 | 25 | A AAAAAAAAAAAACAAAGACAAAGAAATCACTCAGAATAGAAGATGATAT TTTTTTTTT* |
|  |  |  |  |
| 442 | 489 | 3,197 | ATAACAACCACAGATAGAAATGAACGTAAATGAGAGAGAAAGATGAAA TTTTTTTTTTTTG |
| 440 | 489 | 238 | ATAACAACCACAGATAGAAATGAACGTAAATGAGAGAGAAAGATGAAAGT TTTTTTTTTTT |
| 442 | 487 | 730 | ATAA AACAACCACAAGTAGAAATAGACATAAATGAGAGAGAAAGATAAAA TTTTTTTTTTTT |
| 442 | 486 | 246 | ATAT ATAACCACAAATAGAGACAAATGTAAGTAAAGAGAGAAAGTAAAA TTTTTTTGTTTTTTTTTTT |
| 443 | 487 | 208 | ATAA AACAACCACAAGTAGAAATAGACATAAATGAGAGAGAAAGATAAA TTTTTTCTCTCTTTCTCCCTTT |
| 446 | 487 | 149 | ATAA AACAACCACAAGTAGAAATAGACATAAATGAGAGAGAAAGAT TTTTTTTTTTT |
|  |  |  |  |
| 453 | 497 | 1,153 | AT ACAAAATAACAACAATCATAAGTAAGAATAGATGTAGATGAGAAA TTTTTTTTTTT |
| 453 | 494 | 282 | ATATAT AAATAACAACAATCACAGATGAAGGTAGATATAAGTGAGAAA TTTTTTTTTTTTTG |
| 453 | 497 | 2,511 | AT ACAAAATAACAATGATCACAAATAAGAGTGAATGTAGATAGAGAA TTTTTTTTTTTTTTTGGAAT |
| 453 | 494 | 306 | ATATAT AAATAACAACAATCACAGATGAAGGTAGATATAAGTGAGAAA TTTTTTTTTTTTT* |
|  |  |  |  |
| 475 | 519 | 766 | ATATAT AATAACAACAATAATCAGATTAGCAGAGTAATGATAGTTATAAAT TTTTTTTTTTTTG |
| 480 | 524 | 390 | ATACAAATAACAACAATAGCCAAGTTAATAGAGTGATGATGATTA ATTTTTTTTTTTTTT |
| 478 | 524 | 1,913 | ATACAAATAACAACAATGATCAGACTAATAGAGTAATAGTGATTATA TTTTTTTTTTTTCT |
| 481 | 524 | 105 | ATACAAATAACAACAATGATCAGACTAATAGAGTAATAGTGATT TTTTTCTTTTTT |
| 479 | 524 | 101 | ATACAAATAACAACAATGATCAGACTAATAGAGTAATAGTGATTAT TTTTTTTTTTTTTTT |
|  |  |  |  |
| 504 | 554 | 146 | AA AAAAAACGCATATAAGTGAGCCTATACATAGATAATGATGATAATT TTT |
| 503 | 547 | 53 | AAAAAAAAAAA GCATATAAATAGATCTATATATGAGTGATAGTGACAATTA TTTTTTTTTTTTTTT |
| 504 | 547 | 50 | AAAAAAAAAA GCATATAAATAGATCTATATATGAGTGATAGTGACAATT TCTTTTTTTTT |
| 507 | 554 | 98 | AAAAA AAAAAACGCATATGAGTAGATCTGTACATAGATAGTGATGATA TTTTTTTTTTTT |
| 507 | 556 | 84 | ATAGAAAACGCATATGAGTAGATCTGTACATAGATAGTGATGATA CTTTTTT |
| 508 | 554 | 37 | CAACTCGACTGCGTGAA AAAAAACGCATATGAGTAGATCTGTACATAGATAGTGATGAT TTT |
|  |  |  |  |
| 542 | 575 | 511 | AAATTTATAAAACCAAT CCGTAATTATCTGAGATGAGAAGTGTATA ATTTTTTTTTTTT |
| 542 | 584 | 997 | ATATTTATAAAACC ATACTGTGATTATCTAAGGTAGAAAGTGTGTA ATTTTTTTTTTTTTTTTTTTGG |
| 542 | 596 | 352 | ATA TTTATAAAACCAATATCATAGTTATCTGAGGTAGAGAGTGTATA ATTTAATGTATTTTTTTTTTTTTTTTTT |
| 537 | 568 | 62 | CATACC TAATTATCTGAAGTAGAAGATGTATGTAAAT TTTTTTTTTTTTTT |

E) Cytochrome b

| 5' | 3' | Reads | CYb gRNA Sequences |
| --- | --- | --- | --- |
| 32 | 59 | 327 | AAATAATAGGGATTTATGATGAGATATG CTGTGGATATTTTTTTTTTTTTT |
| 32 | 60 | 255 | AAAATAATAGGGATTTATGATGAGATATG CTGTGGATATTTTTTTTTTTT |
| 32 | 61 | 190 | AAAAATAATAGGGATTTATGATGAGATATG CTGTGGATATTTTTTTTTTTTT |
| 32 | 64 | 176 | AA AAAAAAAATAATAGGGATTTATGATGAGATATG CTGTGGATAGTTTTTTTTT |
| 32 | 62 | 65 | GT AGAAAATAATAGGGATTTATGATGAGATATG CTGTGGATAGTTTTTTTTTTTT |
| 32 | 64 | 35 | AA AAAAAAAATAATAGGAGTTTATGATGGAATATG CTGTGGATATTTT* |
| 32 | 64 | 17 | AA AAAAAAAATAATAGGAGTTTATGGTGAGATATG CTGAGAGTATTTTT |
|  |  |  |  |
| 53 | 91 | 18,713 | AAAAAA AAAAGACAGTGTGAATTTCTGAGTAATAAAGGGAATAAT TTTTTTTTTTT |
| 51 | 91 | 10,649 | AAAAAA AAAAGACAATATAGATTTCTGGGTGATAAAAGGGATAATAA CTTTTTGTTTTT |
| 52 | 91 | 908 | ATAA GAAAGACAATATAGGTTTCTGGGTAATGGAGAGAATAATA TTTTTTTTTTTTCTTT |
| 54 | 91 | 339 | AAAAAA AAAAGACAATGTAGATTTCTGAGTAATGGGGAGGATAA CTATTTATTTT* |
| 54 | 91 | 8,406 | AAAA AAAAGACAATGTAGATTTCTGAGTAATGGGGAGGATAA CTATTTTT* |
| 54 | 91 | 2,265 | AAAAAA AAAAGACAATGTAGATTTCTGAGTAATAGGGAGGATAA CTATTTTCTTTTTTTTTTG |
| 53 | 91 | 204 | AAAAAAA AAAAGACAATGTAGATTTCTGAGTAATGGGGAGGATAAT TTTTTTT |
| 56 | 91 | 95 | AAAAAAA AAAAGACAATGTAGATTTCTGAGTAATGGGGAGGAT TTTTT* |

F) Maxicircle Unidentified Reading Frame II

| 5' | 3' | Reads | Murf II gRNA Sequences |
| --- | --- | --- | --- |
| 30 | 79 | 2,605 | ATAG AAAGCACAAAAATAAAATTAAATTAGAGTAATTGGATGTTAAAATT TTTCTTTTTTT |
| 30 | 79 | 125 | ATAG AAAGCACAAAAATAAAATTAAATTAGAGTAATTGAATGTTAAAATT TTTTTTTT |
| 34 | 79 | 17 | ATAG AAAGCACAAAAATAAAATTAAATTAGAGTAATTGAATGTTAA CATTTTTTTTTTTT |
| 33 | 79 | 15 | ATAG AAAGCACAAAAATAAAATTAAATTAGAGTAATTGAATGTTAAA TTTTGTTTT |

G) NADH Dehydrogenase 3

| 5' | 3' | Reads | ND3 gRNA Sequences |
| --- | --- | --- | --- |
| 33 | 76 | 313 | ATATATT ATAAACCATGATATCGAAAATGGGTGTAGAAATGATGATA TTTTTTTTTTTT |
| 31 | 79 | 70 | ATAT ATAATAAACCACAGTATCAGAGACAGATATAGAAGTGATGATAGT TTTTTTCTTTTTT |
| 30 | 73 | 625 | ATAT TAAACCACAATATCAGAAATAAGTGTAGAAATAGTGATAATA TTTTTTTTTTTT |
| 31 | 79 | 511 | ATAT ATAATAAACCACAGTATCAGAGACAGATATAGAAGTGATGATAGT TTTTTTTTT |
| 42 | 79 | 397 | ATAT ATAATAAATCACAGTATCAGAGACAGATATAGAA TGATGATAGTTTTTTTTTTTT |
| 33 | 79 | 26 | ATAT ATAATAAACCACAGTATCAGAGACAGATATAGAAGTGATGATA TTTTTTTTTCTTTTTT |
|  |  |  |  |
| 63 | 113 | 1,006 | ACATAAGAAACATAAAGAAAAATCTGTGAGTAGAGTGATAAGTTATAAT TTTTTTTTTTT |
| 65 | 113 | 411 | ACATAAGAAACATAAAGAAAAATCTGTGAGTAGAGTGATAAGTTATA TTTTTTTTGTTTTTT |
| 64 | 113 | 84 | AT ACATAAGAAACATAAAAAGAAATCTGTAAGTAGAGTAGTAAGTTATAA GTTTTTTTTTTTTTT |
| 63 | 108 | 59 | ATACAT AAAAACATAAAAAGAAACTTATAAGTAGAGTGATAGATTATAAT TTTTTTTTT |
| 65 | 108 | 33 | ATACAT AAAAACATAAAAAGAAACTTATAAGTAGAGTGATAGATTATA TTTT |
| 66 | 108 | 27 | ATACAT AAAAACATAAAAAGAAACTTATAAGTAGAGTGATAGATTAT TTTTTTTTTTTT |
| 68 | 108 | 14 | ATACAT AAAAACATAAAAAGAAACTTATAAGTAGAGTGATAGATT TTTTTTTTTT |
|  |  |  |  |
| 99 | 143 | 826 | AT ATGAAAACAATCAAAGAAGTGTGATAGAAAGTATAAAAGGTATAA TTTTTTTTTTT |
| 98 | 141 | 542 | ATAA GAAAACAATCAGAGAAATGCGGTAAAAGATATAAGAGATATAAA TTTTTTTTTTTT |
| 101 | 141 | 501 | ATATAA GAAAACAATCAGAGAAATGCGGTAAAAGATATAAGAGATAT TTTTTTTTT |
| 98 | 141 | 240 | ATATAA GAAAACAATCAGAGAAATGCAGTAAAAGATATAAGAGATATAAA TTTTTTTTTTTTT |
| 100 | 141 | 199 | ATATAA GAAAACAATCAGAGAAATGCGGTAAAAGATATAAGAGATATA TTTTTTTTTTT |
| 99 | 141 | 188 | ATATAA GAAAACAATCAGAGAAATGCAGTAAAAGATATAAGAGATATAA TCTTTTTTTTTTTTT |
| 100 | 141 | 168 | ATATAA GAAAACAATCAGAGAAATGCAGTAAAAGATATAAGAGATATA TTTTTTTTTATTTTTTTT |
| 99 | 141 | 52 | ATATAA GAAAACAATCAGAGAAATGCGGTAAAAGATATAAGAGATATAA TTTTTTTTTT |
| 98 | 141 | 24 | ATATAA GAAAACAATCAGAGAAATGCGGTAAAAGATATAAGAGATATAAG TTTTTTTTT |
| 100 | 141 | 21 | ATATAA GAAAACAATCAGAGAAATGCGGTAAAAGATATAAGAGATATG TTTTTTTTTTTTTTTTTTTT |
|  |  |  |  |
| 130 | 170 | 507 | ATATAT CAAATCACACGAAGATCATAGATGACGATGAAGGTAGTTAA TATTTTTTTGTTTTTTTTTT |
| 130 | 170 | 413 | ATACAT CAAATCACACGGAAATCATAGATGGCAATGAAGATAGTTAA TTTTTTTTTTT |
| 130 | 174 | 2,397 | ATA TATACAAACTACACGGAGATCACAAATAACAGTGAGAATGATTAA TTTTTTTTTTTT |
| 132 | 174 | 133 | ATA TATACAAACTACACGGAGATCACAAATAACAGTGAGAATGATT TTTTTTTTTTTTTTT |
|  |  |  |  |
| 158 | 205 | 412 | T ATGTATAAAACATTAAATGTGAGTTTGTGTCGTGTAGATTATGTGAA TTTTTTTTTTTTTTT |
| 155 | 189 | 236 | AAATAAAACACC AACGTGAATTTATATTGTATAGATCGTATGAGAAT ATTTTTTTTTTGTTGT |
| 158 | 205 | 1,756 | ATAT ATGTATAAAACATTAAATGTGAGTTTGTGTCGTATAGATTATGTGAA TTTTTTTTTTTTCT |
|  |  |  |  |
| 190 | 229 | 4,864 | ATATAT AACAACTAACAGAATATAGATCTGATGTATAAGATATCA TTTTTTTTTTTTTG |
| 190 | 229 | 123 | ATATAT AACAACTAACAGAATATAGATCTGATGTATAAGATATCG TTTTTTTTTTTTT |
| 190 | 233 | 227 | ATAT AACAAACAACTGATGAGACATAGATTCGATGTATAAGATATCA TTTTTTTTTTTTT |
| 198 | 234 | 139 | ATAT AAACAAACAACTAATAAAATGTAAATCTGATGTGTGA TATACTTTTTTTT |
| 191 | 233 | 40 | ATAT AACAAACAACTGATGAGACATAGATTCGATGTATAAGATATC TTTTTTTTTTT |
|  |  |  |  |
| 223 | 263 | 3,225 | TTAT ATACAAATAATGGGATTTAACGATATAGAGGATGAATGATT TTTTTTTTTTT |
| 222 | 263 | 3,165 | ATAT ATACAAATAATGGGATTTAACGATATAGAGGATGAATGATTA TTTTTTTTTTTTTAT |
| 222 | 264 | 384 | AAAAAT AACACAGATAATGGAATTTAATGATATGAGAAATGGATGATTA TTTTT |
| 223 | 264 | 164 | AAAAT AACACAGATAATGGAATTTAATGATATGAGAAATGGATGATT TCTTTTTTTTTTTTTTTTT |
| 223 | 263 | 106 | ATAT ATACAAATAATGGGATTTAACGATATAGAGAGTGAATAATT TTTT |
| 222 | 263 | 232 | AAAT ATACAGATAATGGGATTTAACGATGTGAGAGATAGATAATTA TTTTTTTTTTTTTTTT |
| 223 | 263 | 177 | AAAAAT ATACAGATAATGGGATTTAACGATGTGAGAGATAGATAATT TTTTT |
| 220 | 263 | 32 | AAAT ATACAGATAATGGGATTTAACGATGTGAGAGATAGATAATTAAT TTTTTTTTTTTTT |
|  |  |  |  |
| 253 | 299 | 260 | ATAT GATAAAACAACACTATTATGAGAACGAGTGATAGAATATAGATAAT TTCTTTTTTTTTTTTTT* |
| 253 | 299 | 106 | ATAT AATAAAACAACACTATTACAAAGATAGACAGTGAGATATAGATAAT TTTTTTTCTTTTT |
| 253 | 298 | 83 | ATACAT ATAAAACAACACTATCATAGAAGCAGACAGTGAGATATGAGTAAT TTTTTTTTCTTTTTT |
| 253 | 299 | 133 | ATAT AATAAAACAACACTATTACAAAGATAGACAGTGAGATATAGATAAT TTTTTTT |
| 252 | 299 | 89 | AT AATAAAACAACACTATTACAAAGATAGACAGTGAGATATAGATAATG ATTTTTT |
| 253 | 299 | 57 | ATAT AATAAAACAACACTATTACGAAGATAGACAGTGAGATATAGATAAT TTTTTTTTTTTT |
| 252 | 299 | 22 | AT AATAAAACAACACTATTACGAAGATAGACAGTGAGATATAGATAATG ATTTTTTTTTTTTTTTT |
|  |  |  |  |
| 284 | 329 | 786 | ATAT AAAACCACAAAAATAGAAAGCTATAATAGAGATAGAATAATGTTA ATTTTTT |
| 288 | 320 | 435 | ATTTTAAGTT AGAGTGAGAAATTGTAGTGGAAATAAGATGATA AAATTTTTTTTTTT |
| 285 | 329 | 282 | ATAT AAAACCACAAAGGTAGAAGATCGTAATAGAGATAGAATAATATT TTTTTTTTT |
| 300 | 333 | 268 | AT AACAAAAACCACAGAGATGAGAGATTGTAATAAG TATAGTGATAATTTTTTTTTTTTT |
| 285 | 329 | 168 | ATAT AAAACCACAAAAATAGAAAGCTATAATAGAGATAGAATAATGTT TTTTTTTCTTTT |
| 282 | 329 | 64 | ATAT AAAACCACAAAAATAGAAAGCTATAATAGAGATAGAATAATGTTATT CTTTTTTTTT |
| 285 | 328 | 3 | AT AAACCACAAAGATAGAAGGCCATAATAGAGATAAAATAATATT TTTTTTTTTT |
|  |  |  |  |
|  |  |  |  |
|  |  |  |  |
|  |  |  |  |
| 5' | 3' | Reads | ND3 gRNA Sequences cont. |
| 322 | 369 | 48,018 | ATAT AATACCACATGAATCTTATATGTACGATGGAAGATGAGAATTAT TTTTTTTTTTTTT |
| 324 | 369 | 5,034 | ATAT AATACCACATGAATCTTATATGTACGATGGAAGATGAGAATT TTTTTTTTTTG |
| 321 | 369 | 3,062 | ATAT AATACCACATGAATCTTATATGTACGATGGAAGATGAGAATTATG TTTCTTTTTT |
| 322 | 369 | 142 | ATAT AATACCACATGAATCTTATATGTACGATGGAAGATGAAAATTAT TCTTTTTTTTTT |
| 322 | 370 | 721 | AT AAATACCACATGAATCTTATATGTACGATGGAAGATGAGAATTAT TTTTTTTTTTTT |
| 321 | 370 | 291 | AAATACCACATGAATCTTATATGTACGATGGAAGATGAGAATTATG CAGTTTTTTTT |
| 320 | 368 | 149 | ATAT ATATCACACAAATTCTATACATATAATAGAGAATGAGAGTTACAA TTTTTT |
| 324 | 370 | 43 | AT AAATACCACATGAATCTTATATGTACGATGGAAGATGAGAATT TTTTT* |
|  |  |  |  |
| 347 | 388 | 95 | ATATGTAAAC AATATACGTGATTTCAGAGATACTATGTGAATTCTAT TTTATTTTTTTTTTTTTTTTTT |
| 345 | 388 | 82 | ATATGTAAAC AATATACGTGATCTCAGAGATACTATGTGAATTCTATGT TTTTTTT |
| 355 | 388 | 1,307 | ATATAAACAAAC AATATATGTGGTTTCGAAAGTGTCATGTGAATT ATTTTTTTTTTTT |
| 350 | 388 | 633 | ATAGAT AATATACGTGATCTTAGAGGTACCATGTGAGTCT GAGTTATTTTTTTTTTTT |
| 349 | 388 | 376 | AGTATGCGTGATTTTAGAGATATTGTATGAATTTT TTTTTTTT |
| 355 | 388 | 104 | AGTATGCGTGATTTTAGAGATATTGTATGAATT ATATTTTTTTTTTTTTTT* |
|  |  |  |  |
| 402 | 438 | 417 | ATATA TATAATACAACAAGGAGCGTCATAAGTAAAGTGAA TTCGTTATATTTTTTTTTTTTT |
| 402 | 438 | 128 | ATAT TATAATACAACAGAAAATGTCATAAGTGAGATGAA TTCGTTATATTTTTTTTT |
| 402 | 435 | 753 | ATATATAA AATACAACAAGAGACGTCGTAAATAGAGTAAA TTCGTTTTTTTT |
| 403 | 438 | 126 | ATAT TATAATACAACAGAAAATGTCATAAGTGAGATGA TTCGTTATTTTTT |

H) NADH Dehydrogenase Subunit 7

| 5' | 3' | Reads | ND7 gRNA Sequences |
| --- | --- | --- | --- |
| 36 | 69 | 100,761 | ATATA ATAAATGTAAAGAGACTATTGAGAGTGGCATAAG TGATATTATTTTTTTTTTTTTT |
| 35 | 69 | 765 | ATATA ATAAATGTAAAGAGACTATTGAGAGTGGCATAAGG GATATTATTTTTTTTTTT |
| 36 | 71 | 240 | AAAT ATACAAATGTAAAGAAGCTATCAGAGGTAATATAAG TGATATAATTTTTTTTTTTTT |
| 36 | 69 | 121 | ATATA ATAAATGTAAAGAGACTATTGAGAGTGACATAAG TGATATTATTTTTTTTTTTTT |
| 38 | 71 | 113 | ATAT ATACAAATGTAAAGAGACTATCGAGAGTGACATA TGTGATATTATTTTTTTTTTT |
| 28 | 71 | 35,079 | AAAT ATACAAATGTAAGAGAACTGCCGAAAGTAACGTAGAATGATATT ATTTTTTTTTTTT |
| 27 | 71 | 20,487 | AAAT ATACAAATGTAAGAGAACTGCCGAAAGTAACGTAGAATGATATTT TTTTTTTT |
| 31 | 71 | 546 | ATAAAT ATACAAATGTAAGAGAACTGCCGAAAGTAACGTAGAATGAT TTTT |
| 34 | 71 | 402 | AAAT ATACAAATGTAAGAGAACTGCCGAAAGTAACGTAGAAT TTTGTTTTTTTTTTT |
| 36 | 69 | 354 | ATATA ATAAATGTAAAGAGACTATTGAGAGTGGCATAAG TGATATTATTTTTTTTTTTT |
| 24 | 71 | 327 | ATAAAT ATACAAATGTAAGAGAACTGCCGAAAGTAACGTAGAATGATATTTGTT TTTT |
| 24 | 71 | 298 | AAAT ATACAAATGTAAGAGAACTGCCGAAAGTAACGTAGAATGATATTTATT TTTTTTTTTTT |
| 28 | 71 | 194 | AAAT ATACAAATGTAAGAGAACTGCCAAAAGTAACGTAGAATGATATT ATCTTTTTTTTT |
|  |  |  |  |
| 59 | 91 | 4,376 | ATATATAGATGCA GTGGGTCGAATGTAAGATGATATAGATGTGAA TGTTTTTTTTTTTTT |
| 58 | 91 | 1,242 | ATATATAAATACA GTGGACTAAATGTGAAGCGATATAAATGTGAAA TTTTTTTTTTTT |
|  |  |  |  |
| 108 | 137 | 13 | ATATAATAAACAACATAAAGTGCCATGTACT#CGAGAT TTTTAG |
| 95 | 132 | 1 | AT ATAAACAACATAAAATACTATGTGATGTAGGAT CTGTGAATTAATTTTTTTTT |
|  |  |  |  |
| 124 | 170 | 3,944 | ATAC ATCAATATAAACGATAGATTTACCGTAGAAGTATAGTGAATAAT TTTTTTTTTTATTT |
| 124 | 168 | 3,640 | ATATA CAATATAAACAGTAGATTCACTGCAGAAGTATGATAGATAAT TTTTTTTTTTG |
| 139 | 170 | 1,507 | AT ATCAATATAAACAATAAGTTCGTCATAGA TTTACAGTAGATAATTTTTTTTTTTT |
| 121 | 166 | 396 | ATACAT ATATAAACAATGAATTCACTGTGAAGATACGATAGATGATATA TTTTTTTTTGTTTT |
| 139 | 170 | 384 | AT ATCAATATAAACAATGAGTTCGTCATAGA TTTACAGTAGATAATTTTTTTTTTTT |
| 121 | 166 | 178 | ATAT ATATAAACAATAAATTCATCGTGAAGGTACAGTAAATGATATA TTTTTTTTTTTTTTTT |
| 122 | 166 | 26 | ATAT ATATAAACAATAAATTCATCGTGAAGGTACAGTAAATGATAT TTTTTTTTTTTTTTT |
|  |  |  |  |
| 152 | 190 | 988 | AAATTACGATGCAT AATAATCTATGGTACAGTTGATATGAGTGATAA TTTTTTTCTTTT |
| 147 | 199 | 563 | ATATATA CACGATGCAGATAATCTATAGTATGATTGATATAAGTGATAAATTT TTTTTTTTT |
| 150 | 199 | 200 | ATATATA CACGATGCAGATAATCTATAGTATGATTGATATAAGTGATAAAT ATTTTTTTTTTTT |
| 151 | 199 | 2,089 | AAA TACGATGTAAATAACCTGTAGTATAGTTAGTGTAGATAGTGAA ATTTTTTTTTTTT* |
| 147 | 179 | 295 | ATATATAAATGCAAATAACG TGTAATACAGTCAATATAGATGATAAATTT TTTTTTTTT |
| 152 | 190 | 143 | AAATTACGATGCAT AATAATCTATGGTACAGTTGATATGAGTGATAA TTTTTTTTTTTTT |
|  |  |  |  |
| 246 | 269 | 2,470 | ATATCAAC ACATAATCTGACTTGTCGGAGTAT CTAAAGGAATAAATTTTTTTTTTTTTT |
| 246 | 269 | 837 | ATATCAAC ACATAATCTGACTTGTCGGGGTAT CTAAAGGAATAAATTTTTTTTTTTT† |
|  |  |  |  |
| 261 | 311 | 3,259 | ATAT AACATAAAGACAATAAGTGCTTATTACAGTGAACATTGATATAATTT TTTTTTTT |
| 261 | 293 | 2,874 | ATATATAAGACAAC GATGCTCATTATGATAGATACTGATGTAATTT TTTTTTTTTT |
| 261 | 310 | 146 | ATAT ACGTAAAGACAATAGGTGTTCATTGCAGTAGATATTGATGTAATTT TTTTTTTTTT |
| 260 | 293 | 133 | ATATATAAGACAAC GATGCTCATTATGGTAGATACTGATGTAATTTA TTTTTTT |
| 261 | 293 | 1,105 | ATATATATAAGACAAC AGTGCTCGTTACAGTGAATATTGATGTAATTT TTTTTTTTTTT* |
|  |  |  |  |
| 297 | 338 | 888 | AT ATAAATAACATCGCAACGTATATTCGAAATGTAGAGATA CATTTTTTTTTTTGT |
| 295 | 338 | 765 | ATATA ACAAACAACATCGTAATATGTGCTCGGAGTATAGAGATAAT TAAATATTTTTTTTTTTTT |
| 292 | 334 | 91 | ATACT ACAACATCGCGATATATACTTGGAATGTAAAGGTGATAAA GTTTTTTTTTTT |
| 292 | 324 | 394 | ATATATAACAACATCG AGCATATACTCAGAATATAAAGGTGATAAA GTTTTTTTTTTTT |
| 292 | 324 | 178 | ATATATAACAACATCG AGTATATACTCAGAATATAAAGATGATAAA GTTTTTTTTT |
| 293 | 324 | 90 | ATCGG AGCATATACTTGAGATATAAAGATGATAA TTTTTTTTTTT |
|  |  |  |  |
| 327 | 373 | 2,128 | A TATAATTAATAAACGTATAAATGTGCAGTGTGACGATGAATGATATT ATTTTTTTTTTTTT |
| 327 | 365 | 1,225 | ATACAT ATAAACGTATAGGTGCATAATGTAACGATGAATGATGTT AATTTTTTTTTTT |
| 327 | 365 | 20,110 | ATACAT ATAAACGTATAGATGCGCAATGTAACGATGAATGATGTT AATTTTTTTTTTTT |
| 327 | 373 | 997 | A TATAATTAATAAACGTATAAATGTGCAGTGTGACGATGAATGATATT TTTTTTTGTTTTT |
| 330 | 378 | 517 | AAA TTACAATTAATAGACGTATAAGTGCATAGTGTAGTGATGGATAAT TTTTTTTTTTTTTTTAT |
| 329 | 378 | 111 | AAA TTACAATTAATAGACGTATAAGTGCATAGTGTAGTGATGGATAATG ATTTTTTTTTTTTT |
|  |  |  |  |
| 352 | 398 | 283 | ATATA GAAAACTACAGGTAAATTCTGCAATTAGTAGACGTGTAAAT ATTTTTTT |
| 353 | 402 | 3,043 | ATATA TATTAAAACTATGGGTAGATTCTGTAATTAATAGACGTGTAAA ATTTTTTTTTTTTT* |
| 352 | 385 | 602 | ATATATAAAACTACGA GTAGATTCTATGATTGATGAACGTGTAAAT TTTTTTTTTTT* |
| 354 | 402 | 556 | ATATA TATTAAAACTATGGGTAGATTCTGTAATTAATAGACGTGTAA TTTTTCTTTTTT |
| 352 | 402 | 173 | ATATA TATTAAAACTATGGGTAGATTCTGTAATTAATAGACGTGTAAAT TTCTTTT |
|  |  |  |  |
| 390 | 424 | 2,283 | ATATTAA ATACATGATATACGCGATAGATTATTAGAGTTATG AGTTAATTTTTTTTTTTTGTTT |
| 391 | 427 | 200 | AAATT ACCATACATGATATATACAGTGAACTATTAGAATTAT AGGTAATGTTTTTT |
| 390 | 424 | 162 | ATATTAA ATACATGATATGCGCAGTAGACTATTAAAGTTATG AGTTAATTTTTTTTTTTT |
| 391 | 424 | 298 | AAATTACA ATACATGATATATACAGTGAACTATTAGAATTAT AGGTAATTTTTTTTTTTTTT |
|  |  |  |  |
|  |  |  |  |
|  |  |  |  |
| 5' | 3' | Reads | ND7 gRNA Sequences cont. |
| 412 | 452 | 15,183 | ATATATAA GGAGACAAATGATCTAGATTCGAGACTGTATATGATATAT TTTTTTTTTTTTT |
| 414 | 451 | 223 | ATATATAACAAC GAGACAGATAATCTAGATTTGAAGTTATATGTGATAT TTTTTTTTTTTT |
| 416 | 452 | 199 | ATATATAA GGAGACAAATGATCTAGATTCGAGACTGTATATGAT TTTTTTTATTTTT |
| 416 | 464 | 5 | AT ATTATAATAACGGAGATGAGCAATTTAGATTCAGAGTTATATGTGAT TTTTTTTTTTTTT |
| 407 | 450 | 3,126 | ATATA AGACAAACAATCTAAATCTGAGACTGTATATGATATGTATAAT TTTTTTTTTT |
| 414 | 458 | 2,332 | ATAT ATAACGGAGACAGATAGCTTGAATCTAAGGTTGTATGTGATAT TTTTTTTTTTTT |
| 410 | 450 | 132 | ATATA AGACAAACAATCTAAATCTGAGACTGTATATGATATGTAT TTTTTTTTTTTTT |
|  |  |  |  |
| 453 | 485 | 3 | ATAAAATGTCATCAATTA GTTACGTTCTTTGAGTGATTGTGATAAT ATTTTTTTTTTTTT |
| 453 | 485 | 207 | ATAAATAACATCAATTA GTTACGTTCTTTGAGTGATTGTGATAAT ATTTTTTTTTTTTT |
|  |  |  |  |
| 486 | 530 | 2,523 | ATATAA GCATACGACAATTACGATATGAGTCAGAGAATGTTGTTAATTT TTTTTTTTTTT |
| 499 | 526 | 2,002 | ATATATAA ATACGACAATCATAACGTGAATCAGAGA CTGTGATTAATTTTTTTTTTT |
| 486 | 530 | 1,136 | ATATAA GCATACGACAATTACGATATGAGTCAGAGAATGTTGTTAATTT TTTTTTTTTTT |
| 486 | 528 | 344 | ATATA ATACGACAATCACGATATAGATTAAAAGATGTTGTTAATTT TTTTTTTTTT* |
|  |  |  |  |
| 508 | 548 | 1,129 | ATAT AAATCATGAAAGCTGAGTGTGTATGGCAGTTACGATATA TGTTAATTTTTTTTTTTTTT |
| 508 | 544 | 116 | ATATAAAA CATGGAAGCTAAGTGTGTATGATGATTATGATATA TGATTAATTTTTT |
| 508 | 553 | 1 | ATA TAATAAAACCATGAGAGTTGGATGTATATGATGATCGTGATATA TTTTTTTTTTTTATTTT |
|  |  |  |  |
| 526 | 569 | 3,050 | ATATAT ATCATCAAGAATATCTAATGAGACTATGAGAGTTAAATGTATA ATTTTTTTTTTTT |
| 526 | 569 | 4,850 | ATATAT ATCATCAAGAATATCTAATGAGACTATGAGAGTTAAATGTATA ATTTTTTTTTTTTT |
| 527 | 569 | 351 | ATATAT ATCATCAAGAATATCTAATGAGACTATGAGAGTTAAATGTAT TTTTTT |
|  |  |  |  |
| 540 | 576 | 544 | ATAATAAT AAACAAAATCATTGAGAGTATCTGATAGAGTTATGA TAGTCATTTTT |
| 531 | 574 | 291 | AAAT ACAAAATCATCAGGGATACTTGGTAAGATTGTGAAAGTTAAGT TTTTTTTGTTTTATAT |
| 540 | 571 | 8,794 | ATATAAT AAATCATCAAGAATATCTGATAGAACTGTGA TTGCTAATTTTTTTTTTTTTT* |
| 540 | 571 | 1,694 | ATACAAT AAATCATCAAGAGTATCTAATAGAACTGTGA TTGCTAATTTTTTTTTTTTTT |
|  |  |  |  |
| 564 | 615 | 1,896 | AT ATATTATCAACAACAATAGAAGATTGGCGAAATTAGAGATAGAATTATT TTTTTTTTTTTT |
| 567 | 615 | 360 | AT ATATTATCAACAACAATAGAAGATTGGCGAAATTAGAGATAGAATT TTTTTTTTT |
| 562 | 603 | 267 | ATATT ACAACAACAAGAAATCAATGAAGTCAGAGATAAAGTTATTAA TTTTTTTTTGTTTTT |
| 564 | 596 | 173 | TAGATATCAACAACAT CAGAGAATCAATGAAACTAGAGATAGAGTTATT TTTTTTTTTT |
| 567 | 611 | 1,316 | ATACA TATCAACAACGACAAGAGATCAGTGAAATTAGAAGTAAAGTT TTTTTTTTTTTTT* |
| 564 | 615 | 1,043 | AT ATATTATCAACAACAATAGAAGATTGACGAAATTAGAGATAGAATTATT TTTTTTTTTT |
| 564 | 611 | 754 | ATACA TATCAACAACGACAAGAGATCAGTGAAATTAGAAGTAAAGTTATT TTTTTTTTT* |
| 567 | 615 | 703 | AT ATATTATCAACAACAATAGAAGATTGACGAAATTAGAGATAGAATT TTTATTTTTTTTTTT |
| 563 | 611 | 188 | ATACA TATCAACAACGACAAGAGATCAGTGAAATTAGAAGTAAAGTTATCA TTTTTT |
| 564 | 611 | 146 | ATACA TATCAACAACGACAAGAGATCAGTGAAATTAGAAGTAAAGTTATC TTTTTTTTTTTT |
| 568 | 603 | 131 | ATATT ACAACAACAAGAAATCAATGAAGTCAGAGATAAAGT ATTTTTTTT |
|  |  |  |  |
| 584 | 630 | 831 | ATAAT TAACAAACAAATATGATATTATCAGTGACAGTGAGAAATTGATA TTTTTTTTTATTTTT |
|  |  |  |  |
| 596 | 642 | 20 | ATA TATAACAATCCATAGCAGATAGACGTGATATTATTGATGATAGT TTAAATTTTTTTTTTTTTTTTTT |
| 596 | 640 | 512 | AAAT TAACAATCCATAACAAGTGAGCGTGATATTGTCAGTGATAAT TTTTTTTTTTT |
| 596 | 648 | 159 | ATAAATCATAACAATCTATAATAGACGAGCGTGATATTGTCAATGATAAT TTTTTTTTTT |
| 596 | 642 | 32 | ATA TATAACAATCCATAGCAGATAGACGTGATATTATTGATGATAAT TTTTTTTTTTTT |
|  |  |  |  |
| 629 | 670 | 1,258 | ATAT GATAAACGATTACCTACAGATAATGAGTCATAGTGATTTATA TTTTTTTTTTTTT |
| 632 | 674 | 463 | ATAT ATAAAATAAACGATTACCTGTGAATGATAGATTATGATGATTT TTTTTTTTTTT |
| 630 | 674 | 419 | ATAT ATAAAATAAACGATTACCTGTGAATGATAGATTATGATGATTTAT TTTTTTTATTTTTTTTTTTTTTTTTT* |
| 630 | 669 | 299 | ATACAT ATAAACGATTACTCATAGATAGCAAGTCATAGTGATTTAT TTTTTTTTATAGG |
| 630 | 671 | 101 | ATTT AAATAAACGATTACTTACGAGTGACAGATTGTGATGATTTAT TTTTTTTTTTTTTT |
| 629 | 671 | 65 | ATATTT AAATAAACGATTACTTACGAGTGACAGATTGTGATGATTTATA TTTTTTTTTTTTACT |
|  |  |  |  |
| 656 | 699 | 59 | ATATAT ATGACAAACTACGTAAGTGCAGATAAAGTAAGTGATTATTT TTTTTCTTTTTT |
| 666 | 701 | 2,991 | ATAT AGATGACAAACCATGTAGACGTGAATAAGATAG TGATTGATACATTTTTTTTTTTT* |
|  |  |  |  |
| 679 | 727 | 2 | AAAAAA AAAAACTAAATCATATAAATTAAAGGGTGATGAACTGTGTAAAT TTTTTTTTTTTTT |
| 679 | 711 | 1 | ATAAGTCAGAAAGTGATAGATCGTGTAAAT TTTTTTTTTTTTTTT |
| 679 | 725 | 1,728 | AAATTAAATCATATAAGTTGGAAGATGGCAGATTGTGTGAAT TGTAGTTTTTTTTTTTTTTTT |
| 679 | 727 | 373 | AAAAA AAAAACTAAGTCATATAAGTCAGAAAGTGATAGATCGTGTAAAT TTT |
|  |  |  |  |
| 711 | 758 | 3,229 | ATATAT ACGAGACAAAATATCACTTAGATTATTAGAGATTGAGTTATA ATTTTTTTTTTTTT |
| 710 | 741 | 22 | AA TTAGACTATTAGAGATTGAGTTATAT TTT |
| 709 | 741 | 2 | AAAACGAGACAAGATACCAA TTAGACTATTAGAGATTGAGTTATATA TTTTTTTTTTTTTT |
|  |  |  |  |
| 725 | 764 | 55,757 | ATATAA TAACGAACGAGGCAGAGTATCATTTAGACTATTAGA TTCAATTTTTTTTTTTTTT |
| 731 | 759 | 2,867 | ATATATAAT GACGAGATAAGACATCACTTAGACTGT AGAGATTTTTTTTTTTTGG |
| 722 | 765 | 8,175 | ATATA TTAACGAACGAGATAGAACATTGCTTGAGTTATTGAGAAT ATTTTTTTTTTTTTT* |
| 719 | 765 | 552 | ATATA TTAACGAACGAGATAGAACATTGCTTGAGTTATTGAGAATT TTTTTTTTTTTT |
| 735 | 765 | 115 | ATA TTAACGAACGAGATAGAACATTGCTTGAGTT TT |
|  |  |  |  |
|  |  |  |  |
| 5' | 3' | Reads | ND7 gRNA Sequences cont. |
| 756 | 794 | 48,263 | AT ACTAAATAAACGACGATCTTATACTGTATCTGATGAATG TGATATTAATTTTTTTTTTTTTT |
| 757 | 794 | 318 | AT ACTAAATAAACGACGATCTTATACTGTATCTGATGAAT ATGATATTAATTTT |
| 751 | 794 | 174 | AT ACTAAATAAACGACGATCTTATACTGTATCTGATGAATGGGATA TTAATTTTTTTTGTTTTT |
| 755 | 794 | 167 | AT ACTAAATAAACGACGATCTTATACTGTATCTGATGAATGA TATTAATTGTTTTTTT |
| 756 | 794 | 2,829 | AT ACTAAATAAACGACGATCTTATACTGTATCTGATGAATG TGATATTTTTTTTTTTTTT* |
|  |  |  |  |
| 772 | 806 | 367 | ATATA TCATAACAACTAGATAAACGATGATCTCACA GTATAGTTAATTTTTTTTTTTT |
| 782 | 816 | 77 | ATAACTTATAACAACTAGATAGACGG GAATCTTATATTGTAGTTAATTTTTTTTTTTTTTTT |
|  |  |  |  |
| 778 | 830 | 25,793 | ATAT ATAAAACATAAAATATGACTTGTAGCAGTTAAGTGAATGATGAT ATTTTTTTTTTTT |
| 777 | 822 | 5,729 | ATATAT TAAAATACAACTTATGATGACTAAGTGAATGATGATT CAATTTTTTTTTT |
| 775 | 830 | 2,644 | ATAT ATAAAACATAAAATATGACTTGTAGCAGTTAAGTGAATGATGATTTT TTTTTTTT |
| 781 | 833 | 2,373 | AAATA ATAACAAAACATGAGATATAACTTGTAGTGATTAGATGAATGAT TTTTTTTTTTT |
| 780 | 833 | 2,079 | AAATA ATAACAAAACATGAGATATAACTTGTAGTGATTAGATGAATGATA TTTTTTTTTTTTT |
| 778 | 833 | 903 | AAATA ATAACAAAACATGAGATATAACTTGTAGTGATTAGATGAATGATAGT ATTTTTTTTTTTTT |
| 778 | 830 | 450 | ATAT ATAAAACATAAAATATGACTTGTAGCAGTTAGGTGAATGATGAT ATTAAATTTTTTTTTTTTT |
| 778 | 830 | 331 | ATAT ATAAAACATAAAATATGACTTGTAGCAGTTAAGTGAACGATGAT ATTAAATTTTTTTTTTTTTT |
| 778 | 830 | 134 | ATAT ATAAAACATAAAATATGACTTGTAGCAGTTAAGTAAATGATGAT ATTTTTTT |
|  |  |  |  |
| 792 | 845 | 98 | ATAT AAAACAATAATCATGATGAGATATAAAGTGCAGTTTGTGATAATT TTTTTGTTTT |
| 790 | 839 | 52 | ATAT ATAATCATAACAAGATGTAGAGTACGATTTATAGTGATTAA TTTTTTTTTTTT |
| 790 | 839 | 343 | ATAT ATAATCATAACAAGATATAGAGTACGATTTATAGTGATTAA TTTTTTTTTTT |
| 792 | 844 | 190 | ATAT AAACAATAATCATGATGGGATATAAAGTGCAGTTTGTGATAATT TTTTTTTTTT |
| 790 | 839 | 99 | ATAT ATAATCATAACAGAGCATAGAATACAGTTTATAGTGATTAA TTGTTTTT* |
| 790 | 844 | 89 | ATAT AAACAATAATCATGATGGGATATAAAGTGCAGTTTGTGATAATTAA TATTTTT |
|  |  |  |  |
| 843 | 877 | 26,437 | ATAT ATAAACGGTCAAATGTATTACTTATAAGATAGAG TTGATAATTTTTTTTTTTTTC |
| 834 | 877 | 1,279 | AT ATAAACGGTCAAATGTATTGCTTGTAAGGTAGAGGTGATAATT TTTTTTTCTTTTT |
| 829 | 865 | 188 | ATATATAACGATC AATGCATTATCTATGAAGCAAGAACAGTGATTATAAT TTTTTTTTTTTTTTT |
| 831 | 857 | 121 | AATGTGTG ACCTATAAAGTGAGAATAATGATTATA TTTTTTTTTTTT |
| 832 | 865 | 23 | ATATATAACGATC AATGCATTATCTATGAAGCAAGAACAGTGATTAT TTTT |
| 832 | 857 | 20 | AATGTGTG ACCTATAAAGTGAGAATAATGATTAT TTTTTTTTTTT |
| 834 | 879 | 1 | ATAT AAATAAACGATTAGATGTATCACTTATAGAATAAAAATGATGATT TTTTTTTTTTTTTTT |
|  |  |  |  |
| 863 | 902 | 2,491 | ATATA ATATGCATATCAGATAGACGTAGAAATAAGTGATTAAAT TTTTTTTTTT |
| 866 | 902 | 126 | ATATAATAA ATACGCATATTAGATGAGTGTAAAAGTAAGTGATTA TTTTTTTTTT |
|  |  |  |  |
| 872 | 916 | 3 | ATATAA AATCAACAAATTCGTATGTATATCGAGTAAATGTAGAAATAAAT TTTTTTTTT |
| 872 | 919 | 3,227 | A TACAAATCAACAAATTCGTGCGTATATTAAGTGGGTGTAGAAGTGAAT TTTTTTTTTTTTTTTTT* |
| 867 | 919 | 528 | A TACAAATCAACAAATTCGTGCGTATATTAAGTGGGTGTAGAAGTGAATGATT TTTTTTTATTTTTTT |
| 872 | 919 | 295 | A TACAAATCAACAAATTCGTGCGTATATTAAGTGAGTGTAGAAGTGAAT TAAATTTTTTTTTTTTTTTTTTT |
| 871 | 919 | 142 | A TACAAATCAACAAATTCGTGCGTATATTAAGTGGGTGTAGAAGTGAATG TTTTTTTTTTTTTTT |
|  |  |  |  |
| 901 | 939 | 1,365 | ATATAT ACTAATAAAAAGGCATTGCTTACAGATTGATAGATTTAT TTTTTTTTTTTT |
| 899 | 931 | 292 | ATATATTACCAACAT AGAGACATTGCTTATGAGTTAACAGATTTATAT TTTTTTTTTTTT |
| 901 | 939 | 249 | ATATAT ACTAACAAAAAGATATTGCTTATAGATCAGTGAATTTAT TTTTTTTTTTT |
|  |  |  |  |
| 907 | 947 | 24 | ATAAAGAAACCAACAGAAGAATATTGCTTGTAAGTTAATGA TCTTGTATTTTTTTTTT |
| 907 | 951 | 64 | CCAAGGCA AAAGATAAAGAAACCAACAGAAGAATATTGCTTGTGAGTTAATGA TCTTTTTTTTTTTTTTTTT |
|  |  |  |  |
| 932 | 978 | 44,852 | ATATAT TCAAACAAACAGATAGAACCGGAGACGAGAAGATTGATAA TTTTTTTTTTTTT |
| 934 | 988 | 11,706 | ATATAAATAATCAAACAGACGAATGAAACTAGAGATAGAGAAATTAAT TTTTTTTTTTTT |
| 931 | 978 | 3,513 | ATATAT TCAAACAAACAGATAGAACCAGAGACGAGAAGATTGATGAA TTTTTTTTTTTT |
| 933 | 986 | 2,467 | ATAAATAATCAAACAGACGAATGAAACTAGAGATAGAGAAATTAATA TTTTTTTTTTTT |
| 940 | 978 | 182 | ATATAT TCAAACAAACAGATAGAACCGGAGACGAGAAG CTTGATAATTTTT |
| 936 | 988 | 175 | ATATAAATAATCAAACAGACGAATGAAACTAGAGATAGAGAAATTA TTATTTTTTTTTTTTTTT |
| 941 | 978 | 137 | ATAT TCAAACAAACAGATAGAACCGGAGACGAGAA TATTGATAATTTAATTTTTTTTTTTTTT |
| 937 | 988 | 97 | ATATAAATAATCAAACAGACGAATGAAACTAGAGATAGAGAAATT TATTTTTTTTTTTTT |
| 934 | 983 | 18,961 | ATATAT AATAATCAAACAGACGAATGAAACTAGAGATAGAGAAATTAAT TTTTTTTTTTTT* |
| 933 | 983 | 1,729 | ATATAT AATAATCAAACAGACGAATGAAACTAGAGATAGAGAAATTAATA TTTTT |
| 936 | 983 | 472 | ATATAT AATAATCAAACAGACGAATGAAACTAGAGATAGAGAAATTA TTTTTTTTTTTTTT |
| 937 | 983 | 397 | ATATAT AATAATCAAACAGACGAATGAAACTAGAGATAGAGAAATT TTTTTTTTTAA |
|  |  |  |  |
| 959 | 1000 | 173,548 | ATAAA GGTAATATCACAGTGTAGATAGTCGGATAGATAGATGAAA ATTTTTTTTTTTTTT |
| 952 | 1000 | 748 | ATAAA GGTAATATCACAGTGTAGATAGTCGGATAGATAGATGAAATT TTTTTTTTT |
| 960 | 1000 | 472 | ATAAA GGTAATATCACAGTGTAGATAGTCGGATAGATAGATGAA TTTTTTTTGTTTT |
| 966 | 1000 | 412 | ATAAA GGTAATATCACAGTGTAGATAGTCGGATAGATA TATGAAAATTTTTTTTTTTTT |
| 964 | 1000 | 372 | ATAAA GGTAATATCACAGTGTAGATAGTCGGATAGATAGA AAAAATTTTTTATTTTTTT |
| 963 | 1000 | 371 | ATAAA GGTAATATCACAGTGTAGATAGTCGGATAGATAGAT TTTCTTTTTTTTTTT |
| 961 | 1000 | 265 | ATAAA GGTAATATCACAGTGTAGATAGTCGGATAGATAGATGA TTTTTTTTTTTT |
| 962 | 1000 | 234 | ATAAA GGTAATATCACAGTGTAGATAGTCGGATAGATAGATG TAAATTTATTTTTTTTT |
| 959 | 1000 | 3,928 | ATATAAA GGTAATATCACAGTGTAGATAGTCGGATAAATAGATGAAA ATTTTTTTTTTT |
| 5' | 3' | Reads | ND7 gRNA Sequences cont. |
| 983 | 1017 | 101 | ATATATAAATAACATAT TAATGGTCTTGATGGTGATATTGTAGTATAA TTTTTTTTTTTT |
|  |  |  |  |
| 1001 | 1032 | 3 | ATATA TATAAAATAACGTGATGATGGTCTCGAT AGTAATTGATAATTTTTTTT |
| 1000 | 1043 | 1 | ATAT ACACCACAAACTGTAGAATGACGTGATAGTGGTTTCAGTG ATAATTTTTTTTTTTTTTT |
|  |  |  |  |
| 1015 | 1043 | 12 | AT ACATCACAAACTATAAAGTAATATGATAA GGGTTTCGATTTTTTTTTTTTTAA |
| 1013 | 1043 | 208 | ATAT ACACCACAAACTGTAGAATGACGTGATAGTG ATTTTTTCTTTTTTTTTTT |
|  |  |  |  |
| 1032 | 1067 | 6,797 | ATATATACAAGC AATGATGTACTCGGTAAATAGTGACACTGTGAATT TTTTTTTTTTTC |
| 1030 | 1067 | 2,406 | ATATATACAAGC AATGATGTACTCGGTAAATAGTGACACTGTGAATTAT TTTTTTTTTTTT |
| 1032 | 1078 | 751 | A AATATAAGCAAATGATGTATTCGGTGAGCAGTGATATCGTAAATT TTTTTTTTTT |
|  |  |  |  |
| 1057 | 1085 | 44 | ATTAGAAA GGTGTTCGGTATAGGTAGATGATATAT ATTTTTTTTTT |
| 1055 | 1085 | 40 | CTCATTAGAAA GGTGTTCGGTATAGGTAGATGATATATTT TTT |
|  |  |  |  |
| 1089 | 1121 | 1 | AT ATATGATAACAAACAATACTTACTTT GAGGTGTTTTGTGAGTAAATTTTTTTTTTTTTT |
| 1087 | 1113 | 412 | ATA AATAACAAACAATATTCGTCTTTG AGATATTCGATATAAGTAAATTTTTTTTTTTT† |
|  |  |  |  |
| 1099 | 1143 | 81,287 | ATATAA GAGAACATAAACTAGCATAGAGGCATAGTAACAAGTGATAT ATTTTTTTTTTTTTT |
| 1094 | 1143 | 6,615 | ATATAA GAGAACATAAACTAGCATAGAGGCATAGTAACAAGTGATATTT TTTTTTTTTT |
| 1099 | 1142 | 5,793 | ATATAAA AGAACATAAACTGACACGAGGGTATAGTGATGAATGATAT ATTTTTTTTTTTTC |
| 1099 | 1131 | 705 | ATATAAGAGAACATAAA CAGCATAGAGGCATAGTAACAAGTGATAT ATTTTTTTTTTTTGT |
| 1101 | 1143 | 550 | ATATAA GAGAACATAAACTAGCATAGAGGCATAGTAACAAGTGAT TTTTTTTTGTTTT |
| 1105 | 1143 | 520 | TATAA GAGAACATAAACTAGCATAGAGGCATAGTAACAAG GGATATATTTTTTCTTTTTT |
| 1106 | 1143 | 318 | ATATAA GAGAACATAAACTAGCATAGAGGCATAGTAACAA TTTTTTTTTTT |
| 1108 | 1143 | 303 | ATATAA GAGAACATAAACTAGCATAGAGGCATAGTAAC TTTTTTTTTTT |
| 1094 | 1142 | 289 | ATATAAA AGAACATAAACTGACACGAGGGTATAGTGATGAATGATATTT TTTTGTTTTT |
| 1099 | 1143 | 259 | ATATAA GAGAACATAAACTAGCATAGAGGCATAGTAACAAGCGATAT ACTTTTTTTTTTTT |
| 1107 | 1143 | 177 | ATATAA GAGAACATAAACTAGCATAGAGGCATAGTAACA TTTTTTTTTTTG |
| 1099 | 1143 | 163 | ATATAA GAGAACATAAACTAGCATAGAAGCATAGTAACAAGTGATAT ATTTTT |
| 1099 | 1143 | 162 | ATATAA GAGAACATAAACTAGCATAGAGGCATAGTAACAAATGATAT ATTTTT |
| 1099 | 1143 | 128 | ATATAA GAGAACATAAACTAACATAGAGGCATAGTAACAAGTGATAT ATTTTTTTGTTTTTT |
| 1099 | 1143 | 98 | ATATAA GAGAACATAAACTAGCATAGAGACATAGTAACAAGTGATAT ATTTTTTTTTCTTTT |
| 1099 | 1143 | 356 | ATATAA GAGAACATAAATTGACATGGAAGCATAGTAATAAGTGATAT ATTTTTTTTTTTT* |
| 1095 | 1143 | 212 | ATATAA GAGAACATAAATCAGTGCAAAGGTATAGTAGTGAGTGATATT AATTTTTTTTT* |
|  |  |  |  |
| 1108 | 1145 | 569 | ATATAAACGTAT ACGAGAGCATAGATCAGTGTGAGAATGTAGTAAT TTTTTTTCTTTTTT |
| 1094 | 1148 | 295 | ATATAAAA TAAACGAGAATATAAACTGATGTAGAGATATAGTGATAAGTAATATTT TTTTTTTT |
| 1107 | 1157 | 82 | AT ATGTAAATGTAAACGAGAATATAGATTGATGTAGAGATATAGTAATA TTTTTTTTTTTTT |
| 1107 | 1146 | 90,559 | ATATTAAACGT AACGAGAATGTGAACTGACATAGAGATATGATAATA TTTTTTTTTTTTTT |
| 1108 | 1146 | 10,003 | ATATTAAACGT AACGAGAATGTGAACTGACATAGAGATATGATAAT TTTTCTTTTTTTTT |
| 1111 | 1146 | 2,224 | ATATTAAACGT AACGAGAATGTGAACTGACATAGAGATATGAT TTTTTTTTTG |
| 1114 | 1146 | 2,220 | ATATTAAACGT AACGAGAATGTGAACTGACATAGAGATAT TTTTTCTTTTTTTT |
| 1110 | 1146 | 1,887 | ATATTAAACGT AACGAGAATGTGAACTGACATAGAGATATGATA TTTTTATTTTTTTTTT |
| 1108 | 1154 | 978 | ATATA TAAACGTAAATGAGAATATGAATCAGTGTGAAAATGTAATAAT TTTTTTT* |
| 1107 | 1146 | 400 | ATTAAACGT AACGAGAATGTGAACTGACATAGAGATATGATAATG TTTTTTTTTTTTT |
| 1120 | 1146 | 266 | ATATTAAACGT AACGAGAATGTGAACTGACATAGAGAT TTTTTTTTTTTTT |
| 1113 | 1146 | 243 | ATATTAAACGT AACGAGAATGTGAACTGACATAGAGATATG TTTT |
| 1108 | 1152 | 232 | ATAT AACGTAAACGAGAGCATAAATTGATGTGAAGATGTGATAAT TTCTTTT |
|  |  |  |  |
| 1128 | 1167 | 3,079 | ATATAT AATCCGTACAATGCGAACGTAGACGAGAATATGAGTTAAC TTTTTTTTTATTTT |
| 1136 | 1182 | 1,834 | ATAT ATAAATATGCAAGAAATCTGTATGATGTAGATGTGAATGAGAATAT TTTTTTTTTT |
| 1138 | 1182 | 866 | ATAT ATAAATATGCAAGAAATCTGTATGATGTAGATGTGAATGAGAAT TTTTTTCTTTT |
| 1128 | 1167 | 337 | ATATAT AATCCGTACAATGCGAACGTAGACGAGAATATGAGTTAAT TTT |
| 1131 | 1167 | 203 | ATATAT AATCCGTACAATGCGAACGTAGACGAGAATATGAGTT TTCTTTTTTTTTTTTTTTT |
|  |  |  |  |
| 1150 | 1197 | 213 | AT ATAAACATCCAATAGACGAGTATGTGAGAGATTTGTATGATGTAAAT TTTTTTTTTT |
| 1150 | 1195 | 1,077 | AAATATCCAATAAACAGATATGTAGAAGGTCCGTATAATGTGAAT ATATTTTTTTTTTGTT |
|  |  |  |  |
| 1181 | 1218 | 4,103 | ATATATAA ATGCAATAGAAGATCACGCAAATAGATATCTGATAAAT TTTTTTTTTTTTC |
| 1183 | 1224 | 1,399 | ATA TAAATCATGCAGTAAAAGACTATGTAGATGGATATTCAGTGA TAATTTTTTTTTTTTTT |
| 1183 | 1223 | 1,017 | AAATA AAATCATGCAGTAGAGAACCGTGTAAGTGAGTATCTGATAA TTATTTTTTTTTTT |
| 1183 | 1224 | 138 | ATA TAAATCATGCAGTGAAGAGCTACGTAAATGGATATTCAGTGA TAATTTTTTTTTTT |
| 1183 | 1224 | 2,561 | ATA TAAATCATGCAGTAAAAGACTATGTAGATGAATATTCAGTGA TAATTTTTTTTTTTTTT* |
|  |  |  |  |
| 1210 | 1257 | 123 | ATAT ACAACATCAATATTACTTAGAACGGTAACTAGATTGTGTAATAA TTTTTTTTTTTTTT |
| 1233 | 1257 | 167 | ATAT ACAACATCAATATTACTTAGAACG ATAACTAGATTGTGTAATTTTTTTTTT† |
| 1233 | 1257 | 83 | ATAT ATAACATCAATATTACCTAGAGTG TCAGTTAGATTATGTGATAAATTTTTTTTTTTTTT† |
|  |  |  |  |
|  |  |  |  |

| 5' | 3' | Reads | ND7 gRNA Sequences cont. |
| --- | --- | --- | --- |
| 1240 | 1268 | 95 | AAACTAACGATATT CGGATCTGAGAGTAACATTGATATTATTT TTTTTTT |
| 1251 | 1282 | 63 | ATATATAT AACTAACGATCTATGGGTTTAAAGACAGTGT GAATTTTTTTTTTTT |
| 1242 | 1283 | 6 | AC AAACTAACGATTTACGGATTTAGAGACAGTGTTAATGTTAT ATTTTTTTTTTTTT |
| 1242 | 1270 | 296 | A TACGGATTCAGAAGTGATATTGATGTTAT ATTTTTTTTTTTTTTT |
| 1240 | 1268 | 216 | AAACTAACGATATT CGGATCTGAGAGTAACATTGATATTATTT T |
| 1240 | 1270 | 54 | ATATAAACTAACGA TACGGATTCAGAAGTGATATTGATGTTATTT TTTTTTTTTTTTTTTT |
| 1239 | 1268 | 25 | ATT CGGATCTGAGAGTAACATTGATATTATTTA TTTTTTTTTTTTTTTTT |
| 1240 | 1268 | 23 | ATT CGGATCTGAGAGTAACATTGATATTGTTT TT |
| 1241 | 1268 | 12 | GATATT CGGATCTGAGAGTAACATTGATATTATT ATTTTTTTTTTTTTTT |
|  |  |  |  |
| 1269 | 1320 | 6 | ATA TAAACAATCCTACAATGATCTCGTGTATAAGACTGATGATTTA ATTTTTTTTTTTT |
| 1269 | 1320 | 1,074 | ATA TAAACAATCCTACAATGATTTCGTGTATAAGACTGATGATTTA ATTTTTTTTTTTTTTTTT |

I) NADH Dehydrogenase 8

| 5' | 3' | Reads | ND8 gRNA Sequences |
| --- | --- | --- | --- |
| 34 | 58 | 2 | GTGGG ATATGAAAGTAAGAGAATAAAAAAA ATTAATTTTTTTTTTTTT |
| 29 | 68 | 1 | AA AAAAAAAAACATACAGAAATAGAAAGATAAGAAAGTGATA TATTATTTTTTTT |
| 28 | 56 | 9 | AAA ATAGGAGTAGGAGGATGAGAAAATGATAG AGGGATTTT* |
|  |  |  |  |
| 55 | 98 | 2,577 | ATAT AAAACAAACAAAAAGAAGAAACAAGAAATTGAAGAGAGATATAT TTTTTTTTTTTAT |
| 55 | 98 | 274 | ATAT AAAACAAACAAAAAGAAGAAGCGAGAAATTGAAGAGAGATATAT TTTTTTTCTTTT |
| 54 | 97 | 236 | ATAT AAACAAACAGAAAAAGAAAACAAAGAGTCAGAGAAAGATATATA TTTTTTTATTTTTTTTT |
| 55 | 97 | 100 | ATAT AAACAAACAGAAAAAGAAAACAAAGAGTCAGAGAAAGATATAT TTTTTTTTTT |
| 57 | 98 | 98 | ATAT AAAACAAACAAAAAGAAGAAGCGAGAAATTGAAGAGAGATAT TTTTT |
| 57 | 97 | 65 | ATAT AAACAAACAGAAAAAGAAAACAAAGAGTCAGAGAAAGATAT TTTTT |
| 54 | 97 | 57 | ATAT AAACAGACAGAAAAAGAAAACAAAGAGTCAGAGAAAGATATATA TGTAATTATTTT* |
| 59 | 97 | 29 | ATAT AAACAAACAGAAAAAGAAAACAAAGAGTCAGAGAAAGAT TTTTTTTTT |
|  |  |  |  |
| 87 | 136 | 6,039 | ATAAATAGTAACACAATGAGCAGAGTACGTATAAGAATGAGTAAA TTTTTTTTTTTTTT |
| 84 | 133 | 633 | A AAATAGTAATACAACAGACAGAGCATATATAGAAATAAGTGAGAAA TTTTTTTTATTTTTTT |
| 87 | 133 | 593 | AAATAGTAACACAATGAGCAGAGTACGTATAAGAATGAGTAAA TTTTTTTCTTTTTTT |
| 87 | 135 | 394 | TAAATAGTAACACAATGAGCAGAGTACGTATAAGAATGAGTAAA TTTTTTTTTTTT |
| 84 | 131 | 380 | AT ATAGTAATACAACAAACGAGATACGTATAGAAATAGATGAGAAA TTTTTTTTTTTTT |
| 87 | 136 | 292 | GTAAATAGTAACACAATGAGCAGAGTACGTATAAGAATGAGTAAA TTTTTTTTTTGT |
| 96 | 136 | 191 | ATAAATAGTAACACAATAGACGAGATACGTGTAGAA TAAGTGATTTAATTTTTTTTTTT |
| 86 | 139 | 11,689 | ATA TAAACAAATAGTAACATGACGGATAGAACGTATATGAGAATGAGTAAAA TTTTTTTTTTTT* |
| 85 | 139 | 1,938 | ATA TAAACAAATAGTAACATGACGGATAGAACGTATATGAGAATGAGTAAAAG TTTTTTTTCTTTT |
| 86 | 139 | 1,171 | A TAAACAAATAGTAATATGACGAATGAAGCGTATATGAGAATAAGTAAAA TTTT* |
| 87 | 139 | 816 | ATA TAAACAAATAGTAACATGACGGATAGAACGTATATGAGAATGAGTAAA TTTATTTTTTTTTTTTTT |
| 88 | 132 | 612 | ATAT AATAGTAACACAACGAATAGAACATGTATAGAGATGAATGA TATTTTTTTTTTTTTTTTT |
| 84 | 131 | 598 | ATAT ATAGTAACACAATAAATGAGACATATATGAAGATGAATGAGAAA TTTT* |
| 92 | 133 | 267 | ATATA GAATAGTAACACAGCAGATAAGATACATATAGAGATAA TGACAGTTTTTTT |
| 92 | 132 | 267 | ATATAT AATAGTAACACAGCAGATAAGATACATATAGAGATAA TGACAGTTTTTTTCTTTTTT |
| 86 | 138 | 221 | AA AAACAAATAGTAATATGACGAATGAAGCGTATATGAGAATAAGTAAAA TTTTTT |
| 85 | 138 | 196 | AA AAACAAATAGTAATATGACGAATGAAGCGTATATGAGAATAAGTAAAAA TTTTTTTTTT |
|  |  |  |  |
| 111 | 153 | 70,659 | ATATATAAATGGTA AACTCAATGGGTGGATAAGTAGTAATGTGATGAAT TTTTTTTTTTTTT |
| 111 | 153 | 479 | ATATATAAATGGTA AACTCAATGGGTGGATAAATAGTAATGTGATGAAT TTT |
| 111 | 153 | 245 | ATATATAAATGGTA AACTCAATGGGTGGATAAGTAGTAATGTGATAAAT TTTTTTTCTTTT |
| 111 | 153 | 147 | ATATATAAATGGTA AACTCAATGGGTGGATAAGTAGTAATATGATGAAT TGTAATATTTTTTTTTTTTTTT |
| 115 | 153 | 127 | ATATATAAATGGTA AACTCAATGGGTGGATAAGTAGTAATGTGAT TTTTTTTATTTTTT |
| 113 | 153 | 121 | ATATATAAATGGTA AACTCAATGGGTGGATAAGTAGTAATGTGATGA TTTTTTTTTCTTTTT |
| 111 | 152 | 804 | A TATACAATGGTT ATTCAGTGGGTAGACAGATAGTGATATGATAGAC TTATTTTTTTTTTTT |
|  |  |  |  |
| 117 | 170 | 4 | ACAT ATAAACTAACAATGGTTGATTTAGTGAGTGAATGAGTAGTAATATT TTTTTTTTTTT |
|  |  |  |  |
| 158 | 186 | 169,806 | ATAT GAACGCAAAGATGGATTACCACGAGTTAGTAAATTGATGAT ATTTTTTTTTTTTTT |
| 161 | 187 | 124,070 | ATATAAT AAACGCAAAATATGGTTACTATGAACTGATAGATTAAT TTTTTTTTTTTC |
| 161 | 187 | 799 | AAACGCAAAATATGGTTACTATGAACTGATAGATTAAT TTTTTTTTTTT |
| 161 | 198 | 66 | ATATAAT AAACGCAAAAAATGGTTACTATGAACTGATAGATTAAT TTTTTTTTGTTTTT |
| 161 | 207 | 2,257 | ATATA TAATAAAGACGCAAAAGATGGTTACTGTGAATTGATGAGTTAAT TTTTTTTTTTTT* |
| 161 | 199 | 966 | ATATATAGT AAAACGCAGAAAATGGTTACTATGGATTGATGAATTAAT TTTTTTTTTTTTTTTT |
| 160 | 199 | 804 | ATATAGT AAAACGCAGAAAATGGTTACTATGGATTGATGAATTAATG TTTTTTTTTTTTTTT* |
|  |  |  |  |
| 186 | 230 | 24,763 | ATA CAATACAACGCTCTGAATCATATCGATAAAAGTGTGAGAAAT TTTTTTTTTTTTT |
| 186 | 229 | 182 | AATACAACGCTCTGAATCATATCGATAAAAGTGTGAGAAAT TTAATTTTTTTATTTT |
| 186 | 230 | 109 | ATA CAATACAACGCTCTGAATCATATCGATAAAAGCGTGAGAAAT TTTTTTTTTTT |
| 186 | 220 | 204 | ATA CAATACAACACTCTGAATCATATCGATAAAAGTGTGAGAAAT TTAATTTTTTTTTT |
| 194 | 230 | 160 | ATA CAATACAACGCTCTGAATCATATCGATAAAAGTG GGAGAAATTTTTTTTTTG |
| 195 | 230 | 149 | ATA CAATACAACGCTCTGAATCATATCGATAAAAGT TGAGAAATTTAATTTTTTTTTTT |
| 196 | 230 | 199 | ATA CAATACAACGCTCTGAATCATATCGATAAAAG AGTGAGAAATTTAATTTTT |
| 186 | 228 | 161 | T ATACAACGCTCTAAATTATACCAGTGAAAATGCGAGAAAT TTTTTTTTTTTTTT |
| 187 | 228 | 52 | ATATTAT ATACAACGCTCTAAATTATACCAGTGAAAATGCGAGAAA ATTTTTTTTTTT* |
| 189 | 229 | 31 | ATATA AATACAACGCTTTAGATCATATCAGTGAGAGTGTGAAA TTTTTTTTTTTTT |
|  |  |  |  |
| 213 | 244 | 173 | A ACATAAACGACAGGTAATATGATGTTCTGAAT GATATCGATAATTTTTC |
| 209 | 239 | 19 | ACAT AACGACAAGTGATATAACGTTTTAAGTTACA GTGATAATTTTTTTTTTTTTTTTTTT |
| 219 | 245 | 10 | AAATA TACATAAACGATGAGTAATATGACGTT GTAGATTTTTTTTTTTTT |
| 219 | 254 | 1 | AAATTAAATCACATAAATGACGAGCGATACAGTGCT GTAGATGATACTTTTTT |
|  |  |  |  |
|  |  |  |  |
|  |  |  |  |
|  |  |  |  |
|  |  |  |  |
| 5' | 3' | Reads | ND8 gRNA Sequences cont. |
| 240 | 288 | 3 | TATGAACATCCGATACTGAACTAGGGTAGATTGAGTTATATA TTTTTTTTTT |
| 237 | 267 | 2 | CATCCAATGTAT AATTAGGGTAGATTGAGTTATGTAAAT TTTTTTTTTTTTTTT |
| 246 | 271 | 340 | ATACGAACATCCATA GTTAGATTAGGGTAAATTGAGT GATGTAATTTTTTTGTTTT† |
| 259 | 285 | 325 | ATATAA GAACATCCGATGCTAGATTA TGGTAGATTGAGTTATATGATTGTAATATTTTT† |
| 270 | 291 | 268 | ATATA TAACACGAACATCTGATGT ATAAACTGAAGTAAATTGATTTTTTTTTTTTTTTT† |
|  |  |  |  |
| 276 | 318 | 51,820 | ATATAC AACGATGATCACTGAGATTTTACCTAATATGGATGTT AATTTTTTTTTTTTT* |
| 276 | 319 | 28,492 | ATATAT AAACGATGACTACTAGAATTCTACTCAATGTGAATGTT AATTTTTTTTTTTTTT |
| 277 | 317 | 1,829 | ATATAT ACGATGACTACCAAAATTCTATCTGATATGAATGT GATAATATTTTTTTTTTT |
| 279 | 318 | 594 | ATATAC AACGATGATCACTGAGATTTTACCTAATATGGAT TTTTTTTTATTTTTTTT |
| 275 | 318 | 384 | ATATAC AACGATGATCACTGAGATTTTACCTAATATGGATGTTT TTTTTTTTT |
| 279 | 319 | 334 | ATATAT AAACGATGACTACTAGAATTCTACTCAATGTGAAT TTTTTTCTTTTTTTT |
| 276 | 314 | 215 | GATGACTACTAGAATTCTACTCAATGTGAATGTT AATTTATGATATTTTTTTTTTTTTT |
| 276 | 311 | 186 | ATATACAACT ATGATCACTGAGATTTTACCTAATATGGATGTT AATTTTTTTTTTT |
| 284 | 321 | 140 | ATATATA CAAAACGATGATTACCGAGATTTCATTTAATATGA TTGTCTAATTTT |
| 287 | 319 | 139 | ATATAT AAACGATGACTACTAGAATTCTACTCAATG AATGTTAATTTT |
| 275 | 319 | 109 | ATATAT AAACGATGACTACTAGAATTCTACTCAATGTGAATGTTT TTTTTGTTTTTT |
| 289 | 318 | 4,761 | ATAATAT AACGATGACTATCAGAACTTTACTCGA GATGAATGTTTTTTTTTTTTT |
| 276 | 318 | 1,264 | ATATAT AACGATGACTACTGAGACTCTATCTGACGTGAATGTT AATTTTTTTTTTTT |
| 275 | 318 | 175 | ATATAT AACGATGACTACTGAGACTCTATCTGACGTGAATGTTT TTTTTTT* |
|  |  |  |  |
| 310 | 353 | 13,915 | ATATTA GATGATAACTCAGTGTAGATTGATCTGTAGAATGAT AATATTTTTTTTTTTTT |
| 301 | 344 | 636 | ATAT ATAACTCAATGTAGATCGATTTGTGAGATGATGATTGTCAA TTTTTTTTTTTATC |
| 310 | 350 | 312 | ATGATAACTCAGTGTAGATTGATCTGTAGAATGAT AATATATAATGTTTTT |
| 316 | 344 | 266 | ATATACA ATAACTCAATGTGAATTAATCTGTGAGAC TATTTTTTTTTTTTTT |
| 301 | 344 | 4 | AT ATAACTCAATGTAGATCGATCCGTAGAATGATGATTGCTAA TTTTTTTTTTT |
|  |  |  |  |
| 331 | 364 | 1,709 | ATAT ATAAATACAACGGTGATAATTCGATGTGA TGATATCTGTAATTTTTTTTTTTTTTAT |
| 325 | 358 | 137 | ATAA ATAACGACGATGATTCAGTGTAAATTGGT ATGTGAAATGATGTTTTTTTTTTTT |
| 325 | 355 | 21 | ACGACGATAGCTTGATGTAGGTTAGT GTGTGAGATAATGATATTTTTTTTTTTTTTTTT |
|  |  |  |  |
| 338 | 382 | 1,964 | ATATAA ATGCATACAAGAATCATAGTAAGTACAGTGATGATAATTT TTTTTTTTT |
| 350 | 386 | 1,269 | ATATA AAACATGTATACAAAAATTACAGTAAATGCGACGA AAATAATTTTTTTTTTTTT |
| 339 | 382 | 1,073 | ATATAA ATGCATACAAGAATCATAGTAAGTACAGTGATGATAATT ATTTTTTTTGTTTTT |
| 341 | 373 | 13,719 | ATATATAAATGCATAC AAGGCCATGATAGATACAATGATGATAA ATTTTTTTTTTT |
| 338 | 385 | 2,361 | ATGTAC AACATGCATACAGAGACTATAATAGATACAGTGATGATAATTT TTATTTT |
| 342 | 385 | 159 | ATGTAC AACATGCATACAGAGACTATAATAGATACAGTGATGATA TTTTGTTTTTTTTTTTTTT |
| 343 | 385 | 133 | ATGTAC AACATGCATACAGAGACTATAATAGATACAGTGATGAT TTTTTTT |
|  |  |  |  |
| 372 | 417 | 2 | ATAT ATAAATGCGTAATGGTATCTTTCGGGTAGATATGTGTATAAA TTTTTTTTTTTTTT |
| 390 | 414 | 894 | AAATTGTATATAT AATGCGTAATGGTGTTTGTTG AGCGAGTGTGTGTTATTTTTTTTTTTTTTT† |
|  |  |  |  |
| 391 | 431 | 23 | AT ATATATAACAAACAATGGGTGTATAATGGTATCTGAT TGTGCATTAATTTAAAATTTTTTTTTTTTTT |
| 405 | 434 | 9 | AT AAAACACATAATAAATGATGAGTGCGTGAT ATAGGTATAATTTTTTTTTTTTT |
|  |  |  |  |
| 411 | 442 | 1,950 | AAAAA AAACAACAAGAACACATAGCAGATAGTGGGTG ACGTATATTTTTTTTTTTC |
| 411 | 443 | 923 | A TAAACAACAAGAACACATAGCAGATAGTGGGTG ACGTATATGATTTTTTTTTTTTT |
| 407 | 441 | 215 | AAAT AACAACAAAAACATATAATAAATGATGGATGTGTA TAAGGTATATTTTTTTTTTTTTTT* |
|  |  |  |  |
| 426 | 466 | 24,242 | ATAAAACTTGGA CGCTAATAGATATATGGTTAGATAATGAGAATATAT TTTTTTTTTTTTC |
| 425 | 466 | 5,218 | ATAAAACTTGGA CGCTAATAGATATATGGTTAGATAATGAGAATATATA TTTTTTTTTTTTTT |
| 423 | 466 | 2,651 | ATAAAACTTGGA CGCTAATAGATATATGGTTAGATAATGAGAATATATAGT TAATTTTTTTTTTT |
| 428 | 466 | 1,071 | ATAAAACTTGGA CGCTAATAGATATATGGTTAGATAATGAGAATAT TTTTTTTTTT |
| 423 | 466 | 203 | TAAAACTTGGA CGCTAATAGATATATGGTTAGATAATGAGAATATATGGT TAATTTTTTTTTTTTTT |
| 426 | 482 | 13 | AT AAAACTTGGGCGCTAATAGATATATGGTTAGATAATGAGAATATAT TTTTGTTTTTTT |
| 423 | 462 | 29,495 | ATACTTGGGCACA CAATAGATATATGGCTGAATAATAGAGATATATAAT TTTTGTTTTTATT |
| 426 | 462 | 11,472 | ATACTTGGGCACA CAATAGATATATGGCTGAATAATAGAGATATAT TTTTCTTTTTTTTTTTTT |
| 425 | 462 | 7,969 | ATACTTGGGCACA CAATAGATATATGGCTGAATAATAGAGATATATA TTTTCTTTTTTT |
| 428 | 462 | 4,922 | ATACTTGGGCACA CAATAGATATATGGCTGAATAATAGAGATAT TCTTTT |
| 430 | 462 | 582 | ATACTTGGGCACA CAATAGATATATGGCTGAATAATAGAGAT TTTTGTTTTTTTTT |
| 426 | 464 | 526 | ATATATAACTTGGGCA TCAATGAGTATATGGTTAAGTGATGAAGATATAT TTTTTTTTTT |
| 426 | 480 | 127 | ATATAT AACTTGGGCGTCAATGAGTATATGGTTAAGTGATGAAGATATAT TTT |
| 427 | 462 | 111 | ATACTTGGGCACA CAATAGATATATGGCTGAATAATAGAGATATA ATCTTTT |
|  |  |  |  |
| 465 | 503 | 1 | ATAT TAAAACAACAATCAAATGATAGAAGCTTGGGTG ACTGTGAATATTTT |
|  |  |  |  |
| 477 | 512 | 176 | ATATA TAAATAACATAAGACGATAACTGAATAATAGAAATT AAGTGTCAATTTTTTTTTTTTTT |
| 482 | 510 | 832 | ATATATT AATAACATAGAGCAATGACCGAGTAATGA TATTTTTTTTCTTT |
| 477 | 512 | 237 | ATATA TAAATAACATAAAACGATAACTGAATGATAGAGATT AAGTGTCAATTTTTTTTT |
|  |  |  |  |
| 489 | 531 | 21,576 | ATAG ATAAAACACAAATAAAGGTCAAGTGATATAGAGTGATGATTAA TTTTTTTTTTTTAT |
| 489 | 528 | 5,835 | ATAAATAT AAACACAAATAAAGGTCAAGTAATGTAGAGTGATAATTAA TTTTTTTTTTTT |
| 5' | 3' | Reads | ND8 gRNA Sequences cont. |
| 500 | 540 | 2,861 | ATATAT AATAACTACACGAGACATGAATAGAAATTAAGTGATGTAAA TTTTTTTTTTTT |
| 494 | 536 | 1,242 | ATATAT ACTACATAAAACACAGATAAGAATCAGATAGTGTGAGATAATA TTTTTTTTTGTTTTT |
| 495 | 539 | 884 | ATAT ATAACTACACAAAATATAGGTAAAAGTTAGATGATGTGAAATGAT TTTTTTTTTTT |
| 495 | 536 | 151 | ATATAT ACTACATAAAACACAGATAAGAATCAGATAGTGTGAGATAAT TTTTCTTTTTTTTTT |
| 494 | 539 | 1,101 | ATAT ATAACTACACAGAACATAGATAAGAGTCAGATAGTATAAAGTGATA TTTTTTTTTTTTTTTTTTT |
| 495 | 539 | 405 | ATAT ATAACTACACAGAACATAGATAAGAGTCAGATAGTATAAAGTGAT TTTTTTTTTTTTTTT* |
| 500 | 541 | 198 | ATA AAATAACTACACAGAATACGAGTAAAGATTGAATGATGTAAA TAATTTTTTTTTTTTTTTTTT |
| 495 | 539 | 127 | ATAT ATAACTACACAGAGCACAAATGAAAGTTAAGTAATGTGAAATAGT TTTTCTTTTTTTTTTTTTTTTTG |
|  |  |  |  |
| 523 | 567 | 413 | ATAAA TATAAACACAGTAAATCACTCGAGATAGATAGTTATATGAGATAT TTTTTTTTTTT |
|  |  |  |  |
| 554 | 598 | 20 | ATATA TAATTTCACCGTGAATTTCTTTAGATTGTAGATATGATAA TTTTTTTTTTTTT |
| 554 | 598 | 7 | ATATA TAATTTCACCGTGGATTTCTTTAGATTATAGATATGATAA TTTTTT |
| 554 | 598 | 5,311 | ATATA TAATTTCACCGTGGATTTCTTTAGATTGTAGATATGATAA TTTTT |
| 554 | 598 | 386 | ATATA TAATTTCACCGTGGATTTCTTTAGATTGTAGATATGGTAA TTTTTTTTTTTTTTT* |

J) NADH Dehydrogenase 9

| 5' | 3' | Reads | ND9 gRNA Sequences |
| --- | --- | --- | --- |
| 33 | 71 | 18 | ATAT AAACATAAACGAAATGAGCATAGAAGTATATGTATGATG ATATTATTTTTTTTTTTTTT |
| 29 | 72 | 93 | ATAT AAAACATAAACGGAATAAATATAAGAGTGTATATGTGATATAAT TTTTTTTGTTTTTTT |
| 25 | 72 | 32 | ATAT AAAACATAAACGGAATAAATATAAGAGTGTATATGTGATATAATGTTT TTTTTTTTTTT |
|  |  |  |  |
| 60 | 105 | 540 | A TATAACACAAACAATAGAATAAAGTTAAGTGAGAATATGAGTGA TTTATTTTTTTTTTT |
| 60 | 101 | 472 | ATAT ATACAAACAATAAAATAGAATTAGATAGAGGTATAAGTGA TGTAAATTTTTTTTTTT |
|  |  |  |  |
| 87 | 124 | 1 | ATATCAAC AAACAAATAGAGCACTGTCTATGATATAAGTGATAA TTCTTTTTTTTT |
| 87 | 124 | 536 | AAATATCAAC AAACAGACGAGACATCATCTACAGTATAAGTGATAA TATTTTTTTTTT |
| 87 | 124 | 453 | AAATATCAAC AAACAAATAGAGCACTGTCTACGATATAAGTGATAA TTTTT |
|  |  |  |  |
| 117 | 160 | 1,145 | ATAT ATAAAACAATAAAGAAATAGAAGGCTACAGTTAATGAGATAAAT TTTTTTTTTTTTT |
| 130 | 167 | 137 | AAAATTAACAAAACAATAAAAGAACGAGAAATTACAGT GAATGAAGTAAATTTTTTT |
| 117 | 162 | 906 | ATA TAACAAAACAATAAAGAAATGAGAAACTGTGATTGATAAGATAAAT TTTTTTTTTTTT* |
| 119 | 162 | 65 | ATATA TAACAAAACAATAAGAAGACAGAGAGTTACAGTTAATAAGATAA TTATTTTTTTTTTTTTTTTTTTGGA |
| 116 | 162 | 55 | ATA TAACAAAACAATAAAGAAATGAGAAACTGTGATTGATAAGATAAATA TTTTT |
| 121 | 162 | 30 | ATA TAACAAAACAATAAAGAAATGAGAAACTGTGATTGATAAGAT TTTTTTTTTTT |
|  |  |  |  |
| 149 | 193 | 1,196 | ATAT AATAAAAACATACAATAAAATAGAAGGAAACTAATAAGATGATAG TTTTTTTTTTTTTTT |
| 147 | 187 | 5,984 | ATATAT AACATACAATAAGACGAAGAGAAACTAATAGAGTGATAAGA TTTTTTTTTTTTTTT* |
| 150 | 193 | 463 | ATAT AATAAAAACATACAATAGAATGAAAGGAAACTAATAAGATGATA TATTTTTTTTTTT |
|  |  |  |  |
| 176 | 216 | 3,869 | ATACAATAT AAAACAAAAGTCACAGATTAAGGGATAGAGATGTGTGATAA TTTTTTTTTTTTTT |
| 177 | 216 | 357 | ATACAATAT AAAACAAAAGTCACAGATTAAGGGATAGAGATGTGTGATA TTTTTTTTTTT |
| 176 | 216 | 620 | ATACAATAT AAAACAAAAATCGTAGATTAAAAGATAGAGATATATGATAA TTTTTTTTTTTTTT |
| 176 | 221 | 63 | ATAT ATATAAAAACAAAAGTCACGAATTAGAAAGTAAAGATATGTGATAA TTTT |
|  |  |  |  |
| 201 | 242 | 87 | ATATAA AATCAATAACAGATCATGATGATATAGAGATAGAAGTTATAA TTTTTTTGTTTTTTT |
| 204 | 249 | 652 | AAA AAAAATCAATCAATGATAGATCACAGTGGTATAGGGATGAGAATTA ATTTTTTTTTTT |
| 205 | 249 | 485 | AAAA AAAAATCAATCAATGATAGATCACAGTGGTATAGGGATGAGAATT TTCTTTTTTTT |
| 202 | 248 | 101 | ATAT AAAATCAATCAATAGCAAGTTACGATAATATAGAAGTGAGAATTATA TTTTTTTTTTTTT |
| 203 | 248 | 94 | ATAT AAAATCAATCAATAGCAAGTTACGATAATATAGAAGTGAGAATTAT TTTTTTTTTTTTT |
| 205 | 248 | 77 | ATAT AAAATCAATCAATAGCAAGTTACGATAATATAGAAGTGAGAATT TTTTTTTTTT* |
| 203 | 249 | 58 | AAAAA AAAAATCAATCAATGATAGATCACAGTGGTATAGGGATGAGAATTAT TTTTT |
|  |  |  |  |
| 239 | 279 | 569 | AAATTAT ACAACATAAGACGACGAAGATAAAGGTCATAGAGATTAATT TTTTTCTTTTTT |
| 239 | 268 | 561 | AACATAAAT CGGTGAAGATAGAGACTATGAGAATTAATT TTTTTTT |
| 240 | 268 | 346 | ATAAAT CGGTGAAGATAGAGACTATGAGAATTAAT ATGTTTTTTTTTTTTTTT |
| 238 | 279 | 86 | AAAATTAT ACAACATAAGACGACGAAGATAAAGGTCATAGAGATTAATTA TTTTTTTTTTT |
| 239 | 288 | 23 | AT AAATATACAACAATATAGAACGATGAAAGTGAAGATTATAAGAGTTAATT TTTTTCTTTTTT |
| 239 | 286 | 26,097 | ATATATAACAACATAGAACGATGAGAATAGAGATCATGAAAGTTAATT TTTTTTTTTTTATA |
| 239 | 286 | 7,153 | ATAC ATATACAACAATATAGAACGATGAAAGTGAAGATTATAAGAGTTAATT TTTTTTTTTTT |
| 238 | 286 | 1,828 | ATATATAACAACATAGAACGATGAGAATAGAGATCATGAAAGTTAATTA TTTTTTTTTTT |
| 243 | 286 | 1,812 | ATATATAACAACATAGAACGATGAGAATAGAGATCATGAAAGTT TTTTTTTTTT |
| 239 | 279 | 553 | AAATTAT ACAATATAAGACGACGAAGATAAAGGTCATAGAGATTAATT TTTTTTTTTT* |
| 242 | 286 | 499 | ATAC ATATACAACAATATAGAACGATGAAAGTGAAGATTATAAGAGTTA TTTTTTTTTTT |
| 247 | 289 | 451 | AAAATATACAACAATATGAAGCGACGAAAATAGAGACTATAGA TATTATTTTTT |
| 239 | 268 | 300 | ATAAAT CGATGAAGATAGAGACTATGAGAATTAATT TTGTTTTTTTTTT* |
| 246 | 286 | 262 | ATATATAACAACATAGAACGATGAGAATAGAGATCATGAAA TTTTTTATTTT |
| 243 | 286 | 236 | ATAC ATATACAACAATATAGAACGATGAAAGTGAAGATTATAAGAGTT TTTATTTTTTTTTTTTT |
| 236 | 282 | 197 | ACAACAATATAGAACGATGAAAGTGAAGATTATAAGAGTTAATTAAT TTTTTTTTTTTTT |
|  |  |  |  |
| 272 | 312 | 43 | ATAA GAACACACAGAGACAAGCAGAGTAAAGTGTATAGTGACATA TTTTTT |
| 273 | 314 | 5,869 | ATATAT ACGAACACACAGAAATAAGCAAGGTAGAATATATGATGATAT TTTTTTTTTTTTT* |
| 271 | 314 | 250 | ATAT ATGAACACACAGAAACAGATGAGATAGAGTATACAGTGATATAA TTTTTTTTTTTTTTTTT* |
| 275 | 314 | 157 | ATATAT ACGAACACACAGAAATAAGCAAGGTAGAATATATGATGAT TTTTTTTTTTTT |
| 272 | 314 | 93 | ATAT ATGAACACACAGAAACAGATGAGATAGAGTATACAGTGATATA TTTTTTTTTTTTTT |
|  |  |  |  |
| 303 | 339 | 44,686 | ATAT ACAAACAACACAAGATAGAGCACAAATGAATGCACAG TGATAATTTTTTTTTTTTT |
| 298 | 343 | 2,681 | ATAAACAAACAACACAGAGCAGAATACAAGTGAGTATATGAGAATA TTTTTTTTTTT |
| 298 | 343 | 1,741 | GTAAACAAACAACACAGAGCAGAATACAAGTGAGTATATGAGAATA TTTTTTTTTTTGT |
| 297 | 339 | 270 | ATAT ACAAACAACACAAGATAGAGCACAAATGAATGCACAGGGATAA TATTTTTTTTTTT |
| 304 | 339 | 139 | ATAT ACAAACAACACAAGATAGAGCACAAATGAATGCACA CTGATAATTTTTTTTTTTTT |
| 305 | 339 | 6,792 | ATAT ACAAACAACACAAGATAGAGCACAAATGAATGCAC TGAGATAATTTTTTTTTTTTTTG |
| 301 | 338 | 260 | ATATAT TAAACAACACGAAACGAAGCATAGATGGATATATGAGA TTCTTTT |
|  |  |  |  |
| 319 | 366 | 36 | AT ATTAAAACACAATCCGAAGAGTACAAATAGACAGTATAAGATAAAAT TTTTTTTTTTT |
| 319 | 366 | 17 | AT ATTAAAACACAATCCGAAGAGTATAAATAGACAGTATAAGATAAAAT TAATTTTTTTTTTTTTTTTTTT |
| 343 | 368 | 157 | AT AAATTAAAACACAATCTAAGAAATA GAGATAGACAGTATAAGATAAAATTTTTTTT† |
| 343 | 369 | 45 | AAAATTAAAACACAATCTAAGAAATA GAGATAGACAGTATAAGATAAAATTTTTTTT† |
|  |  |  |  |
| 5' | 3' | Reads | ND9 gRNA Sequences cont. |
| 352 | 392 | 10,040 | ATATT AAACGCATAACAGAAATGATTAGAACTGAGATATGATT AATTTTTTTTTTTTTC |
| 349 | 392 | 270 | ATATAT AGACGCATAATAAAAGCAACTGAGGCTAGAATATGATTTAA TTTTTTTGTTTTTTTTTTT |
| 349 | 392 | 232 | TAT AAACGTATAACAAAAGCAGTTAAAGCTAGAATGTGATTTAA TTTTTTTTATTTTT |
| 352 | 392 | 5,765 | ATATT AAACGCATAACAGAGACAATTAGAACTGAGATATGATT AATTTTTTTTTT* |
| 349 | 392 | 2,338 | ATATAT AAACGCATAATAAGGGCAACTGAAACTAGAGTATGATTTAA TTTCTTTTTTTTTTTT |
| 351 | 392 | 691 | ATATT AAACGCATAACAGAGACAATTAGAACTGAGATATGATTT TTTTCTTTTTTTTTTTTTT |
| 356 | 392 | 575 | ATATT AAACGCATAACAGAGACAATTAGAACTGAGATAT TTT |
| 351 | 392 | 189 | ATAT AAACGCATAATAAGGGCAACTGAAACTAGAGTATGATTT TTTTCTTTTTTTTTTT |
| 358 | 392 | 167 | ATATT AAACGCATAACAGAGACAATTAGAACTGAGAT TTTCCTTTTTT |
|  |  |  |  |
| 382 | 421 | 1 | AATCAAAATATTCGTGTTCTGATGATAGAGATGTATAAT TTTTTTTTTT |
| 380 | 418 | 317 | ATATA TAAAACATTCGCGTTCTAATGATAGAGATGTATGATAA TTTTTTTTTTTT |
|  |  |  |  |
| 409 | 447 | 761 | ATATAAA ATTACCAACAGAATAGAAGCTAGACAAGTTAAGATATTT TTTTTTTT |
| 409 | 447 | 667 | ATATAAA ATTACCAACAGAATAGAAGCTAGACAAGTTAAGATATTC TTTTTTTTTTTT |
| 410 | 447 | 82 | ATATAAA ATTACCAACGAAATAAAGACTAAGTGAATTAAGATGTT ATATTTTT |
|  |  |  |  |
| 439 | 484 | 72 | ATAT AAACCAATCAACGAGTAAATGATGTAGAGTATTATTGTCAAT TTTTTTTTATTTT |
| 438 | 483 | 185 | ATATAT AACCAATCAATAAGTGAGCGATGTAAGATGTTATTGTTAATA TTTTTTTTTT |
| 437 | 472 | 130 | ATATA TAACAAATAAACGATGTGAGATATCATTACCAGTGA TTTAATTTTTTTT |
| 438 | 483 | 46 | ATATAT AACCAATCAATAAGTGAGCGGTGTAAGATGTTATTGTTAATA TTTTTTTTTTTTTT |
| 439 | 483 | 34 | ATATAT AACCAATCAATAAGTGAGCGATGTAAGATGTTATTGTTAAT TTTTTTTTTTTTT |
| 447 | 472 | 16 | ATATA TAACAAATAAACGGTGTAGAGTGTCA GTATTTTTTTTTTTTTTTT |
| 442 | 483 | 25 | ATATAT AACCAATCAATAAGTGAGCGATGTAAGATGTTATTGTT TTTTTTTTTTTT |
|  |  |  |  |
| 468 | 514 | 234 | ATAT ATAACACTTCAACGGAGAGAGACCAATGAGAGATCAGTTAATA TTTTTTTGTTTTT |
| 469 | 514 | 2,106 | ATAT ATAACACTTCAACGAGAAGAAGCCAGTGAGAAATCAGTTAAT TTTTTTTTTTT |
| 469 | 514 | 1,535 | ATAT ATAACACTTCAATAGAAAGAAACTGACAGAGAATTGATTAAT TTTTTTTTTTTTG |
| 472 | 514 | 59 | ATAT ATAACACTTCAACGAGAAGAAGCCAGTGAGAAATCAGTT TTTAATTGTTTTTTTTTTTTTT |
|  |  |  |  |
| 502 | 549 | 746 | ATATAAAATAACAATACAAGTGAATCAGATAATGGATGATGTTTCAAT TTTTTTTTTTTTG |
| 509 | 542 | 1,407 | ATAT ATAACAATACAAATGAACTGAGTAATGGATAGTA GTTTGACAATTTTTTTTTTTTTT* |
| 497 | 539 | 935 | ATAT ATAATACAAACAAACTGAGTGATGGATAGTGCTTTAATGAGAA TATTTTTTTTTTTTTT* |
| 501 | 547 | 576 | ATAGAATAACAATATAAACGAACTAGATGATGGATAGTATTTTGATA TATTTTTTTTTTTTTTTT* |
| 501 | 547 | 339 | ATAGAATAACAATATAAACGAACTAGATGATGGATAGTATTTTGATA TATGCCACG |
| 501 | 547 | 55 | GTAGAATAACAATATAAACGAACTAGATGATGGATAGTATTTTGATA TATTTT |
| 508 | 547 | 36 | ATAGAATAACAATATAAACGAACTAGATGATGGATAGTAT CTTTTTTTTTTTTTTTTT |
|  |  |  |  |
| 534 | 566 | 15 | AAACGTACATATG ATCTCTTCTGCTAGTATATAAGATGATAATA ATTTTTTTTTTTTTT |
| 533 | 566 | 11 | AACGTACATATG ATCTCTTCTGCTAGTATATAAGATGATAATAT TTTTTTTTTTT |
| 535 | 566 | 89 | ATATG ATCTCTTCTGCTAGTATATAAGATGATAAT TTCTTTTTTTTTTTTTTTT |
| 533 | 566 | 72 | AAATAAACGTACATATG ATCTCTTCTGCTAGTATATAAGATGATAATAT TTTTTTTT |
| 534 | 566 | 24 | ATG ATCTCTTCTGCTAGTATATAAGATGATAATA ATTTTTTTTTTTTTTT |
|  |  |  |  |
| 535 | 578 | 13 | ATATT AACGTACATACTGTCTTCTCTATTGACATATAAGATGATAAT TTTTTTTTTTTTT |
| 543 | 580 | 39 | ATAT TAAACGTACATATTATTTTCTCTACTGATATACAAG TGATAATTTTTTTTT |
|  |  |  |  |
| 568 | 604 | 76 | ATATA TATGCAACAACAGAGATAATATTGTAGATGTATATGT GATATCGTTTTTTTTTTT |
| 569 | 600 | 78 | ATATA TAACAACAAAAGTGACATTGTAAACGTATATG ATGAGTTTT |
|  |  |  |  |
| 582 | 612 | 66 | TATT AATTGGTATGTGACAATAGAAGTGATATT TTTTTTTTT |
| 582 | 611 | 66 | AATGCAGATAATT ATTGGTGTGCAATGATAGAGGTAATATT TTTTTTTTTTTT* |
| 580 | 611 | 17 | AATGCAGATAATT ATTGGTGTGCAATGATAGAGGTAATATTGT TTTTTTTTTTTT* |
|  |  |  |  |
| 597 | 625 | 7 | A AATGCAAATAGAAGTTGGTGTGCAAT TATAGAGATGATATTTTTTTTT |
|  |  |  |  |
| 609 | 644 | 274 | ATATATAAGAATTACAATGGT GTATTAAGTGAAGTAATGTAAATGAGAATT TTTTTTTTTTTT |
| 609 | 640 | 1 | GATA GTTAGATAGAATAATGTAAGTGAAAATT TTTTTTTTTTTT |
|  |  |  |  |
| 615 | 659 | 1 | ATATATAA AGGATTACAGTGGTGATATTGAATAGAGTGATGTAAATG TTTTTTTTTTTT |
| 618 | 658 | 1,673 | ATATAT GAATTACAACGGTGATATTGAATAGAATAATGTGA TTGAAATTTTTTTTTTTTTT* |

K) Ribosomal Protein Subunit 12

| 5' | 3' | Reads | RPS12 gRNA Sequences |
| --- | --- | --- | --- |
| 43 | 78 | 12,531 | ATAT ACAACAACCATATGAAGATCATGTACGTAGAAGA TTGATATATTTTTTTTTTTTTT |
| 35 | 76 | 2,218 | ATA AACAACCGTACAGAAGTTACATATGCAGAGAAGGTGAGAT TTATTTTTTTTTTTT |
| 43 | 78 | 1,663 | ATAT ACAACAACCATATGAAGATCATGTACGTAGAAGA TTGATATATTTTTTTTTTTT |
| 38 | 78 | 423 | ATAT ATAACAACCATACAGAAATCGTATATGTGAGAGAAGTGA TTTCTTTTTTTTTTTTTTT |
|  |  |  |  |
| 63 | 109 | 5,122 | AT ATAATATAAAACAAATAAGACAGAGTGTAGATAGTAATTGTATGA TATTTTTTTTTTTT |
| 66 | 99 | 32 | AT ATAAATAAAACAAAACGTAAATGATAACTGTG AGATGATTTTTTT |
|  |  |  |  |
| 74 | 106 | 1,212 | ATAT ATATAAAACAAATAGAATAGAACGTAGATGAT TACTGTATAATTTTTTTTTTAT |
| 73 | 115 | 120 | A TATATAATAACATAAAACAAATAGAACGAGATGTAAATGATA TCTATTTTTTTATTTTT |
| 73 | 115 | 1,879 | ATA TATATAATAACATAAGACAGATAGAACGAGATGTAAATGATA TTTTCTTTTTTTTTTTTTTTT |
| 74 | 115 | 1,091 | ATA TATATAATAACATAAGACAGATAGAACGAGATGTAAATGAT TTTTTTTTTTTTT |
| 77 | 115 | 466 | ATATA TATATAATAACATAAGACAGATAGAACGAGATGTAAAT TTTTT* |
| 79 | 115 | 311 | ATA TATATAATAACATAAGACAGATAGAACGAGATGTAA TTTTTTTTTTTTTT |
|  |  |  |  |
| 96 | 121 | 233 | AATCA CGGATTTATATAGTAACGTAAAATGA TATTATTTTTTTTTTTT |
| 92 | 121 | 90 | AACTGGGC-ATCT CGGATTTGTATAGTGATATAAAGTGAATAA TTTT |
| 96 | 121 | 1,542 | ATATAAACTGTGCAATCGA TGGACTTATATAGTGATGTAAGATGA TAATTTTTTTTTTTG† |
| 96 | 121 | 1,066 | ATATAGAACTAGGCAGTCA CGGATTTGTATAGTAATGTAGAATGA TATTTTTTTTTTTTTT† |
| 93 | 121 | 793 | ATATAGAACTGGCAATTT CGGATTTATATAGTGACATGAGATAGATA TTTTTTTTTTTTTTT*† |
| 94 | 121 | 219 | ATATAGAACTGGCAATTT CGGATTTATATAGTGACATGAGATAGAT TTTTTTTTTT† |
|  |  |  |  |
| 96 | 131 | 2 | ATATAGAATTA GGCAATCGCGGATTTATATAGTAACGTAAAATGA TATTTTTTTTTT |
|  |  |  |  |
| 119 | 158 | 3 | ACT TACAATACACGTTGGTTATCGGAGTTAGGTGATTGTG ACTTATTTTTTTTTT |
|  |  |  |  |
| 139 | 170 | 44 | ATATA ACGGCATATAGTATACGTCGGTTACTAGGATTGTGTAATTTT |
| 132 | 164 | 18 | CATATAAA GGCATATAGTATACGTCGGTTACTGGGATTGTGTAATTTTTTT |
| 133 | 158 | 56 | ATACT TACAATACACGTTGGTTATCGGAGTT AGATGATTTTTTTTTTTTT |
|  |  |  |  |
| 169 | 208 | 4,724 | ATAAAT AACAACGCAATATCCGAGTAAGATTGTATAAGTGAGATAT ATTTTTTTTTTTT |
| 164 | 195 | 146 | ATAACGCAACA TCAGATGAGATTATATAAGTGAGATATG ATATATTTTTTTTTTT |
| 158 | 207 | 67 | ATAT ATAACGCAACATTCGAATGAGATTATGTAGATGAAATATGGTAT TATTTTT |
| 164 | 201 | 896 | ATA TAACATCCAAACAAGATTATATAGGTAGAGTATG ATGTATAATTTTAT_22_ |
| 156 | 207 | 192 | ATAT ACAACGTAACATTCAGATAAGATTGTGTAGATAGAATATGGTATAT TTTTTTTTTTTT |
| 158 | 207 | 104 | ATAT ACAACGTAACATTCAGATAAGATTGTGTAGATAGAATATGGTAT TTTTTT |
|  |  |  |  |
| 198 | 235 | 4,950 | ATATATAATGAC TAACTAAACTGATAAAGCAGTAGAAGAGATGATGTAAT TTTTTTTTTTT |
| 194 | 235 | 3,025 | ATATATAATGAC TAACTAAACTGATAAAGCAGTAGAAGAGATGATGTAATATTT TTTTTTTTTTT |
| 196 | 235 | 222 | TAATGAC TAACTAAACTGATAAAGCAGTAGAAGAGATGATGTAATAT ATTTTTTTTTTTTTT |
| 198 | 235 | 444 | ATATATAATGAC TAACTAAACTGATAAAGCAGTAGAAGAGACGATGTAAT TTTTTTTTTTTT* |
| 194 | 235 | 338 | ATATATAATGAC TAACTAAACTGATAAAGCAGTAGAAGAGACGATGTAATATTT TTTTTTTTTT |
| 198 | 229 | 36 | ATAACTT GACTAATAGAGTAGTGAGAGAGACAGTGTAAT TTTTGTTT |
| 200 | 235 | 25 | ATATATAATGAC TAACTAAACTGATAAAGCAGTAGAAGAGACGATGTA TTTTTTTTTTTTTTT |
| 196 | 235 | 20 | ATAATGAC TAACTAAACTGATAAAGCAGTAGAAGAGACGATGTAATAT ATTTTTTTTTTTT |
|  |  |  |  |
|  |  |  |  |
|  |  |  |  |
|  |  |  |  |
|  |  |  |  |
|  |  |  |  |
|  |  |  |  |
|  |  |  |  |
|  |  |  |  |
|  |  |  |  |
|  |  |  |  |
|  |  |  |  |
|  |  |  |  |
|  |  |  |  |
|  |  |  |  |
|  |  |  |  |
|  |  |  |  |
|  |  |  |  |
|  |  |  |  |
|  |  |  |  |
|  |  |  |  |
|  |  |  |  |
| 5' | 3' | Reads | RPS12 gRNA Sequences cont. |
| 203 | 246 | 341,382 | ATATAT ATAATGACGTAACTGAGCTAATGAGGCAATGAGAGAGATAAT ATTTTTTTTTTTTTT |
| 203 | 246 | 1,324 | ATATAT ATAATGACGTAACTGAGCTAATGAGGCAATGAGAGAAATAAT ATTTTTTTTTATTTTT |
| 205 | 246 | 1,091 | ATATAT ATAATGACGTAACTGAGCTAATGAGGCAATGAGAGAGATA TTTTTTTTTTTTT |
| 209 | 246 | 964 | ATATAT ATAATGACGTAACTGAGCTAATGAGGCAATGAGAGA TAATATTTTTTTTTTTT |
| 206 | 246 | 922 | ATATAT ATAATGACGTAACTGAGCTAATGAGGCAATGAGAGAGAT TTTTTTTTTTTTTG |
| 200 | 246 | 609 | ATAT ATAATGACATAATTAGACTGATAAGATAACGAGAAAAGTGATGTA TTTTTTTTTTTT |
| 203 | 246 | 544 | ATATAT ATAATGACGTAACTGAGCTAATGAAGCAATGAGAGAGATAAT ATTTTTTTTTTTTT |
| 203 | 246 | 530 | ATATAT ATAATGACGTAACTGAGCTAATGAGACAATGAGAGAGATAAT ATTTTTTTTTTTG |
| 208 | 246 | 484 | ATATAT ATAATGACGTAACTGAGCTAATGAGGCAATGAGAGAG TTAATATTTTTTTTTTT |
| 204 | 246 | 430 | ATATAT ATAATGACGTAACTGAGCTAATGAGGCAATGAGAGAGATAA AATTTTTTTTTTTTT |
| 203 | 246 | 370 | ATATAT ATAATGACGTAACTGAGCTAATGAGGCAATGAGAGGGATAAT ATTTTTTTTTTTT |
| 207 | 246 | 370 | ATATAT ATAATGACGTAACTGAGCTAATGAGGCAATGAGAGAGA AATATTTTTTTTTTTTT |
| 203 | 246 | 312 | ATATAT ATAATGACGTAACTGAACTAATGAGGCAATGAGAGAGATAAT ATTTTTTTTTTTT |
| 210 | 246 | 282 | ATATAT ATAATGACGTAACTGAGCTAATGAGGCAATGAGAG CGATAATATTTTTTTTTTTTCT |
| 211 | 246 | 268 | ATATAT ATAATGACGTAACTGAGCTAATGAGGCAATGAGA TTTTTTTTTTTT |
| 203 | 250 | 267 | AT ATAAATAATGACATAACTAGGTTAGTAAAGTGACGAAGAAGATAAT ATTATTTT |
| 203 | 246 | 196 | ATATAT ATAATGACGTAACTGAGTTAATGAGGCAATGAGAGAGATAAT ATTTTTTTTATTTT |
| 203 | 246 | 193 | ATATAT ATAATGACGTAACTGAGCTAATGAGGCAACGAGAGAGATAAT ATTTTTTTTTATTTTTTTTTTT |
| 205 | 246 | 177 | ATATAT ATAATGACGTAACTGAGCTAATGAGGCAATGAGAGAAATA TTTTTTTTTGTTTTT |
| 212 | 246 | 176 | ATATAT ATAATGACGTAACTGAGCTAATGAGGCAATGAG CGAGATAATATTTTTTTTTTCTTT |
| 203 | 246 | 173 | ATATAT ATAATGACGTAACTGAGCTAATAAGGCAATGAGAGAGATAAT ATTTTTTTTTTTTTT |
| 203 | 246 | 157 | ATATAT ATAATGACGTAACTGAGCTAATGGGGCAATGAGAGAGATAAT ATTTTTTTTTTTC |
| 203 | 246 | 141 | ATATAT ATAATGACGTAACTGAGCTAATGAGGCGATGAGAGAGATAAT ATTTTTTTTTGTTTTTTTTT |
| 203 | 246 | 130 | ATATAT ATAATGACGTAACTAAGCTAATGAGGCAATGAGAGAGATAAT ATTTTTTTTTTTTT |
| 203 | 246 | 119 | ATATAT ATAATGACGTAACTGAGCTAATGAGGTAATGAGAGAGATAAT ATTTTTTTTTTTT |
| 203 | 246 | 117 | ATATAT ATAATGACGTAACTGAGCCAATGAGGCAATGAGAGAGATAAT ATTTTTTTTTATTTTTT |
| 203 | 246 | 116 | ATATAT ATAATGACGTAACTGGGCTAATGAGGCAATGAGAGAGATAAT ATTTTTTTTTTTTT |
| 203 | 245 | 56 | ATAAAAT TAATGACATAACTAAATTGATAGGGTAATGAGAGAGATAAT ATTTTTTTGT |
|  |  |  |  |
| 234 | 264 | 35 | TAG TGCCTTCTATAGTAGATGATGATATA TGATTTTTTTTTTTTTT |
| 234 | 280 | 24 | ATATA AGATCAACAAAACTGCCATTTTCTGTAGTAAGTGATGATATA TTTTTTTTTATTTT |
| 248 | 281 | 14 | ATA TAAATCAACAGAACTGCCATCTTTTGTAGTA TAGTGATATAATTTTTTTTTTTTT |
| 234 | 280 | 434 | ATATA AGATCAACAAAACTGCCATTTTCTATAGTGAGTGATGATATA TTTTTTTTTTTTTTTT |
| 248 | 282 | 270 | ATAT GTAAATCAACAGAACCGTCATCTTTTGTAGTA TAGTGATATAATTTTTTTTTTTT |
|  |  |  |  |
| 288 | 322 | 16,349 | ATA TACAATACGTGTATGATATTTTATACT AGGTAGATCAGTGAAATT TTTTTTTTTTTT |
| 267 | 322 | 10 | ATA TACAATACGTGTATGATATTTTATACTGGGTAGATCAGTGAAATT TTTTTTT |
| 269 | 308 | 3,731 | ATA TATAATACTTTACATCGGGTAAATTGACGAGA ACATGATTTTTTTTTTT |
| 288 | 322 | 909 | ATA TACAATACGTGTGTAATATTTTATACT AGGTAGATCAATGAAATTTTTTTTTTTTTTT |
|  |  |  |  |
| 309 | 349 | 128 | AAATAT AACATATCTTATATCTGAATCTAACTTGTAATATGTG AATTTTTTTTTTTTTTTT |
| 309 | 336 | 195 | AAATAAAACATATCTGAT TCTAAATCTAACTTGTAATGTGTG AATTTTTTATTTTTTTTTTTTTTTTTT† |

*Indicates that the tail sequence was shortened where random nucleotides after the poly U tail had been indicated.

†Indicates that the gRNA was identified under conditions of reduced stringency.
